# How to train your neuron: developing a detailed, up-to-date, multipurpose model of hippocampal CA1 pyramidal cells

**DOI:** 10.3389/fncom.2026.1897214

**Published:** 2026-09-22

**Authors:** Luca Tar, Sára Sáray, Máté Mohácsi, Tamás F. Freund, Szabolcs Káli

**Affiliations:** 1 HUN-REN Institute of Experimental Medicine, Budapest, Hungary; 2 Faculty of Information Technology and Bionics, Pázmány Péter Catholic University, Budapest, Hungary

**Keywords:** compartmental modeling, computer simulations, dendritic spines, hippocampus, model validation, parameter optimization

## Abstract

Anatomically and biophysically detailed models of neurons have been widely used to study information processing in these cells. Most studies focused on understanding specific phenomena, while more general models that aim to capture various cellular processes simultaneously remain rare even though such models are required to predict neuronal behavior under more complex, natural conditions. In this study, we aimed to develop a detailed, data-driven, general-purpose biophysical model of hippocampal CA1 pyramidal neurons. We leveraged extensive morphological, biophysical and physiological data available for this cell type, and established a systematic workflow for model construction and validation that relies on our recently developed software tools. The model is based on a high-quality morphological reconstruction and includes a diverse curated set of ion channel models. After incorporating the available constraints on the distribution of ion channels, the remaining free parameters were optimized using the Neuroptimus tool to fit a variety of electrophysiological features extracted from somatic whole-cell recordings. Validation using HippoUnit confirmed the model’s ability to replicate key electrophysiological features, including somatic voltage responses to current input, the attenuation of synaptic potentials and backpropagating action potentials, and nonlinear synaptic integration in oblique dendrites. Our model also included active dendritic spines, modeled either explicitly or by merging their biophysical mechanisms into those of the parent dendrite. We found that many aspects of neuronal behavior were unaffected by the level of detail in modeling spines, but modeling nonlinear synaptic integration accurately required the explicit modeling of spines. Our data-driven model of CA1 pyramidal cells matching diverse experimental constraints is a general tool for the investigation of the activity and plasticity of these cells and can also be a reliable component of detailed models of the hippocampal network. Our systematic approach to building and validating multi-purpose models should apply to other cell types as well.

## Introduction

In the last few decades, anatomically and biophysically detailed neuronal models have become essential tools in neuroscientific research. Such models can be used to examine the function and behavior of specific types of neurons, to explore the various mechanisms behind the observed electrophysiological behavior, and to predict neuronal behavior and function under circumstances that are difficult to study directly in experiments (Almog and Korngreen, 2016; Almog and Korngreen, 2014; Hay et al., 2011; Schutter and Bower, 1994; Poirazi et al., 2003; Migliore et al., 1999; Migliore et al., 2018; Káli and Freund, 2005), In addition, such detailed cellular models are the fundamental building blocks of detailed network models (Bezaire et al., 2016; Markram et al., 2015; Traub et al., 2005; Schneider et al., 2012; Romani et al., 2024).

Recently, it has become standard to support new experimental findings with models tuned to replicate observed behaviors; however, many earlier models had significant limitations. Often, these models were fitted to a narrow set of experimental conditions, and there is typically very little information about how these models work outside the scope of the study they were developed for Almog and Korngreen (2016). Such models are frequently underconstrained, as multiple parameter combinations can fit the limited data equally well, potentially resulting in unrealistic parameter values and incorrect predictions under different conditions. Additionally, parameters were often adjusted manually, and modifications to accommodate new data were rarely assessed systematically, limiting model reusability and leading to unnecessary duplication of effort.

More recently, several tools have been developed that allow the automated determination of model parameters using a potentially large set of electrophysiological features (Mohácsi et al., 2024; Geit Wv et al., 2016) and support the systematic validation of the resulting models against experimental data (Sáray et al., 2021). Using the HippoUnit model validation tool, Sáray et al. (2021) compared the behavior of several rat CA1 pyramidal cell (PC) models available in the ModelDB database against data from various experimental paradigms. Such comparisons help researchers determine which existing model provides the best fit to the data in the domains they care about and whether they can reuse existing models or should build their own. However, the overall conclusion of this study was that although several models successfully capture certain aspects of the electrophysiological behavior of CA1 PCs (typically those that the models were originally built to replicate), none of the existing models can simultaneously capture all the relevant behaviors of CA1 PCs covered by HippoUnit, and a thoroughly validated, accurate general-purpose model of this cell type is currently missing.

In the current work, we developed and validated morphologically and biophysically detailed models of CA1 pyramidal cells using our previously developed tools for parameter optimization and model validation. Our goal was to create models consistent with the widest possible range of experimental observations, making them broadly applicable for various modeling studies. CA1 pyramidal cells are among the most extensively studied neuron types, with abundant experimental data available on their physiological behavior—including both somatic and dendritic responses to current injections and synaptic inputs—as well as detailed information on ion channel distribution and kinetics. This wealth of data provided a strong foundation for developing a detailed, widely usable model and for demonstrating methods that can be applied to other cell types.

A crucial aspect of developing an up-to-date biophysical model of CA1 PCs was the review and update of the ion channel models in light of recent scientific advances. Over the past decade, improvements in assessing the molecular composition and quantification of ion channels in cell membranes have provided computational neuroscientists with new quantitative data on channel distributions, which can be incorporated as constraints during parameter fitting. Additionally, more experimental data are now available on ion channel kinetics in pyramidal neurons, including regional differences within dendrites. These advances enabled us to curate a new set of ion channel models specifically for CA1 pyramidal cells, significantly enhancing our model’s performance.

Accurate modeling of CA1 pyramidal neurons requires consideration of dendritic spines, which receive most excitatory synaptic input, function as distinct electrical and biochemical compartments, and play key roles in signal integration and plasticity. Many previous models of CA1 pyramidal cells either ignored spines or represented them in a highly simplified manner. In this study, we aimed to develop a fully active model that includes spines, enabling analysis of the contributions of nonlinear processes in spines and dendrites to signal integration and action potential generation.

A major challenge in model development is that detailed modeling of dendritic spines greatly increases computational complexity. Fortunately, recent advances in modeling tools that support parallel execution allow efficient simulation of complex neuronal models on modern computing clusters and supercomputers. To further address computational demands, we also explored simplified models that do not explicitly represent dendritic spines but instead adjust membrane properties—including voltage-gated conductance densities—using a surface-correction factor (F-factor) that accounts for the additional membrane area contributed by spines. By employing these approaches, we were able to compare different methods for incorporating dendritic spines into our models and to identify which aspects of neuronal physiology depend on the compartmentalization of specific mechanisms within spines. This comparison provides guidance for researchers in selecting the most appropriate strategy for modeling spines in future studies.

## Methods

### Data

To construct realistic models of hippocampal CA1 pyramidal neurons and validate their somatic electrophysiological behavior, we used somatic whole-cell patch-clamp recordings from rat CA1 PCs. The data were provided by Judit Makara and were previously described in Sáray et al. (2021). The recordings were obtained in current-clamp mode and contain the somatic membrane potential responses of 6 CA1 PCs (with 3 measurements from each cell) to somatic step current injections of 300 ms duration and ten different amplitudes (−0.25, −0.2, −0.15, −0.1, −0.05, 0.05, 0.1, 0.15, 0.2, and 0.25 nA). One of the 6 cells fired doublets (which can be considered as “mini-bursts”) in response to 0.1 nA current injections, and the voltage response also started with a burst at higher current injection amplitudes. Since it displayed a qualitatively different voltage response from the other cells, we excluded it from the target data for the current study, although its behavior could be fitted separately (the voltage response of the excluded cell can be seen in Supplementary Figure 1). A typical experimental voltage response can be seen in Figure 1C. From the raw voltage traces, we extracted electrophysiological features using BluePyEfe (https://github.com/BlueBrain/BluePyEfe) and the eFEL feature extraction library (https://github.com/BlueBrain/eFEL) (Bologna et al., 2021).

**Figure 1:**
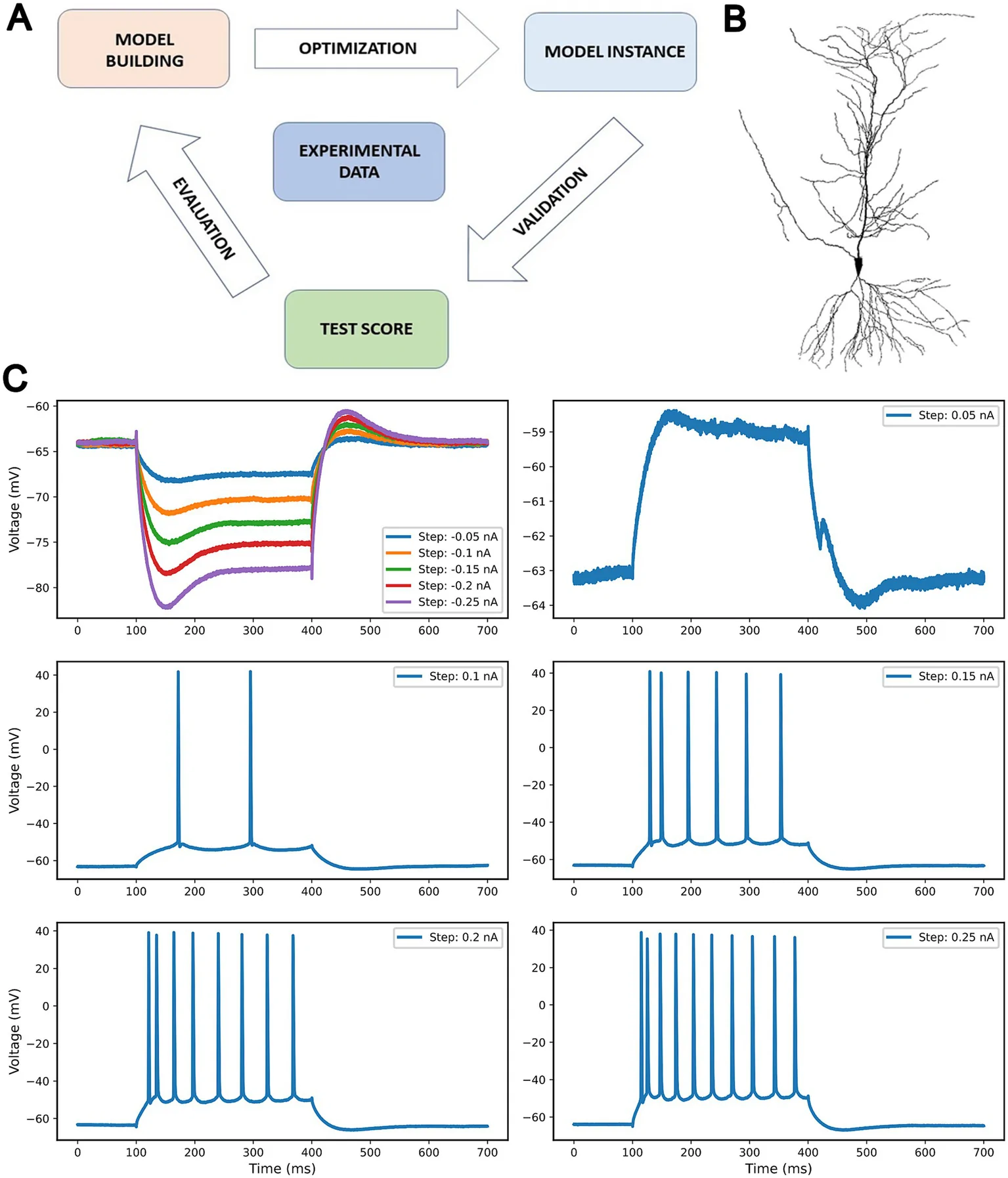
Modeling workflow. **(A)** Schematic representation of the model development process. First, we define a family of models based on a reconstructed morphology and a set of biophysical mechanisms. Next, we optimize the unknown parameters of the model using electrophysiological data; running the optimization algorithm multiple times with different random seeds results in several (typically 20) model instances. Then we validate these models using the automated tests implemented by the HippoUnit framework, which provides test scores as well as further visual and quantitative diagnostics. We evaluate the candidate models based on the scores and figures, modify or expand the models accordingly, and then repeat the entire process. Between iterations the set of included mechanisms, the optimized parameters and their boundaries may also change. **(B)** The morphology of the model, with dendritic reconstruction and soma parameters from Megías et al. (Megías et al., 2001), and axonal reconstruction from Bianchi et al. (Bianchi et al., 2012). **(C)** An example of the experimental voltage traces resulting from our standard current step protocol that we used for the extraction of electrophysiological features. We extracted features and then optimized our parameters for 5 negative current steps and 5 positive current steps that lasted for 300 ms.

For those features that average over all the action potentials we always excluded the first action potential because of a systematic error in the detection of action potential initiation near the beginning of the current step. Sag features were evaluated only for negative amplitudes, voltage deflection features were only considered at non-spiking amplitudes, while spiking features were evaluated for current steps where spikes could be observed in the experimental data.

For parameter fitting, we selected a set of eFEL features that best describe the somatic behavior of the cell with minimal redundancy. The selected features are listed in Table 1. For validation of somatic behavior, a wider range of features was used, as described in Sáray et al. (2021).

**Table 1:** The optimized eFEL features during the parameter fitting process with the current steps where they were evaluated.

Feature name from eFEL	Feature description	Fitted current steps (nA)
Spikecount	Number of spikes during stimulus	−0.05, −0.1, −0.15, −0.2, −0.25, 0.05, 0.1, 0.15, 0.2, 0.25
inv_first_ISI	Reciprocal of first interspike interval	0.15, 0.2, 0.25
inv_second_ISI	Reciprocal of second interspike interval	0.2, 0.25
inv_third_ISI	Reciprocal of third interspike interval	0.2, 0.25
inv_fourth_ISI	Reciprocal of fourth interspike interval	0.2, 0.25
inv_fifth_ISI	Reciprocal of fifth interspike interval	0.2, 0.25
inv_last_ISI	Reciprocal of last interspike interval	0.15, 0.2, 0.25
inv_time_to_first_spike	Reciprocal of time to first spike	0.15, 0.2, 0.25
steady_state_voltage	Average voltage after stimulus	−0.05, −0.1, −0.15, −0.2, −0.25, 0.05, 0.1, 0.15, 0.2, 0.25
voltage_base	Average voltage during the last 10% of time before the stimulus	−0.05, −0.1, −0.15, −0.2, −0.25, 0.05, 0.1, 0.15, 0.2, 0.25
voltage_deflection	Difference between the voltage base and the steady-state voltage at the end of the stimulus	−0.05, −0.1, −0.15, −0.2, −0.25
voltage_deflection_begin	Difference between the voltage base and the mean voltage in the early phase (5 to 15% of duration) of the stimulus	−0.05, −0.1, −0.15, −0.2, −0.25
AHP_depth_abs	Absolute value of the depth of afterhyperpolarization	0.15, 0.2, 0.25
AHP_time_from_peak	Time from the peak of the AP to the minimum of the afterhyperpolarization	0.15, 0.2, 0.25
AP2_amp	Amplitude of second AP	0.15, 0.2, 0.25
AP_amplitude	Average height of APs	0.15, 0.2, 0.25
AP_begin_voltage	Threshold of AP initiation	0.15, 0.2, 0.25
AP_duration_half_width	Half-width of the AP	0.15, 0.2, 0.25
AP_fall_time	Time from the peak of the AP to the end of the AP	0.15, 0.2, 0.25
AP_rise_time	Time from the AP threshold to the peak of the AP	0.15, 0.2, 0.25
AP_last_amp	Amplitude of the last AP	0.15, 0.2, 0.25
time_to_last_spike	Time to the last spike	0.15, 0.2, 0.25
sag_amplitude	Difference between the minimal voltage and the steady state voltage at the end of the stimulus	−0.25
sag_ratio1	Ratio between sag amplitude and maximal hyperpolarization from voltage base	−0.25

### Model building

#### Morphology

We used morphology pc1a from Megías et al. (2001), which had reconstructed soma and dendrites in detail, and we also added a few axonal compartments from the model of Bianchi et al. (2012). The reconstructed morphology can be seen in Figure 1B. Megías et al. (2001) also give detailed information about the number of dendritic spines of each dendritic region, from which we calculated the spine density (number/μm length) for the basal dendrites (2.85), trunk (4.52), oblique (3.52) and tuft dendrites (0.56), and the distance between spines on a dendritic section was 1/density in the corresponding region. The dimensions of all dendritic spines were modelled according to Harnett et al. (2012) (spine neck length: 1.58 μm, spine neck diameter: 0.077 μm, spine head length and diameter: 0.5 μm).

With this method, we can explicitly model any number of spines at any location, taking the spine density of the region into account, including modelling all the possible spines on the morphology, resulting in a total of 29,602 dendritic spines. However, modelling all dendritic spines greatly increases the computational cost of the model, and therefore, in most of our simulations, we used a method that takes spines into account in a computationally more efficient way.

There are several different approaches to take the dendritic spines into account without explicitly simulating them, but the most principled and computationally efficient way is the correction of the appropriate biophysical parameters with the surface factor (F-factor). The F-factor is defined as the relative increase in the membrane area of a dendritic section due to the presence of spines. The spines consist of a spine neck, which is modeled as a cylinder, and a spine head, which can be modeled as a sphere or as a cylinder. We note that if both the length and the diameter of the cylinder are equal to the diameter of the sphere, they have equal surface areas (excluding the bases of the cylinder). Therefore, for convenience, we decided to model the spine head as a cylinder. From this, the surface of the spine can be obtained as follows (Equations 1–7):


$$\text{surfac}e_{\text{spine}}=\text{surfac}e_{\text{sneck}}+\text{surfac}e_{\text{shead}}$$ (1)

$$\text{surfac}e_{\text{sneck}}=\mathrm{dia}m_{\text{sneck}}\times \mathrm{le}n_{\text{sneck}}\times \pi$$ (2)

$$\text{surfac}e_{\text{shead}}=\mathrm{dia}m_{\text{shead}}\times \mathrm{le}n_{\text{shead}}\times \pi$$ (3)

$$\begin{array}{l} \text{surfac}e_{\text{spine}}=(\mathrm{dia}m_{\text{sneck}}\times \mathrm{le}n_{\text{sneck}}\times \pi )\\ +(\mathrm{dia}m_{\text{shead}}\times \mathrm{le}n_{\text{shead}}\times \pi ) \end{array}$$ (4)

The surface area of a dendrite of length $L_{\text{dend}}$ and $\mathrm{dia}m_{\text{dend}}$ is


$$\text{surfac}e_{\text{dendrite}}=\mathrm{dia}m_{\text{dend}}\times L_{\text{dend}}\times \pi$$ (5)

and the total surface of the spines on that dendrite is:


$$\text{surfac}e_{\mathrm{all}\;\text{spines}}=\text{surfac}e_{\text{spine}}\times \text{spine density}\times L_{\text{dend}}$$ (6)

From this the F-factor for that dendritic segment is:


$$\begin{array}{l} F-\text{factor}=(\text{surfac}e_{\mathrm{all}\;\text{spines}}+\text{surfac}e_{\text{dendrite}})\;/\text{surfac}e_{\text{dendrite}}\;\\ =\frac{\begin{array}{l} \left(\mathrm{dia}m_{\text{sneck}}\times \mathrm{le}n_{\text{sneck}}+\mathrm{dia}m_{\text{shead}}\times \mathrm{le}n_{\text{shead}}\right)\\ \times \text{spine density}+\mathrm{dia}m_{\text{dend}} \end{array}}{\mathrm{dia}m_{\text{dend}}} \end{array}$$ (7)

Then, instead of modeling each spine explicitly, their effect on electrical signaling in the dendrites can be taken into account by multiplying the leak conductance and the membrane capacitance by the corresponding F-factor. A similar approach can be applied to active conductances that are present on the spines (Rapp et al., 1992). The F-factor was calculated independently for every section of the dendritic tree. For every dendritic section where we did not explicitly model them, we take the spines into account by the F-factor. For those dendrites that wear explicitly modeled spines (any number), the F-factor was calculated with the exclusion of the membrane area of the modeled spines.

#### Passive parameters

The specific membrane capacitance was set to 1 μF/cm^2^, which corresponds to the standard experimentally measured value for the neuronal membrane and was later multiplied by the F-factor on the affected dendritic sections. The specific axial resistance (Ra) of the model was set to 50 Ohm×cm, which is lower than the values typically used in previous models of CA1 PCs. This lower Ra is in alignment with recent model-based estimates of passive parameters of CA1 PCs (Terbe et al., 2022) and was essential for the model to capture a wide variety of experimental data on signal propagation and integration.

#### Channel models

The model contains 13 active voltage- and/or calcium-gated ion channels, and occasionally 2 or 3 versions of one type of ion channel when the kinetics of the ion channels are dependent on their distances from the soma or on the dendritic region in which they are located. Our channel models are diverse in implementation and in their sources, but every one of them was carefully evaluated in the light of the newest data that can be found in the literature about their kinetics and were modified accordingly when necessary.

The current that flows through an ion channel at a given membrane potential is given by Equation 8:


I=g(V−e) (8)

where V is the membrane potential, e is the equilibrium potential or reversal potential of the given ion, and g is the conductance of the ion channel. The voltage and/or calcium-dependent g is the product of the theoretical maximal conductance of the ion channel and gating variables independent from each other, corresponding to the number of activation and inactivation gates that the given ion channel possesses. A gating variable X, can be described with Equations 9–11 based on the Hodgkin-Huxley model:


$$\frac{\mathrm{dX}}{\mathrm{dt}}=\frac{X_{\infty }(V)-X}{\tau_{X}(V)}$$ (9)

where $X_{\infty }$ is the steady-state value of X , $\tau_{X}$ is the time constant, and both are dependent on the voltage (or the calcium concentration in some cases).

$X_{\infty }\;$ and $\tau_{X}\;$ can both be described by several different formalisms. In the more traditional Hodgkin-Huxley equations, they are defined in terms of the forward (α) and backward (β) rates of channel opening and closing. In this formalism


$$X_{\infty }=\frac{\alpha }{\alpha +\beta }$$ (10)

$$\tau_{X}=\frac{1}{\alpha +\beta }$$ (11)

Although for some ion channels we used models that employ this kind of equation, experimental data regarding ion-channel kinetics rarely provide these rates directly. Instead, voltage-clamp experiments typically measure the voltage- and /or calcium concentration-dependent steady state activation and inactivation as well as the associated time constants. For easier parameterization and to adjust our channels more directly to the experimental data, we described most of our channels using the formalism introduced by Borg-Graham (1999), where (Equations 12, 13):


$$X_{\infty }=\frac{1}{1+e^{-\frac{V-\text{Vhalf}}{k}}}$$ (12)

$$\tau =\tau_{0}+\frac{1}{G*e^{\gamma \times \frac{V-\text{Vhalf}}{k}}+G*e^{(\gamma -1)\times \frac{V-\text{Vhalf}}{k}}}$$ (13)

where Vhalf is the voltage where X is half-activated at steady state, 1 / (4 k) is the slope of the steady-state activation curve at Vhalf , G is a rate coefficient and γ is an asymmetry parameter (where 0 = < γ <= 1) for the voltage-dependent part of τ , and $\tau_{0}\;$ is the voltage-independent part of τ . Then the conductance g can be calculated via Equation 14:


$$g=g_{\max}\times X$$ (14)

if the channel has only a single activation gate. Additional gates (for activation and inactivation) can be described as multipliers of the right-hand side of this equation.

The models of calcium channels use the Goldman-Hodgkin-Katz equation to describe the current, which gives a better fit for currents mediated by calcium ions than the traditional conductance-based Hodgkin-Huxley formalism. In this description, instead of the conductance, the theoretical maximal permeability is used, and the activation and inactivation curves are multiplied by the GHK (Equations 15–18) instead of the driving force.


ghk=0.001×2×F×(eci−eco) (15)

$$\mathrm{eci}={\left[Ca^{2+}\right]}_{\text{inside}}\times \frac{z}{e^{-z}-1}$$ (16)

$$\mathrm{eco}={\left[Ca^{2+}\right]}_{\text{outside}}\times \frac{z}{e^{z}-1}$$ (17)

z=0.001×2×F×V/(R×(celsius+273.15)) (18)

where F is the Faraday constant, and R is the gas constant, and celsius is the temperature of the model is in degrees Celsius. From this we can calculate the current (Equation 19):


$$I=\text{permeability}\times X\times \mathrm{ghk}(V,{\left[Ca^{2+}\right]}_{\text{inside}},{\left[Ca^{2+}\right]}_{\text{outside}})$$ (19)

The value of ${\left[Ca^{2+}\right]}_{\text{outside}}$ is the default value for the extracellular calcium concentration of the Neuron simulator (2 mM) while the ${\left[Ca^{2+}\right]}_{\text{inside}}$ is determined by the model of the calcium dynamics (see later). Since we tried to find and, in many cases, create ion-channel models that best describe the latest experimental results, differences from these equations can occur. In those cases, we state the equations and parameters in the description of the ion channel in question.

In the following description of the channel models, the voltage dimension values are expressed as millivolts (mV) while the time dimension values are in milliseconds (ms).

##### Hyperpolarization-activated mixed cation (Ih) current

The model of the h-current was based on the observations and measurements of Zemankovics et al. (2010). Since the earlier data of Magee (1998) indicated that the proximal and distal channels differ in the voltage-dependence of their activation kinetics, two different channel models were incorporated into the model and distributed throughout the dendritic arbor in a non-uniform fashion (Magee, 1998).

The gating of this ion channel is described by Equations 20–22. For the proximal H-current:


$$X_{\inf}=1/(1+e^{0.3\times (V+83)})$$ (20)

For the distal H-current:


$$X_{\inf}=1/(1+e^{0.3\times (V+89)})$$ (21)

and for both channels the τ was:


$$\tau =\frac{e^{0.033\times (V+75)}}{11*(1+e^{0.083\times (V+75)})}$$ (22)

To determine the exact amount of Ih in the model, a ratio parameter was fitted during the optimization process that served as a multiplier in each dendritic segment. With this ratio parameter, the maximal conductance is described by Equations 23–28 as follows. From 0 to 100 μm distance from the soma:


$$\mathrm{gma}x_{\text{prox}}=(\text{ratio}\times 2\times 10^{-5})\times (1+0.02\times \mathrm{dis}t_{\text{soma}})$$ (23)

$$\mathrm{gma}x_{\text{dist}}=0$$ (24)

From 100 μm to 400 μm:


$$\mathrm{gma}x_{\text{prox}}=0$$ (25)

$$\mathrm{gma}x_{\text{dist}}=(\text{ratio}\times 2\times 10^{-5})\times (1+0.02\times \mathrm{dis}t_{\text{soma}})$$ (26)

and further from 400 μm:


$$\mathrm{gma}x_{\text{prox}}=0$$ (27)

$$\mathrm{gma}x_{\text{dist}}=0.00018\times \text{ratio}$$ (28)

##### Sodium channel

The model has 3 types of sodium channels that have modified kinetics for the soma, axon, and dendrites.

The sodium current was calculated according to Equation 29:


$$I_{\mathrm{Na}}=\text{gmax}\times X^{3}\times Y\times Z\times (V-e_{\mathrm{Na}})$$ (29)

where gmax is the maximal conductance of the channel, X is the gating variable for activation, Y is the gating variable for fast inactivation, Z is the gating variable for slow inactivation, V is the voltage, and e_Na_ is the reversal potential of the sodium channel. X and Y were described in the Borg-Graham formalism stated above (Equations 12, 13). The steady-state half-activation and half-inactivation were fitted parameters. To ensure that the action potentials are initiated in the axon, both the half-activation and half-inactivation were more hyperpolarized by 3 mV than those of the sodium channels in the soma and dendrites (Lőrincz and Nusser, 2010).

The characteristics of the slow inactivation gate are based on data from Colbert et al. (1997) and Jung et al. (1997) and gating is described by Equations 30–32. In particular,


$$Z_{\inf}=(1+0.7\times e^{\frac{V+30}{2}})/(1+e^{\frac{V+30}{2}})$$ (30)

for the somatic sodium channels, and


$$Z_{\inf}=(1+0.2\times e^{\frac{V+30}{2}})/(1+e^{\frac{V+30}{2}})$$ (31)

for the dendritic sodium channels. The expression for τ is the same for both somatic and dendritic channels:


$$\tau_{Z}=2+(5000\times e^{0.09\times (V+45)-2})/(1+e^{0.45\times (V+45)})$$ (32)

The time constants were provided for CA1 pyramidal neurons by Martina and Jonas (1997).

The somatic density of sodium channels was a fitted parameter. The axonal sodium channel density was 40 times the somatic density, while on the oblique and basal dendrites it was 2/3 of the somatic density, and on the tuft dendrites it was 1/3 of the somatic density, based on the data of Lőrincz and Nusser (2010). On the apical trunk, the somatic density decreased with distance according to Equation 33


$$\mathrm{gma}x_{\mathrm{Na}\_\text{dend}}=\mathrm{gma}x_{\mathrm{Na}\_\text{soma}}\times (1-0.002\times \mathrm{dis}t_{\text{soma}})\;$$ (33)

##### Potassium channels

###### Delayed rectifier potassium channel

Delayed-rectifier potassium channels contribute significantly to action potential repolarization and therefore have a major impact on the shape and height of the APs, along with the sodium channels (Chen and Johnston, 2004). They were modeled according to the Borg-Graham formalism described above (Equations 12, 13). The parameters of the equations can be found in Table 2. Although this channel can be inactivated according to experimental data, this process is so slow that it is not expected to be significant under physiological conditions and was therefore not modeled.

**Table 2:** Parameters of the ion channels present in the model whose gating is described by the Borg-Graham formalism.

Ion channel	Maximal conductance (S/cm^2^)	Steady-state half activation (mV)	Slope of the steady-state activation (mV)	tau0 of activation (ms)	Asymmetry parameter of the activation tau	kt of activation (1/ms)	Power of activation	Steady-state half inactivation (mV)	Slope of the steady-state inactivation (mV)	tau0 of inactivation (ms)	Asymmetry parameter of the inactivation tau	kt of inactivation (1/ms)	Power of inactivation	Reversal potential (mV)
Somatic sodium channel	Fitted parameter	Fitted parameter	7	0.02	0.45	20	3	Fitted parameter	−2	0.5	0.2	0.1	1	55
Dendritic sodium channel	Fitted parameter	Fitted parameter	7	0.02	0.45	20	3	Fitted parameter	−2	0.5	0.2	0.1	1	55
Axonal sodium channel	Fitted parameter	Fitted parameter-3	7	0.02	0.45	20	3	Fitted parameter-3	−2	0.5	0.2	0.1	1	55
Delayed-rectifier potassium channel	Fitted parameter	0	9	2	0.9	0.05	4	-	-	-	-	-	-	−80
Proximal A-type potassium channel	0.0035 (baseline)	−9	22	1	-	-	3	−56	1/125	× × ××			1	−80
Distal A-type potassium channel	0.0035 (baseline)	−18	18	1	-	-	3	−56	1/125	× × ××			1	−80
M-type potassium channel	Fitted parameter	−56	−7	5	0.5	0.01	2	-	-	-	-	-	-	−80
D-type potassium channel	Fitted parameter	−65	10	2	-	-	3	−100	−10	1	0.5	0.006	1	-
BK-type potassium channel	Fitted parameter	−28.9	6.2	× × ××			3	−32	5.8	× × ××			1	-
Proximal H-current	0.00002 (baseline)	−83	1/300	× × ××			1	-	-	-	-	-	-	−30
Distal H-current	0.00002 (baseline)	−89	1/300	× × ××			1	-	-	-	-	-	-	−30
	Permeability (cm/s)													
L-type calcium channel	0.0004 (baseline)	× × ××					2	-	-	-	-	-	-	GHK
N-type calcium channel	Fitted parameter	−11	7	0	0.2	5	2	−75	−6.5	100	0.5	1	1	GHK
T-type calcium channel	Baseline varies depending on region	−44	12	1.275	0.4686	0.0836	3	−53.4	−11	20	× × ××		1	GHK
R-type calcium channel	Fitted parameter	−29	9.6	5.1	-	-	3	−33	−17	22	× × ××		1	GHK

Cells containing × × × × have their equations in different formalisms; for these equations and parameter values, see the corresponding parts of methods.

###### M-type potassium channel

The M-type potassium channel has roles in generating the somatic medium afterhyperpolarization, preventing bursting, and generating subthreshold resonance in the theta frequency range. It also plays a major role in determining the action potential threshold and suppressing the afterdepolarization.

We used the channel model from Hönigsperger et al. (2015). The channel model uses the standard Borg-Graham formalism (Equations 12, 13). The parameters of the equations can be found in Table 2. We used the same maximal conductance on the soma and the dendrites, which was a fitted parameter, while on the axon, the maximal conductance was 4 times higher than the somatodendritic value (Shah et al., 2011).

###### A-type potassium channels

A-type potassium channels play key roles in the regulation of dendritic excitability (Hoffman et al., 1997; Johnston et al., 2000), but they also contribute to action potential repolarization (Kim et al., 2005) and influence subthreshold neuronal behavior (Johnston et al., 2000). Our model of the transient A-type potassium channel was based on the observations of Hoffman et al. (1997). They found that the voltage dependence of its activation was different in somatic and dendritic regions of the cell. We implemented two types of this channel, one for the soma and the proximal dendritic regions, and one for the distal dendritic regions. The voltage-dependent activation and inactivation of K(A) in the model were also based on data from Hoffmann et al., but we allowed some flexibility in a few specific parameters to make sure that this channel could fulfil all the diverse roles that are indicated above. In particular, the voltage dependence of the steady-state activation and inactivation of the channel was described with the Borg-Graham formalism (Equations 12, 13), and its baseline parameters can be found in Table 2. However, we allowed the slope of voltage-dependent K(A) activation to change during parameter optimization, and we also introduced an additional free parameter that shifted the half-activation and half-inactivation voltage by equal amounts.

The τ of the channel activation was set to a voltage-independent constant of 1 ms (Hoffman et al., 1997), while according to the same article, the time constant of inactivation above -30 mV was (Equation 34)


$$Y_{\tau }=0.00026\times (V+30\;\mathrm{mV})+5\;\mathrm{ms}\;$$ (34)

and below −30 mV (Equation 35)


$$Y_{\tau }=5\;\mathrm{ms}$$ (35)

Hoffmann et al. also stated that the maximal conductance of the A-type potassium channels is linearly increasing with the distance from the soma up until 400 μm. Since we have no data further from that point, we set the maximal conductance constant in the distal dendrites.

The model follows this inhomogeneous distribution according to the rules below (Equations 36–43).

On the soma, we have


$$\mathrm{gma}x_{\text{prox}}=0.0035\times KA_{\text{ratio}}$$ (36)

$$\mathrm{gma}x_{\text{dist}}=0$$ (37)

from the soma to 100 μm distance


$$\mathrm{gma}x_{\text{prox}}=0.0035\times KA_{\text{ratio}}\times (1+(0.0157\times \mathrm{dis}t_{\text{soma}}))$$ (38)

$$\mathrm{gma}x_{\text{dist}}=0$$ (39)

from 100 μm to 400 μm


$$\mathrm{gma}x_{\text{prox}}=0$$ (40)

$$\mathrm{gma}x_{\text{dist}}=0.0035\times KA_{\text{ratio}}\times (1+(0.0157\times \mathrm{dis}t_{\text{soma}})$$ (41)

and above 400 μm


$$\mathrm{gma}x_{\text{prox}}=0$$ (42)

$$\mathrm{gma}x_{\text{dist}}=0.0255\times KA_{\text{ratio}}$$ (43)

The value 0.0035 from the maximal conductance comes from Kerti et al. (2011), and $KA_{\text{ratio}}\;$ is a fitted parameter.

###### D-type potassium channel

The D-type potassium channel is present on the soma and the axon, and its kinetics were carefully examined and adjusted to the literature to get the delay at the beginning of the current step to the first action potential, which can be observed in the experimental data. The channel model is defined by the Borg-Graham formalism (Equations 12, 13), and its parameters can be found in Table 2.

This channel model originates from Golomb et al. (2007), but the steady-state half-activation and half-inactivation voltages were changed from −60 to −65 mV and from −90 to −100 mV, respectively, to optimally capture both voltage-clamp and current-clamp data from hippocampal pyramidal neurons (Chen and Johnston, 2004; Storm, 1990; Storm, 1988). The parameter gkd, which is the maximal conductance of the channel, is a fitted parameter.

###### Big-conductance (BK) calcium- and voltage-dependent potassium channel

Big-conductance calcium and voltage-dependent potassium channels are located on the soma, co-localized with N-type calcium channels (Marrion and Tavalin, 1998), so in the model, it takes its calcium input solely from what comes in through the N-type calcium channels. It has a voltage-dependent activation and inactivation and a calcium-dependent component that are modelled by three separate gates. The steady-state activation and inactivation of the voltage-dependent gates were described with the Borg-Graham formalism (Equation 12, parameters in Table 2), while the time constants were described by similar but differently parameterized formulae (Equations 44–45):


$$\tau_{x}=0.505+1/(e^{\frac{V-33.3}{-10}}+e^{\frac{V+86.4}{10}})$$ (44)

$$\tau_{y}=1.9+1/(e^{\frac{V-54.2}{-12.9}}+e^{\frac{V+48.5}{5.2}})$$ (45)

The calcium-dependent gate evolved according to Equations 46, 47:


$$z^{\prime }=(z_{\infty }-z)/\tau_{z}$$ (46)

$$z_{\infty }=\frac{1}{1+z_{\text{coeff}}/[Ca^{2+}]}$$ (47)

where $z_{\text{coeff}}\;$ = 0.001 mM, $\tau_{z}$ = 1 ms. From this the potassium current carried by this channel is given by Equation 48:


$$I_{k}=\text{gbar}\times X^{3}\times Y\times z^{2}\times (V-e_{k})$$ (48)

###### Small-conductance (SK) calcium-dependent potassium channel

Small-conductance calcium-dependent potassium channels are present on the soma and dendrites. SK channels are also present in the spine heads (Ballesteros-Merino et al., 2012), and there is an approximate number of how many channels are in one spine (2–3 channels/spine). The number of SK channels in dendritic membrane patches and how this number is changing in the different dendritic regions was also available. It was a linear change, so we implemented that in the model, and we left the somatic value (from where it will linearly increase) as a free parameter between boundaries close to the experimental data (Ballesteros-Merino et al., 2012). Since this is a calcium-activated potassium channel and has no voltage dependence, the equations are different from the other potassium channels that are voltage dependent (Equations 49–52). The equations came from the channel model of Hirschberg et al. (1998). This channel model also had a built-in temperature dependence that we decided to use because the experimental data that it was fitted to was measured at room temperature (23°C) while our experimental data was measured at body temperature (33°C).


$$I_{k}=(\text{gmax}\times \mathrm{rat}e_{k})\times w\times (V-e_{k})$$ (49)

$$\mathrm{rat}e_{k}=1.5^{(\text{celsius}-23)/10}$$ (50)

$$w_{\infty }=0.81/(1+e^{\frac{\log(\text{wcai})+3.3}{-0.46}})$$ (51)

$$\tau_{w}=\tau_{\text{sKCa}}/\mathrm{rat}e_{k}$$ (52)

where $\tau_{\text{sKCa}}$ = 23.65 ms and wcai is the calcium concentration.

The $\mathrm{gma}x_{\text{sKCa}\_\text{soma}}$ was a fitted parameter. We fitted an equation to get the maximal conductance ratios equal to the SK channel densities in different dendritic regions stated in Ballesteros-Merino et al. (2012). From this the maximal conductance as a function of distance from the soma is given by Equation 53:


$$\mathrm{gma}x_{\text{sKCa}}=\mathrm{gma}x_{\text{sKCa}\_\text{soma}}\times (1+0.00002\times \mathrm{dis}t_{\text{soma}})$$ (53)

###### Slow Afterhyperpolarization potassium (K-AHP) current

The potassium current responsible for the slow afterhyperpolarization that occurs in the CA1 pyramidal cells was initially assumed to be generated by the SK-channels, but this later proved to be false. There were also reports about intermediate-conductance calcium-dependent potassium channels that are present in other cell types, may also exist in CA1 pyramidal cells, and may be responsible for the slow afterhyperpolarization (King et al., 2015). Since there was a debate about these findings and there is no available data about the kinetics of this channel, we kept a phenomenological calcium-dependent potassium channel model that produces the desired behavior at the soma. It was described with first-order kinetics modeled using transition rates, similar to the original Hodgkin-Huxley equations (Equations 10, 11), where α = 0.2 × calcium concentration if the concentration was less than 50 mM, and α = 10 if the concentration was above 50 mM, and *β* = 1. The maximal conductance (gmax) was a fitted parameter. Since the channel that is responsible for slow afterhyperpolarization is co-localized with the L-type calcium channels (Marrion and Tavalin, 1998), our channel model gets the calcium ions solely from the L-type calcium channels. The kinetics were based on the observations of Traub et al. (1991).

##### Calcium channels

###### L-type calcium channel

High-voltage-activated L-type calcium channels play a major role in shaping the currents in the dendrites and spines of the CA1 pyramidal cell. Although both calcium-dependent and voltage-gated inactivation exist in these channels, they were neglected because of their slow time course.

The steady-state activation is based on Bell et al. (2001), and the current is described by the GHK equation (Equations 15–19). Specifically, for the L-type calcium channel, the channel kinetics are described by Equations 54–56:


$$I_{\mathrm{CaL}}={\bar{p}}_{\mathrm{CaL}}\times X^{2}\times \mathrm{ghk}(V,{\left[Ca^{2+}\right]}_{\text{inside}},{\left[Ca^{2+}\right]}_{\text{outside}})$$ (54)

The rates are the following:


$$\alpha =A_{\alpha }\times e^{(V-\text{Vhal}f_{\alpha })/B_{\alpha }}$$ (55)

$$\beta =A_{\beta }\times e^{(V-\text{Vhal}f_{\beta })/B_{\beta }}$$ (56)

where $A_{\alpha }$ = 0.3, $B_{\alpha }$ = 17 and $\text{Vhal}f_{\alpha }$ = 6.5, while $A_{\beta }$ = 0.3, $B_{\beta }$ = − 17 and $\text{Vhal}f_{\beta }$ = 6.5.

The permeability of the channel was a fitted parameter. According to Magee et al. (1998) and Marrion and Tavalin (1998), L-type calcium current is present perisomatically, then decreases further from the soma; therefore, we applied an inhomogeneous distribution of this ion channel according to Equations 57, 58:


$${\bar{p}}_{\mathrm{CaL}}=\text{ratio}\times (0.002+(-1.3333\times 10^{-5}\times \mathrm{dis}t_{\text{soma}})\times 0.2$$ (57)

up to 150 μm distance and from there


$${\bar{p}}_{\mathrm{CaL}}=\text{ratio}\times (0.002+(-1.3333*10^{-5}\times 150))\times 0.2$$ (58)

The fitted parameter was the ratio.

###### N-type calcium channel

The model contains N-type calcium channels exclusively on the soma. Channel gating is described by the classical Borg-Graham formalism, and the current is calculated via the GHK equation (Equations 15–19, 59).


$$I_{\mathrm{CaN}}={\bar{p}}_{\mathrm{CaN}}\times X^{2}\times Y\times \mathrm{GHK}$$ (59)

The parameters for the X and Y equations can be found in Table 2. The kinetics were based on McNaughton and Randall (1997).

###### T-type calcium channels

We decided to implement one T-type calcium channel that describes the current measured in a CA1 pyramidal cell (Randall and Tsien, 1997). The steady-state activation and inactivation curves and the time constant of the activation were described by the Borg-Graham formalism (Equations 12, 13), whose parameters can be found in Table 2, while the time constant of the inactivation was described as follows (Equation 60):


$$Y_{\tau }=1.6531\times e^{-0.0659\times V}+20$$ (60)

The number of different T-type calcium channel isoforms was explicitly measured by McKay et al. (2006) in every region of the dendritic arbor and the soma. Since the model has only one type of T-type calcium channel, we summed up the total numbers of each isoform present in the given region. We could not find a simple fitting equation to describe the gradient of the current, and therefore, we explicitly used the measured permeability in each region and introduced a single multiplier that scaled all permeability values as a fitted parameter.

The baseline permeability on the oblique dendrites was 7.2608 × 10^−7^, on the basal dendrites it was 6.9983 × 10^−7^, on the apical trunk 2.4292 × 10^−7^, on the tuft dendrites 4.3133 × 10^−7^, and on the soma, it was 2.125 × 10^−8^ cm/s.

###### R-type calcium channel

Cav2.3, which is the Ca channel subunit responsible for the R-type current in the CA1 pyramidal cells, is exclusively present on dendritic spines (Bloodgood and Sabatini, 2007). One spine head can contain 5–15 channels (Yasuda et al., 2003); based on this and the known single channel conductance, we calculated the feasible range of permeability values to set the boundaries for this optimized parameter. The properties of the channel were described by Foehring et al. (2000) and Randall and Tsien (1997). We used the GHK equation (Equations 15–19) to describe the channel behavior, and its parameters can be found in Table 2.

In those simulations where dendritic spines were not explicitly modeled, R-type Ca channels were included in models of the corresponding dendritic segments, and their density was set using the surface factor (F-factor) – however, since these channels were assumed to be present only in the spines, the multiplying factor for the maximal permeability was F-factor minus one (rather than F-factor, which would imply an equal density of the channel in the membranes of spines and the dendritic shaft).

###### NMDA receptor

To make the model simultaneously perform well on several HippoUnit tests and qualitatively match the experimental data of Losonczy and Magge (2006) we created a new NMDA receptor model. (See Results for more explanation).

The voltage dependence of the Mg-block was fitted with a sigmoid function (Equation 61):


$$\text{Mgblock}=1/(1+e^{-(V-\text{Vhalf})/k})$$ (61)

This equation can describe the voltage dependence of many previous NMDA receptor models as well, and its parameters have clear interpretations as the midpoint and (inverse) steepness of the sigmoid. The final parameters are Vhalf = − 6 mV and k = 18 mV.

The equations describing the behavior of the receptor model are Equations 62, 63:


g=(B−A)×Mgblock(V) (62)

I=g×(V−e)×(1−pf) (63)

Here, A and B are state variables that describe the control of presynaptically released glutamate over the opening and closing of the ion channel, A corresponds to the rise and B to the decay of the current, and B−A is analogous to X in the Hodgkin-Huxley equations (Equations 9–11), modified by the synaptic weight of the NMDA channels in the model. Following a presynaptic spike and subsequent transmitter release, A and B change instantaneously according to Equations 64–67:


A=A+weight×factor (64)

B=B+weight×factor (65)

$$\text{factor}=1/(-e^{-\frac{\mathrm{tp}}{\tau_{1}}}+e^{-\frac{\mathrm{tp}}{\tau_{2}}})$$ (66)

$$\mathrm{tp}=\frac{\tau_{1}+\tau_{2}}{\tau_{2}-\tau_{1}}\times \log\frac{\tau_{2}}{\tau_{1}}$$ (67)

The scaling factor is set such that a single presynaptic release event through a synapse of weight 1 generates a peak conductance of 1.

In the absence of presynaptic activity, A and B decay exponentially according to Equations 68, 69:


$$A^{\prime }=-A/\tau_{1}$$ (68)

$$B^{\prime }=-B/\tau_{2}$$ (69)

pf = 0.03, which is adjusted so that 15% of the whole current through the NMDA channel is mediated by calcium ions at −60 mV, $\tau_{1}$ is the rise time that equals 3.3 ms and $\tau_{2}\;$ is the decay time constant, which equals 102.38 ms. $\tau_{1}$ and $\tau_{2}$ are Q10-corrected values from McDermott et al. (2006).

##### Calcium dynamics

The model contains four different types of pools for intracellular calcium ions, each with its own separate dynamics. One general calcium pool is not assigned to any specific calcium channel and is used in the soma and the dendrites, another one is present in the spines, a third calcium pool is for the calcium ions that flow in through the N-type calcium channels and are then used by the BK potassium channels, and a final calcium pool is for the calcium ions that flow in through the L-type calcium channels. The latter is connected to the hypothetical K_AHP current, since the slow afterhyperpolarization is mediated by an only calcium dependent potassium channel that is most likely co-localized with the L-type potassium channels (Marrion and Tavalin, 1998).

The calcium dynamics models calcium accumulation into a volume of area×depth next to the membrane with a decay time constant (tau) to the resting level. The general calcium dynamics and the dynamics in the spine heads are described by Equations 70–71.


$$\mathrm{ca}i^{\prime }=\frac{\text{drive}}{1+\text{bcap}}+({\left[Ca^{2+}\right]}_{0}-[Ca^{2+}])/\tau$$ (70)

where ${\left[Ca^{2+}\right]}_{0}$ is the baseline calcium concentration (5 × 10^−5^ mM), τ = 1.33 ms, and the buffer capacity (bcap) = 17.


$$\text{drive}=I_{\mathrm{Ca}}/(2\times F\times \text{depth})$$ (71)

where F is the Faraday-constant, and depth is the shell where the calcium ions take effect, most of the time the half of the diameter of the dendrite, soma or spine head, while in the pools assigned to specific calcium channels the depth = 1 nm to model the co-localization of the calcium channels and the calcium-dependent potassium channels. If the drive <= 0 then drive = 0, so it cannot change the direction of the flow of the ions.

For the two channel-specific calcium pools bcap=0 .

##### Leak

The model also contains a non-specific “leak” current that represents all non-voltage-dependent ionic currents (Equation 72).


$$I_{\text{Leak}}=\mathrm{gma}x_{\text{Leak}}\times (V-e_{\text{Leak}})$$ (72)

where both the maximal conductance and the reversal potential were fitted parameters of the model.

### Tools

#### Neuron

Simulations were carried out using the NEURON simulation environment (version 8.2). The model was defined in NEURON’s HOC language, and channel models were implemented in the NMODL programming language (Carnevale and Hines, 2009).

#### Parameter fitting

We used automated parameter fitting methods to determine those model parameters that were not sufficiently constrained by the experimental literature. In a few cases where a published experimental value led to clearly incorrect model behavior, we allowed that parameter to be optimized within realistic boundaries. Table 3 contains the fitted parameters and their boundaries.

**Table 3:** Fitted parameters with their corresponding boundaries.

Fitted parameter	Minimum	Maximum
Maximal conductance of the leak current	0.0000005 S/cm^2^	0.00001 S/cm^2^
Reversal potential of the Leak current	−120 mV	−20 mV
The ratio of the inhomogeneous Ih current from a pre-established baseline	0.1	20
The half steady-state inactivation of the somatic sodium channels	−60 mV	−25 mV
The half steady-state activation of the somatic sodium channels	−40 mV	−20 mV
Maximal conductance of somatic sodium channels	0.02 S/cm^2^	0.5 S/cm^2^
Maximal conductance of dendritic delayed-rectifier potassium channels	0.01 S/cm^2^	0.2 S/cm^2^
Maximal conductance of somatic delayed-rectifier potassium channels	0.01 S/cm^2^	0.8 S/cm^2^
Maximal conductance of the D-type potassium channels	0.0001 S/cm^2^	0.01 S/cm^2^
Maximal conductance of axonal D-type potassium channels	0.000001 S/cm^2^	0.1 S/cm^2^
Maximal conductance of the M-type potassium channels on the soma and dendrites	0.0000001 S/cm^2^	0.0005 S/cm^2^
The ratio of the inhomogenous A-type potassium channels from a pre-established baseline	0.5	5.0
A shift in the steady-state activation and inactivation curves of the A-type potassium channels	0 mV	50 mV
The slope of the A-type potassium channel activation	5 mV	22 mV
Permeability of the N-type calcium channels	0.002 cm/s	0.01 cm/s
Maximal conductance of the BK-type potassium channels	0.001 S/cm^2^	0.3 S/cm^2^
The ratio of the inhomogeneous L-type calcium channels from a pre-established baseline	0.1	5.0
Maximal conductance of the slow afterhyperpolarizing current	0.00001 S/cm^2^	0.001 S/cm^2^
The ratio of the inhomogenous T-type calcium channels from a pre-established baseline	0.1	5.0
Maximal conductance of the SK-type potassium channels	0.00001 S/cm^2^	0.001 S/cm^2^
Permeability of the R-type calcium channels	0.00001 cm/s	0.001 cm/s

The parameter fitting procedure was carried out by the Neuroptimus software (Mohácsi et al., 2024) which is an interactive Python tool for the automated fitting of unknown parameters between given boundaries. The optimized error function was a sum of standardized feature errors based on the feature values extracted from the voltage traces by the eFEL feature extraction library. For the optimization algorithm, we chose the Covariance Matrix Adaptation Evolution Strategy from the CMAES package as it was found by Mohácsi et al. (2024) to be one of the best algorithms to fit the parameters of a morphologically and biophysically detailed neuronal model. We set the parameters of the search algorithm to get the best possible results within an acceptable runtime. The population size was 127, which was determined by the number of cores per node available on the Neuroscience Gateway’s supercomputers (Sivagnanam et al., n.d.) that we used for the optimization process. We ran the search for 100 generations, as the results of Mohácsi et al. (2024) indicated that this was sufficient for the CMAES algorithm to converge to a solution whose error score was close to the global minimum.

#### Model testing

To validate the models against experimental data that were not considered during the optimization process, we used the HippoUnit software (Sáray et al., 2021). HippoUnit is a Python module that automatically performs simulations that mimic experimental protocols on detailed hippocampal cell models. It uses models built in NEURON (Carnevale and Hines, 2009) as a user-defined Python class. We used all the five built-in tests:

The Somatic Features Test uses the eFEL Python package to extract and evaluate the values of both subthreshold and spiking features from the voltage responses of the model to somatic current injections of different depolarizing and hyperpolarizing current amplitudes. Spiking features describe action potential shape (such as AP width, AP rise/fall rate, AP amplitude, etc.) and timing (frequency, inter-spike intervals, time to first/ last spike, etc.), while passive features (such as the voltage base or the steady state voltage), and subthreshold features for negative current stimuli (voltage deflection, sag amplitude, etc.) are also examined. Step currents of varying amplitudes are injected into the soma of the model, and the voltage response is recorded. Its outputs are the features extracted from the models and error values resulting from comparisons to the features extracted from the experimental data (see earlier Methods, Data section), an error score that is the mean of the errors of all the features examined by the given test, and figures that illustrate the behavior of the model. This test computes features and errors that are similar to those used during parameter optimization, but it tests more features to determine how the model performs on non-optimized features.

The Backpropagating Action-Potential Test evaluates the strength of action potential backpropagation on the apical trunk at different distances (50, 150, 250, 350 μm) from the soma. The test finds the amplitude of the current injection where the model fires at exactly 15 Hz (or the closest possible rate) and then measures the amplitude of the first and last action potentials in the spike train (Golding et al., 2001). The final score here is the average of the Z-scores achieved for the features of first and last action potential amplitudes at different dendritic distances. In the result, it is also stated whether the model is more like a strongly or a weakly propagating cell (Golding et al., 2001). We modified the original test from Sáray et al. (2021), and instead of evaluating the absolute value of the amplitude of the last action potential, the ratio of the amplitudes of the first and last action potentials was used to address the fact that the shrinkage of the spikes is independent of the absolute value of the spike amplitudes.

The Post-synaptic Potential Attenuation Test tells us about the attenuation of postsynaptic potentials from input sites in different parts of the dendritic tree to the soma (Magee and Cook, 2000). The apical trunk receives excitatory post-synaptic current (EPSC)-shaped current stimuli at locations of different distances from the soma. The somatic and dendritic depolarization values were then averaged in three ranges around distances of 100, 200, and 300 μm from the soma, and the soma/dendrite attenuation was calculated to get the mean and standard deviation of the attenuation features at the three different input distances.

The Depolarization Block Test determines whether a model enters depolarization block in response to a prolonged and high-intensity somatic current stimulus and also determines the minimal current required to elicit this behavior. CA1 PCs respond to somatic current injections of increasing intensity with an increasing number of action potentials until a threshold is reached. For current intensities higher than the threshold, the cell does not fire over the whole period of the stimulus; instead, firing stops after some action potentials, and the membrane potential is sustained at some constant depolarized level for the rest of the stimulus. This phenomenon is known as depolarization block (Bianchi et al., 2012). The test attempts to determine the threshold current by stimulating the soma of the model with 1,000 ms long square current pulses of increasing amplitudes from 0 to 1.6 nA in 0.05 nA steps. The model is considered to exhibit depolarization block if the largest number of action potentials is not displayed at the highest current amplitude tested and if a higher current intensity exists where the model does not fire during the last 100 ms of its voltage response. Those models that do not enter depolarization block at all get an error score of 100.

The Oblique Integration Test evaluates the signal integration properties of radial oblique dendrites by providing an increasing number of synchronous or asynchronous clustered synaptic inputs that mimic glutamate uncaging experiments.

The test evaluates the voltage threshold for dendritic spike initiation defined as the expected somatic depolarization at which a step-like increase in peak dV/dt occurs; the proximal threshold defined the same way as above, but including only those results in the statistics where the proximal part of the examined dendrite was stimulated; distal threshold; degree of nonlinearity at threshold; suprathreshold degree of nonlinearity; peak derivative of somatic voltage at threshold; peak amplitude of somatic EPSP; time to peak of somatic EPSP; degree of nonlinearity in the case of asynchronous inputs (Losonczy and Magge, 2006).

The test automatically selects a list of oblique dendrites that meet the criteria of being a terminal branch not further than 120 μm from the soma, then an increasing number of synaptic inputs are activated at the selected dendritic locations separately, while recording the local and somatic voltage response. To make the test mimic the glutamate uncaging experiments better, we put a single dendritic spine at the chosen dendritic location, which receives the synaptic input.

The synaptic weights for each selected dendritic location are automatically adjusted by the test using a binary search algorithm so that the threshold for dendritic spike generation is 5 synchronous inputs. Those dendritic locations where this first dendritic spike generates a somatic action potential, or where no dendritic spike can be evoked, are excluded from further analysis. We also modified the criteria for choosing dendritic location and weight - in the original test, the model should not fire any action potential for 5 inputs. We added that it should not fire at 6 inputs either, because somatic action potentials at 6 inputs interfere with the results and their interpretations. To reduce the probability of firing too early, we also gave a small (− 0.1 nA) constant hyperpolarizing current injection to the soma.

In this test, we did not use the built-in NMDA receptor model of HippoUnit; instead, we tried several different NMDA receptor models found in the literature. To determine the right AMPA/NMDA ratio for each receptor model, we implemented the results of Otmakhova et al. (2002). We applied somatic voltage clamp at −65 mV, then recorded the voltage response of the cell when we gave no input to the cell, when we stimulated the cell with one single synaptic input on a proximal dendrite containing only AMPA current, and then when stimulated with a synaptic input with both AMPA and NMDA current in it. We subtracted the no-input trace from the other two cases as a correction, then took the integral of the corrected traces and calculated their ratio as follows:


$$\text{area ratio}=\frac{\begin{array}{l} \text{AMPAandNMDA response integral}-\\ \;\;\;\;\;\;\;\;\;\;\;\;\text{AMPA response integral} \end{array}}{\text{AMPA response integral}}$$

According to the article, this should result in a 3.7 ± 0.8 ratio in the Schaffer-collaterals. We used an AMPA/NMDA ratio for each NMDA receptor model that gives an area ratio within one standard deviation of the experimental result. To find the appropriate conductance ratio for a particular NMDA receptor model, we started from an AMPA/NMDA ratio of 0.5, and then if the area ratio was lower than 3.7 ± 0.8, we subtracted 0.1, and if it was higher, we added 0.1 until the area ratio got within 1 standard deviation of the experimental result. The model from Grunditz et al. (2008) could not be activated by one synaptic input sufficiently to get within the targeted range for the area ratio, and therefore, for this receptor model, we used the default 0.5 AMPA/NMDA ratio. Lastly, we created a new custom NMDA receptor model (see Methods above and Results) that fits our cell models best. The used AMPA/NMDA ratios with the corresponding area ratios can be seen in Table 4.

**Table 4:** The used AMPA/NMDA ratios for each NMDA receptor model with the corresponding area ratios.

Receptor model	AMPA/NMDA ratio	Area ratio
Jahr-Stevens	1	3.06
Magee	1.5	3.293
Major	0.4	3.475
Gao	0.4	3.116
Otmakhova	0.3	3.527
Larkum	0.25	3.436
Ujfalussy	0.5	3.874
Tar (custom NMDA receptor)	0.5	3.73

This version of HippoUnit, with the modifications made especially for this project and for validating models with dendritic spines, can be found at https://github.com/lucatar/hippounit/tree/hippounit_spine_project. Jupyter notebooks, target features, and the model can be found at https://github.com/lucatar/hippounit_CA1_demo.

## Results

### Overview of the model-building process

The primary goal of this study was to develop a detailed model of rat CA1 pyramidal neurons that properly integrates the current evidence regarding the relevant biophysical mechanisms and successfully captures the electrophysiological behavior of this cell type in a wide range of experimental conditions. To this end, we first systematically processed the available literature on CA1 pyramidal cells (PCs), with particular attention to the biophysical properties and spatial distributions of voltage-gated ion channels (see Methods). This allowed us to identify those parameters (mainly the absolute densities of the various channels in specific cellular compartments) that are not sufficiently constrained by the available data but are expected to have a significant effect on the electrical behavior of the neuron. We then used automated parameter search methods (Mohácsi et al., 2024) to set these parameters in a morphologically and biophysically detailed model neuron such that the electrophysiological behavior of the model best matched a set of experimental recordings under the same conditions. Finally, we validated the resulting model by quantitatively comparing its behavior to that of real CA1 PCs under a variety of different conditions using data that had not been used during parameter optimization. Figure 1A shows the model-building process.

Models were fitted to electrophysiological features extracted from somatic current-clamp recordings from rat CA1 pyramidal neurons (data courtesy of Judit Makara) using the eFEL feature extraction library (https://github.com/BlueBrain/eFEL). Only a subset of experimental features was used for optimization (see Table 1), and the rest (each eFEL feature available) were used only for validation. During model validation, the behavior of the model was also tested against published experimental data in several additional paradigms implemented by the HippoUnit test suite. These additional test results were not used during the automated optimization of model parameters, but they informed our decisions about subsequent refinements of basic modeling assumptions (such as the inclusion of specific channels or setting plausible parameter ranges).

In every iteration of model fitting, we ran 20 optimizations simultaneously from different, random starting points to avoid solutions where the parameter search process gets stuck in a local minimum instead of reaching the global best solution. Multiple runs also helped us identify when a parameter is consistently tuned near one of its boundaries, which indicated that a better solution could be achieved by extending the relevant boundary. In these cases, the parameter search was re-run with revised boundaries.

During parameter optimization, we used the original reconstructed morphology that did not include spines, and modeled the effects of spines by merging all the relevant biophysical mechanisms (such as the membrane capacitance and various conductances) with the ones present in the dendritic shaft (for details, see Methods and the section” The effect of explicitly modelling dendritic spines on the behavior of the model” below). The dendritic conductance parameters fitted during optimization correspond to their hypothetical values in the equivalent fully detailed model (with spines), but the actual values in the optimized model were calculated by also considering the density of the channel in the spine and the shaft, as well as the F-factor, and simulations were run using this model. Therefore, the maximal conductance parameters seen in Figure 2 are the uncorrected (hypothetical) values, and the maximal conductance values in the simulated spineless model can be different when a particular conductance is present both in the dendritic spines and the dendritic shaft.

**Figure 2:**
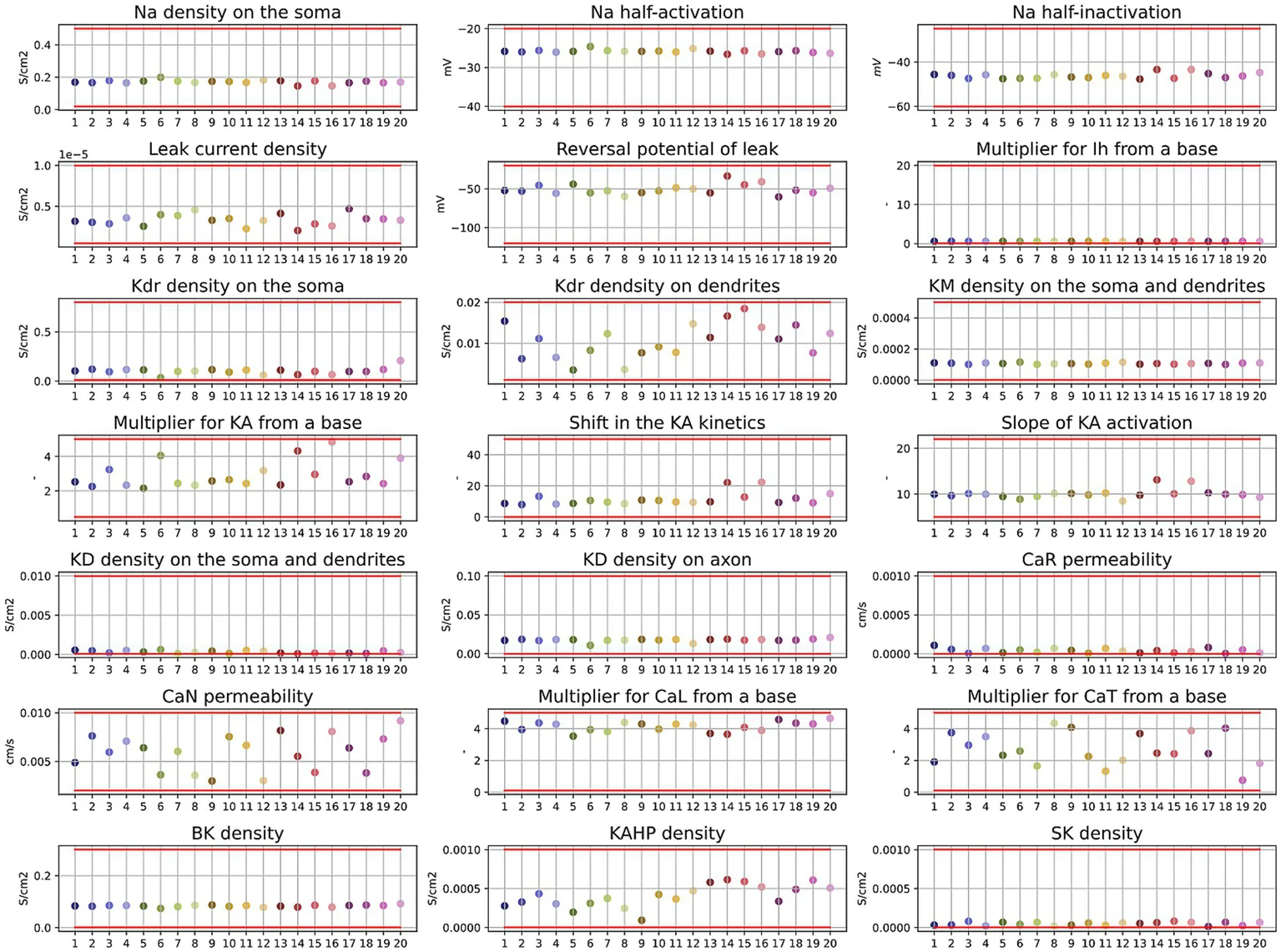
The fitted parameters of the 20 optimized models. The red lines are the upper and lower bounds for each parameter. The x-axis and the different colors represent the individual models by their numbers, while on the y-axis, there are the parameter values for each model and parameter. Supplementary Figure 1 shows versions of the plots in the panels corresponding to the “Multiplier Ih from base”, “Kdr density on the soma”, “KD density on the soma and dendrites”, “CaR permeability”, and “SK density” parameters using a different scale for the y-axis to allow a better view of the actual parameter values.

To validate the results, we used the HippoUnit software tool, which was developed specifically to compare various electrophysiological properties of detailed models of CA1 PCs with the corresponding experimental data (Sáray et al., 2021). HippoUnit has five built-in tests. The Somatic Features Test examines the somatic responses of the model cell to step current injections and compares these to experimental results based on the features of the eFEL library; the Oblique Integration Test examines signal integration in oblique dendrites based on the experimental protocol and results of Losonczy and Magee (Losonczy and Magge, 2006); the Post-synaptic Potential (PSP) Attenuation Test evaluates changes in the amplitude of post-synaptic potentials as they propagate toward the soma (Magee and Cook, 2000); the Back-propagating Action Potential (bAP) Test examines the properties of the action potentials that propagate back from the soma into the dendrites (Golding et al., 2001); and the Depolarization Block Test, based on Bianchi et al. (2012), tests whether the neuron goes into depolarization block in response to a sufficiently large somatic current injection and, if so, compares the characteristics of depolarization block with the relevant experimental results.

For candidate models from the parameter optimization phase, we first ran the Somatic Features Test. The outputs of this test include the feature values and feature errors extracted from the models, and the total error score, which is the average of the individual feature errors. We found that all the errors were reasonably small (the vast majority of the feature errors were within 2 standard deviations of the experimental mean), so we moved to other tests. We then evaluated the model from the perspective of the PSP Test and the Depolarization Block Test, then the bAP Test, and then moved to the Oblique Integration Test. Detailed results from these tests are presented below.

The final iteration of parameter search resulted in a set of 20 optimized models. In this final set of models, none of the parameters was consistently tuned close to one of its bounds (Figure 2; see also Supplementary Figure 2 for a closer view of five parameters whose tuned values were much smaller than the upper bound). The algorithm found very similar results in the case of parameters that play a role in shaping the somatic responses of the cell, while in the case of parameters mostly responsible for dendritic behavior, we found larger variability since we did not optimize for dendritic features (Figure 2). We also computed the pairwise correlations of the optimized parameters across the tuned models (Supplementary Figure 3). The majority of the correlation coefficients had moderate magnitude, but there were also several strong (positive or negative) correlations. Many of these strong correlations were between parameters that belonged to the same conductance (such as Na or KA), but there were also some strong correlations between parameters of different channels.

The models, the full optimization, and all the validation results, along with the scripts for further evaluation, can be found at: https://zenodo.org/records/21907403 and are also available on ModelDB: https://modeldb.science/2031080.

The following sections describe in detail the properties and behavior of these final models, including the construction of the set of ion channel models and synaptic receptor models, the results of a large set of validations, and a comparison of models in which dendritic spines were modeled at varying levels of detail.

### Models of voltage-gated ion channels

The properties, total quantities, and spatial distributions of voltage-gated ion channels are known to be critical for the construction of biologically faithful compartmental models of neurons. Therefore, we carefully evaluated the relevant experimental evidence regarding the voltage-gated ion channels of CA1 PCs and screened the modeling literature for potentially suitable existing channel models. All the candidate channel models were carefully re-evaluated and compared to the latest available data in the literature. The existing channel parameters were adjusted where needed, and if the exact value for a parameter was not available in the literature, it was either fitted by Neuroptimus or, when some kind of relationship was known, was bound to another optimized parameter. This new set of ion channel models greatly improved the model’s ability to reproduce a wide variety of experimental results, since their kinetics were often the source of deviations from the expected behavior. We used experimental data from the literature that were preferably from adult rat CA1 pyramidal cells. If data from rats were not available in sufficient detail, then data from mice were used, and in the case of L- and N-type calcium channels we used data from humans. If there was no sufficient data from CA1 pyramidal cells, then we turned to measurements from other cell types, and verified that the channel model has the same effect on the behavior of the model cell as the experimentally observed effect of the channel in CA1 pyramidal cells. We avoided channel data from gene expression systems since post-translational modifications and interactions with auxiliary proteins in nerve cells can significantly alter the channel kinetics. It was also an important factor that the experiments were carried out at body temperature, or the results were appropriately corrected for temperature.

The final version of the model contains the following currents (see Methods for more details).

- Especially in the subthreshold voltage range, the responses of the neuron are strongly influenced by the voltage-independent leak conductance. Since the observed leak conductance is likely the result of multiple ion channels with approximately linear current–voltage relationships but different ion selectivity, we chose to include both the reversal potential and the density (which was assumed to be uniform) of the leak channel among the unknown (fitted) parameters.
- The hyperpolarization-activated current (Ih) mediated by HCN channels also primarily shapes the subthreshold behavior of CA1 PCs. We used one Ih kinetics for the soma and the proximal dendritic arbor and a modified kinetics for the distal dendritic arbor (Zemankovics et al., 2010). The amount of Ih increases in the dendrites as a function of distance from the soma (Magee, 1998; Lörincz et al., 2002), so we fixed the proportion of Ih at different distances and fitted only a single scaling factor.
- The model has 3 types of sodium channels with different kinetics for the soma, axon, and dendrites to address the different contributions of Nav1.6 and Nav1.2 channels in the different regions of the cells (Lőrincz and Nusser, 2010). The somatic sodium channel density was optimized, while the axonal density was fixed to 40 times the somatic density. On the oblique and basal dendrites, it was 2/3 of the somatic density, and on the tuft dendrites, it was 1/3 of the somatic density based on the data of Lőrincz and Nusser (2010). On the apical trunk, the density decreased from the somatic value with increasing distance from the soma.

In regard to potassium channels, the model contains delayed-rectifier, D-type, M-type, and A-type voltage-gated potassium channels as well as several calcium-dependent (and, in some cases, also voltage-dependent) potassium channels.

- The delayed-rectifier potassium channel (Kdr) is a well-studied channel (Coetzee et al., 1999; Misonou et al., 2005; Rudya and McBain, 2001). Its main role is to rapidly repolarize the action potential and enable repetitive firing. Delayed rectifier potassium channels are present in every region of the CA1 PCs. We optimized the maximal conductance of the Kdr channels separately on the soma and the dendrites, while on the axon, the amount of Kdr was 40 times higher than the somatic value to match the increased maximal conductance of the sodium channels.
- The D-type potassium current is present in the soma, dendrites (Metz et al., 2007), and the axon, and its kinetics were carefully examined and adjusted to the literature (Storm, 1990; Storm, 1988), to get the delay at the beginning of the current step protocol to the first action potential, which can be observed in the experimental data (Storm, 1990). Since D-type potassium channels are heteromers regarding their α-subunit, which, as homomers, act like delayed-rectifier potassium channels, we found it logical to include D-type potassium channels in the axon and the soma as well, but their maximal conductance was separately fitted as parameters due to the lack of information (Coetzee et al., 1999).
- M-type potassium channels are present on the soma, axon, and dendrites. The kinetics came from Hönigsperger et al. (2015) (see Methods for details). The maximal conductance was the same for the soma and dendrites, while on the axon, the maximal conductance was 4 times the somatodendritic value (Shah et al., 2011).
- A-type potassium channels are present in the soma and the dendrites, and their maximal conductance increases with the distance from the soma (Hoffman et al., 1997). We fitted a multiplier parameter that changes the amount of A-type potassium current proportionally on the whole dendritic arbor from a starting point that is based on Kerti et al. (2011). We also introduced a shift as a fitted parameter that could modify the voltage dependence of the activation during our optimization process. It is known that A-type potassium channels can undergo different kinds of modulations that alter the channel kinetics (Varga et al., 2004; Lindroos et al., 2018; Siwiec et al., 2020; Jerng and Pfaffinger, 2014; Kise et al., 2021; Murphy et al., 2022), which gives us some freedom to modify the kinetics to better fit the model’s needs.
- The model contains N-type calcium channels exclusively on the soma. The modeling of the N-type calcium channel follows the Goldman-Hodgkin-Katz equation, with its permeability being a fitted parameter. Its kinetics are based on McNaughton and Randall (1997).
- High-voltage-activated L-type calcium channels play a role in shaping the currents in the dendrites and spines of the CA1 pyramidal cell. It is also important to note that calcium ions entering through the L-type calcium channels are responsible for activating the calcium-dependent potassium channel that shapes the slow afterhyperpolarization (Bowden et al., 2001). L-type calcium channels are present on the soma and dendrites, but the amount of the channels is linearly decreasing with the distance from the soma (Marrion and Tavalin, 1998; Magee et al., 1998). The channel kinetics are based on Bell et al. (2001).
- The T-type calcium channels kinetics were based on Randall and Tsien (1997) while the approximate distribution in each dendritic region came from McKay et al. (2006). Since the densities measured by McKay et al. did not seem to follow any simple rule, the relative amount of T-type calcium channels in the model was set directly according to their data (see Methods). Since McKay et al. measured the three different subtypes of the T-type calcium channels separately, in this study, the summed amount was used for our channel model as a baseline amount, and then we used a multiplier as a fitted parameter so the ratios of the maximal conductances between the dendritic regions were preserved.
- R-type calcium channels have a major role in shaping the EPSP and EPSC in the spine heads during synaptic stimulation (Bloodgood and Sabatini, 2007). One spine head can contain 5–15 channels (Yasuda et al., 2003); based on this and the known single-channel conductance of Cav2.3 (the subunit responsible for the R-type current in the CA1 pyramidal cells), we could calculate the feasible range of permeability values to set the boundaries for this optimized parameter. The properties of the channel were described by Foehring et al. (2000) and Randall and Tsien (1997). Since the optimized models do not have explicitly modeled spines, which are instead taken into account by the increased membrane area of the dendrites, in these models, the R-type calcium channels are effectively present on the dendritic shaft, which may artificially increase their role in shaping the cell’s response to different inputs. We will examine the effects of modeling spines at different levels of detail in a later section.
- Big-conductance (BK)- type calcium and voltage-dependent potassium channels read out the calcium concentration exclusively from the N-type calcium channels, since according to the literature, these two kinds of channels are only a few nanometers apart from each other in the membrane (Marrion and Tavalin, 1998). The BK-type potassium channel has a role in shaping the fast afterhyperpolarization and spike repolarization (Gu et al., 2007). The BK-type potassium channel that we used originates from Maurice et al. (2004). It has both voltage and calcium-dependent activation modeled as two separate gates since we have no information whether the voltage influences the calcium dependency of the activation.
- We also introduced the Small-conductance (SK) calcium-dependent potassium channel to the model. It is present in the soma, the dendrites, and spine heads (2–3 channels/spine). According to Ballesteros-Merino et al. (2012) the number of SK-channels increases linearly with the distance from the soma. The somatic value was a free parameter between boundaries close to the experimental data (Ballesteros-Merino et al., 2012), and from that, the number of SK-channels increases with distance from the soma. The channel kinetics were based on Hirschberg et al. (1998).
- Connected to the L-type calcium channels, we introduced a generic calcium-dependent potassium channel to the model that is responsible for slow afterhyperpolarization, since there is contradictory information about the identity of this channel (King et al., 2015). Its kinetics were based on Traub et al. (1991), it was only present on the soma, and the maximal conductance of the channel was a fitted parameter.

Dendritic spines are specialized compartments of neurons, both in a biophysical and biochemical sense. They play critical roles in the generation of excitatory postsynaptic potentials and in synaptic plasticity. Therefore, we paid special attention to identifying the biophysical mechanisms (voltage-gated and ligand-gated ion channels) that shape electrical responses in dendritic spines. Based on the literature, we included the following voltage-gated channels in the dendritic spines of our model neurons.

- A-type potassium channels, with their maximal conductance density matching that of the parent dendrite from which the spine originated. Depending on the location of the spine, either proximal or distal A-type potassium channels were used.
- Small-conductance (SK-type) calcium-activated potassium channels, whose maximal conductance density also matched that of the parent dendrite.
- L-type calcium channels, whose density (permeability) also came directly from the originating dendritic location.
- R-type calcium channels, whose permeability was determined by the parameter fitting process.
- T-type calcium channels, whose permeability was provided directly by the experimental data (McKay et al., 2006) and depended on the dendritic region where the spine was located, except for the trunk, where we assumed in the absence of more accurate data that the permeability of T-type calcium channels in spines equals the permeability at the same location on the shaft. The permeability on the dendritic shaft was a fitted parameter.

### Model validation

In this section we describe the behavior of the optimized models mainly through the application of the HippoUnit tool, which was developed specifically to enable the comprehensive, quantitative validation of detailed models of CA1 PCs (Sáray et al., 2021). However, there are a few basic properties of these neurons that are not currently included in the HippoUnit framework (and that were not directly optimized for during the parameter search), which are nevertheless important indicators of the physiological behavior of CA1 PCs. These properties, which were evaluated separately before the application of HippoUnit, were the following:

The cells should not fire spontaneously before current injections, and the model should stop firing when the current injection ends. It was also a requirement for the spikes to initiate in the axon (Supplementary Figure 4). Finally, it was important that our model should not enter depolarization block in response to the optimized current injections.

Supplementary Table 1 contains the final scores of the models on each HippoUnit test. All 20 optimized models gave very similar results, which suggests convergence to similar low-error solutions from different random initializations.

#### Somatic features test

We first evaluated the models on the somatic features test. The resulting traces and the wider variety of features allowed us to get a more in-depth understanding of the models. In Figure 3, there is a selection of representative and diverse features of the models, and the models’ results compared to the experimental data. Note that this is only a selection of our choice for demonstrative purposes. The entire set of validation results can be found at: https://zenodo.org/records/21907403. A typical model’s voltage response to the somatic current injection protocol used in this test can be seen in Supplementary Figure 5.

**Figure 3:**
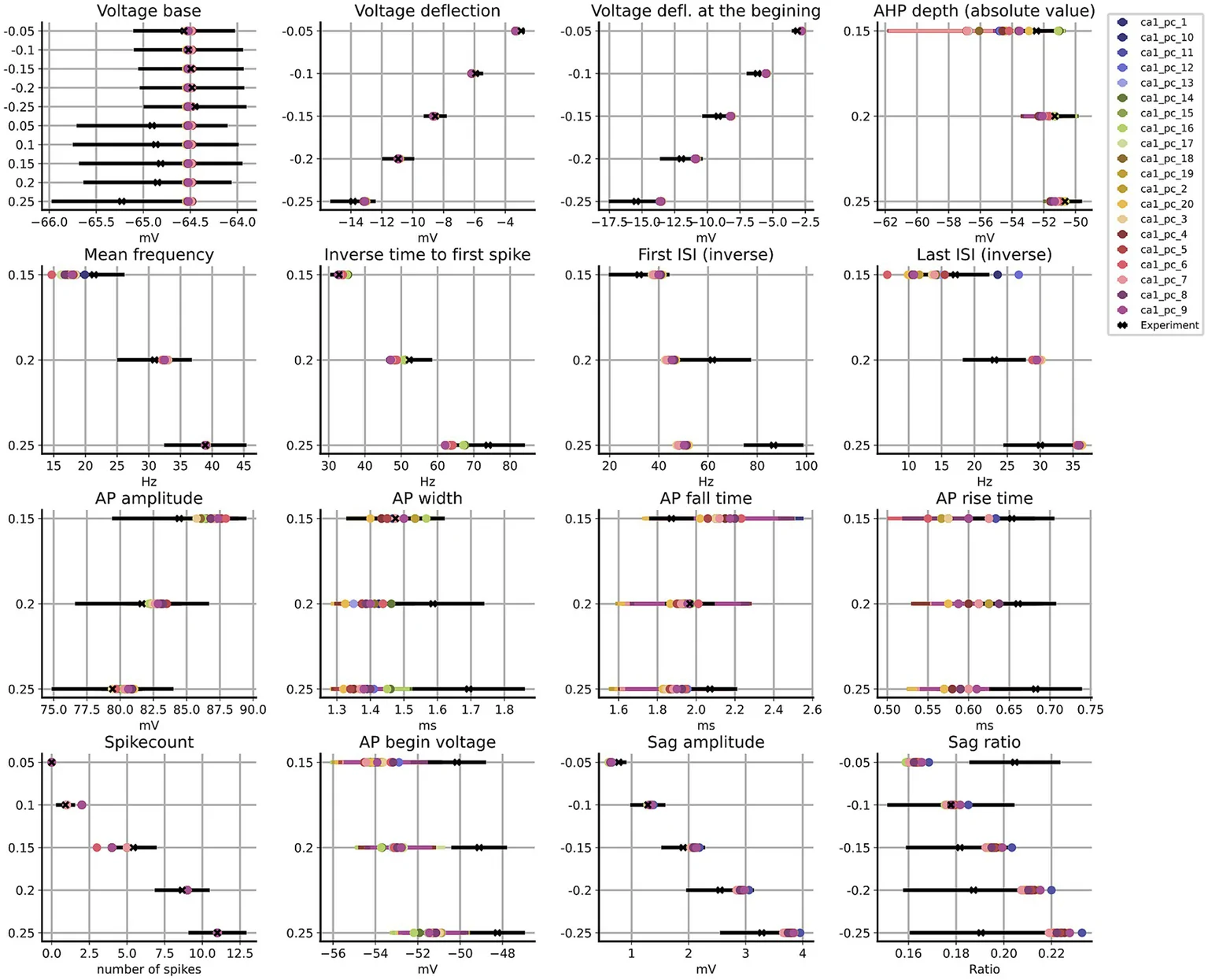
The result of the somatic features test of HippoUnit for the 20 optimized models. The figure shows 16 selected features that represent different aspects of the somatic behavior of the cells, from passive properties to action potential shapes and spiking patterns. Each panel shows feature values on the x axis for several different amplitudes of the somatic current injection, indicated on the y axis. The mean of the experimental feature value is indicated by the black cross, the associated experimental standard deviation by the black bar, and the feature values extracted from the responses of different optimized models are represented by the colored dots.

#### Backpropagating action potential test

Getting backpropagating action potentials right was crucial for the further examination of the dendritic integration of the models. The amplitude of the backpropagating action potential depended on the amount of sodium channels, and the difference between the first and the last action potential amplitude depended on the inactivation of the sodium channels on the dendrites (see Supplementary Figure 6). All the models were “strong-propagating” cells according to the experimental classification (Golding et al., 2001). Identifying the necessary conditions for weak propagation was outside of the scope of this study, and this issue was previously investigated by Bernard and Johnston (2003) and Golding et al. (2001). Results of the Backpropagating action potential test are in Figures 4A,B.

**Figure 4:**
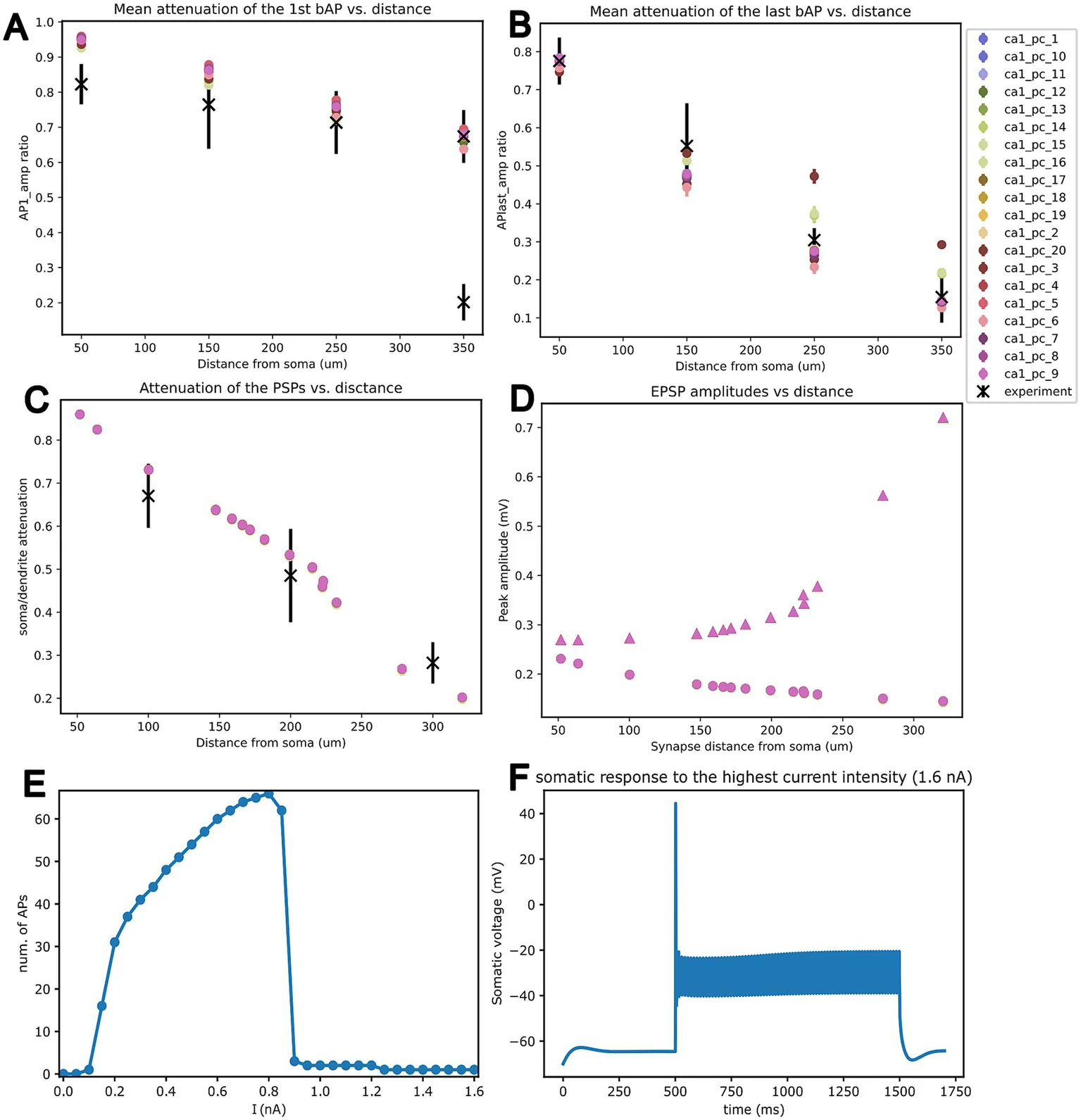
Validation results of the 20 optimized models. **(A,B)** Results from the Back-propagating Action Potential Test of HippoUnit. **(A)** shows the amplitude of the first action potential propagating along the apical trunk from the soma toward the distal dendrites, relative to the AP amplitude at the soma. **(B)** shows the same amplitude ratio for the last back-propagating action potential in a one-second train of APs (at approximately 15 Hz). The two experimental targets for the attenuation of the first AP correspond to the strongly and weakly propagating cell populations in the study of Golding et al. Most of the models behaved very similarly, and all of them were similar to strongly propagating CA1 PCs. **(C,D)** Results of the Post-synaptic Potential Attenuation Test, where an EPSP evoked in the apical dendrites propagates toward the soma. **(D)** shows the amplitude of the EPSP at the location of the synapse (triangles) and at the soma (dots), and **(C)** shows the ratio of these two amplitudes. The models showed very little variability. **(E,F)** An example of the results from the Depolarization Block Test. **(E)** shows the f-I curve of a model, which could be interpreted as a depolarization block, but the actual trace in **(F)** shows that there are spikes throughout the current step even for the largest injected current, but they become smaller than the detection threshold of −20 mV.

#### Post-synaptic potential attenuation test

We found that the attenuation of post-synaptic potentials depended mainly on the passive properties of the cell model. Based on the recent *in vivo* findings about a stronger coupling between the soma and the dendrites (Francioni and Harnett, 2022) and computational observations made in our lab (Terbe et al., 2022), we lowered the axial resistance of the model from 100 to 50 Ohm×cm, which resulted in a good fit to the experimental data (Figures 4C,D). Using the traditional 100 Ohm×cm axial resistance resulted in too strong attenuation (see Supplementary Figure 7), which remained the same after re-optimizing the model, and was therefore independent of the fitted parameters.

#### Depolarization-block test

The Depolarization Block Test examines whether the soma enters a depolarization block and how much injected current is needed for that. According to the test, the performance of the models varied: there were some that entered depolarization block, but others did not. However, looking at the individual traces, we concluded that, in fact, all the models showed qualitatively similar behavior: in response to large current injections, the amplitude of somatic APs was reduced drastically, but small spikelets always remained. The result of the test depended on whether these spikelets reached the −20 mV detection threshold. If they did, the test reported that the model did not enter depolarization block, and when they did not, it was considered a depolarization block. An example of this phenomenon can be seen in Figure 4E, which shows the f-I curve of the model, and Figure 4, which shows the corresponding voltage trace. The spikelets themselves originate from the axon, then backpropagate to the soma (Supplementary Figure 8). We conclude that the soma could enter a real depolarization block in principle, but the axon currently prevents this. Despite our model failing this HippoUnit test, there is experimental evidence that supports the presence of spikelets coming from the axon (Apostolides et al., 2016). A more detailed and fine-tuned axon could probably help to capture this phenomenon more accurately.

#### Finding the correct voltage dependence of the NMDA receptor model using the oblique integration test

Cortical pyramidal neurons, including CA1 PCs, implement highly nonlinear forms of dendritic integration, which are mediated by several distinct mechanisms such as Na spikes, Ca spikes, and NMDA spikes or plateaus (Spruston, 2008). More specifically, the thin apical oblique dendrites of CA1 PCs show supralinear summation of synaptic inputs arriving at nearby dendritic spines if these inputs are activated within a short time window, and the phenomenon depends on the activity of both dendritic voltage-gated Na channels and NMDA receptors (Losonczy and Magge, 2006). The Oblique Integration Test of HippoUnit replicates some of the key experiments in Losonczy and Magge (2006) to compare the characteristics of synaptic integration in oblique dendrites of detailed CA1 PC models to the corresponding experimental data (Sáray et al., 2021).

Unlike the previously presented validations that did not utilize synapses or (in the case of the PSP Attenuation Test) used only a standardized AMPA-type synaptic current input, the results of the Oblique Integration Test are strongly influenced by the properties of synaptic inputs, and it was therefore crucial to have a realistic model of excitatory synapses that are activated by the Schaffer Collaterals in this dendritic region. These inputs are known to activate both AMPA- and NMDA-type glutamate receptors, so it was important to properly capture the conductance ratio of the two receptors as well as their kinetics, and especially the voltage dependence of NMDA receptor activation, which is a critical factor that determines the emergence and properties of NMDA spikes/plateaus.

By default, HippoUnit uses the NMDA receptor voltage dependence from the model of Jahr and Stevens (1990) and an AMPA/NMDA conductance ratio of 2.0 in the Oblique Integration Test. However, this configuration produced sublinear dendritic integration and a correspondingly high error in the test. On the other hand, the experimental and modeling literature contains several competing models of NMDA receptors with distinct voltage dependence, and there are also relevant experimental results that constrain the AMPA/NMDA ratio in Schaffer collateral synapses. Therefore, we systematically evaluated oblique dendritic integration with several different models of NMDA receptors and recalculated the AMPA/NMDA conductance ratio based on the available experimental evidence. We determined the AMPA/NMDA conductance ratio separately for each NMDA receptor model based on the experimental study of Otmakhova et al. (2002). These authors used somatic voltage clamp and pharmacological blockade of NMDA receptors to measure the contributions of AMPA and NMDA receptors to the excitatory postsynaptic current (EPSC) evoked by the activation of the Schaffer collaterals or the perforant path and determined the ratio of the total charge carried by AMPA vs. NMDA receptors by calculating the areas under the EPSC curves corresponding to the two types of receptor. We replicated their experiments in silico (see Methods for details) and determined the AMPA/NMDA conductance ratio that was necessary to reproduce the measured total charge ratio.

We used the Oblique Integration Test of HippoUnit (with custom NMDA receptor models and AMPA/NMDA conductance ratios) to analyze how the voltage dependence of NMDA receptor activation shapes the nature of synaptic integration in thin dendrites of CA1 PCs, and to determine the voltage dependence that provides the closest match to the experimental data. First, we evaluated models with eight distinct NMDA receptor voltage dependences from different published studies, including Jahr and Stevens (1990), which is the source of the default NMDA receptor model in HippoUnit; Major et al. (2008); Ujfalussy and Makara (2020), in which voltage dependence was deliberately placed between those of the Jahr-Stevens and Major models; Grunditz et al. (2008), Otmakhova et al. (2002), Larkum et al. (2009), Andrásfalvy and Magee (2001), and Gao et al. (2010).

We ran the Oblique Integration Test (Sáray et al., 2021) using each of these voltage dependences (Figure 5B) but otherwise identical synaptic parameters, and compared the resulting features, error scores, and the individual voltage traces. The test first selects candidate dendrites with the required geometric properties, then creates an active dendritic spine at the chosen location, which will receive the synapses. Next, it determines the synaptic weight (which scales the maximal conductance of both AMPA and NMDA receptors) such that a jump in the maximal steepness of the somatic voltage response (dV/dt) occurs as the number of activated synapses is increased from 4 to 5, which was a typical scenario in the experiments of Losonczy and Magge (2006). This jump is typically associated with the appearance of a dendritic sodium spike in the stimulated dendritic branch. Then it runs the main simulation experiments where increasing numbers of synapses are activated either synchronously or asynchronously on a given dendritic segment, and the somatic and dendritic voltages are recorded. Finally, it calculates various features such as the apparent voltage threshold for supralinear behavior, the amplitude of the response at threshold, and the degree of nonlinearity at and above the threshold.

**Figure 5:**
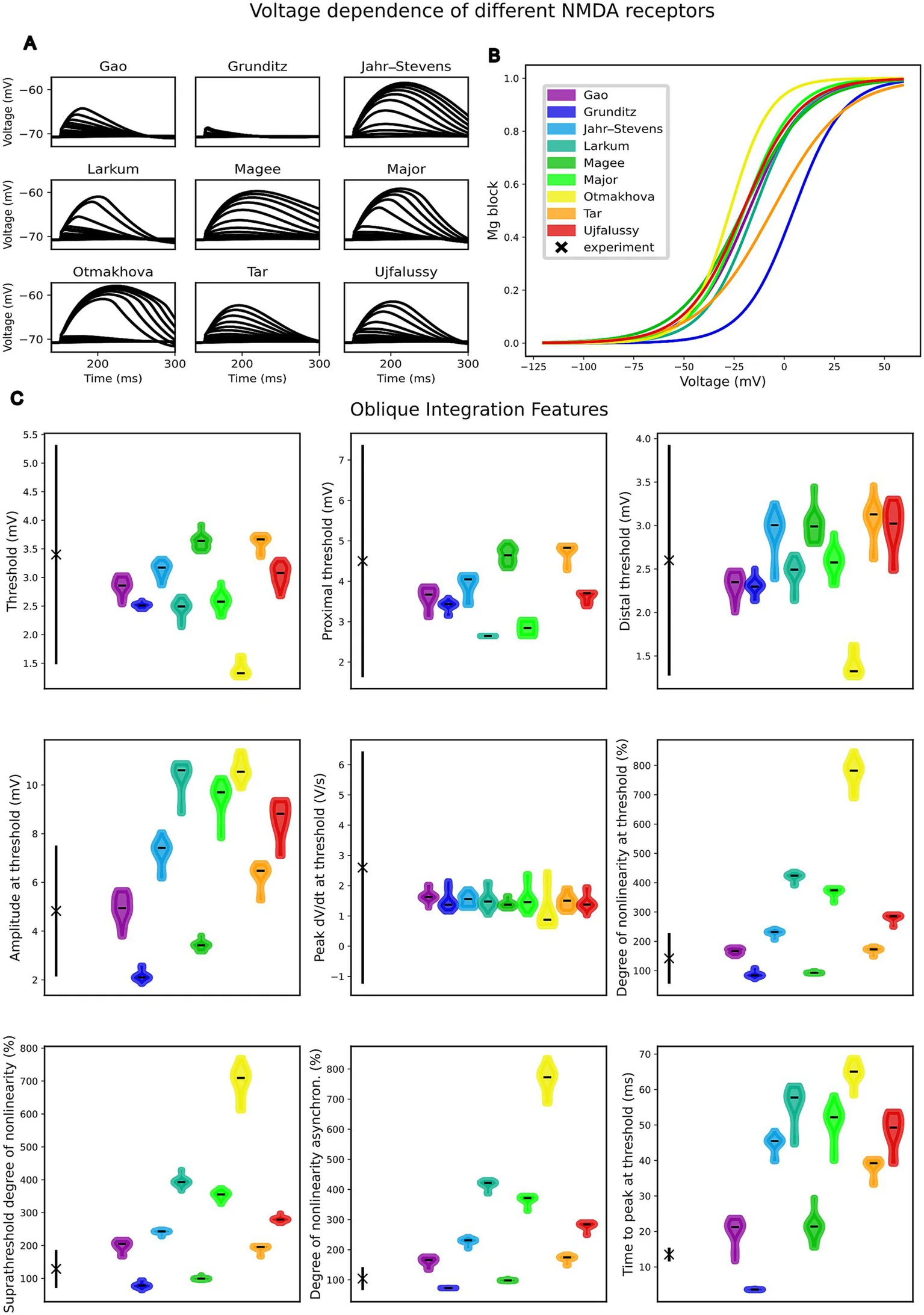
Modeling the voltage-dependence of NMDA receptors. **(A)** An example of the somatic voltage traces produced by HippoUnit’s Oblique Integration Test using different NMDA receptor models, where an increasing number of nearby synaptic inputs were activated synchronously on a distal dendrite. **(B)** Voltage-dependence of the different NMDA receptor models. The color code in the legend also applies to panel **(C)**, which shows the features of the Oblique Integration Test for all versions of the model with different voltage-dependences of the NMDA receptor. The experimental mean values are indicated by the black crosses in panel **(C)** (with the associated standard deviations indicated by the black bars).

We found large differences between the results of the Oblique Integration Test between models with different voltage dependences in the NMDA receptor, both at the level of the actual voltage responses to synaptic stimuli (Figure 5A) and in the values of several calculated features (Figure 5C). The voltage dependence of the NMDA receptor had a particularly strong effect on all the features that measure the degree of nonlinearity: some models showed a much greater degree of supralinearity than real neurons, some others remained sublinear, and only in a few cases did the degree of nonlinearity match the experimental results. The EPSP amplitude at threshold was similarly variable. On the other hand, some other features, such as the peak dV/dt at threshold, were not significantly affected by the voltage dependence of the NMDA receptor because this feature reflects the effect of dendritic Na spikes and depends mainly on the amount and properties of dendritic Na channels.

When looking at the somatic voltage responses to varying numbers (1–10) of synchronously activated synapses (Figure 5A), we observed prominent differences in the appearance of the long-lasting, large-amplitude component of the response that reflects non-linear activation of NMDA receptors (NMDA plateaus). In some cases, no NMDA plateau could be seen (Gao, Grunditz); in other cases, the NMDA plateau appeared only at input numbers much larger than the threshold (Larkum, Major, Ujfalussy, Magee); finally, in some cases, there was a very large jump in the response amplitude due to the appearance of the plateau, with little further increase as the number of synapses increased further (Otmakhova). None of these patterns matches the experimental observations of Losonczy and Magge (2006) and therefore rules out the corresponding NMDA receptor voltage dependence in our model. However, we note that some of these models still achieved reasonably high scores on the Oblique Integration Test, which suggests that the features currently used in this test may not completely cover all the relevant characteristics of nonlinear dendritic integration in CA1 PCs.

Overall, we found that none of the previously proposed voltage dependences led to completely satisfactory results in our model, so we decided to tune the parameters of the sigmoid function to achieve better results. By systematically changing the half-maximal point and the steepness of the sigmoid, we found a voltage dependence that led to oblique integration behavior matching experimental data both qualitatively and quantitatively (according to the scores of the Oblique Integration Test of HippoUnit). We found that the ideal NMDA receptor model for our model cell activates roughly at the same voltages as the receptor model of Ujfalussy et al. but then continues flatter, having a half-activation at −6 mV and at higher voltages it looks more like the NMDA receptor model of Grunditz et al. (2008) (Figure 5B) With this voltage dependance the model could replicate the jump driven by a dendritic sodium spike at 5 inputs and had the desired degree of nonlinearity while avoiding all of the previously mentioned issues regarding the amplitude of the NMDA component at higher input numbers.

Our results suggest that the voltage-dependence of the NMDA receptor has a major impact on the characteristics of synaptic integration in the dendrites of CA1 PCs. We found that a voltage-dependence that was different from previously suggested ones was needed to match both the qualitative and the quantitative features described in the experiments. However, although we believe that approaches based on combining different types of experimental data with morphologically and biophysically detailed modeling can potentially reveal the true biophysical properties of the NMDA receptor, we see our current model of the NMDA receptor as an effective model that works well in our neuronal model, and suggest that further, more specific studies will be necessary to address the properties of the NMDA receptor in general.

#### The roles of specific ion channels in the model

To verify that the ion channel models have the same effect in the model as the corresponding channels in a real cell, we systematically blocked each of them and re-evaluated the HippoUnit tests in each case. The results of these simulations are summarized in Table 5 and Supplementary Figure 9.

**Table 5:** Each row contains the results of a blocked channel model.

Blocked channel	Experimentally measured effect	Effect(s) in model	
		Soma	Dendrites
Sodium (Na)	No firing	No firing	No firing
Delayed rectifier potassium (KDR)	Impaired repolarization after spike (Hoffman et al., 1997; Coetzee et al., 1999), negative shift in voltage threshold for action potentials (APs) and reduced rheobase (Guan et al., 2007) increased initial firing frequency, depressed maintained firing because neurons entered depolarization block more readily (Liu and Bean, 2014)	Impaired repolarization and disarranged spiking pattern	bAP: slight change in spike widths on dendrites
Hyperpolarization-activated mixed cation (Ih)	Elimination of sag at hyperpolarizing current, increased EPSP amplitude and duration (Magee, 1998)	Elimination of sag at hyperpolarizing currents	
M-Type potassium (KM)	Spontaneous firing (Shah et al., 2011)	Spontaneous firing after stimulus, repetitive firing for small positive currents	
A-type potassium (KA)	Broad, irregular action potential shape, no attenuation of bAP, bigger EPSP (Hoffman et al., 1997; Cai et al., 2004; Gasparini et al., 2007)	Disrupted firing pattern and broad, irregular action potential shape	No attenuation of bAP
D-type potassium (KD)	Effect on the delay in the onset of firing (Storm, 1990) modulation of ADP and bursting (Metz et al., 2007)	Repetitive firing to very small positive currents, elimination of delay in the onset of firing	
N-type calcium (CaN)	No ADP and burst firing (Metz et al., 2007); also expecting similar effects as BK channel blocking	Elimination of fast AHP, firing doublets, strange spike shape	
BK-type calcium-dependent potassium	Effect on fast AHP and repolarization of spikes and plateaus (Marrion and Tavalin, 1998; Poolos and Johnston, 1999)	Elimination of fast AHP, firing doublets, strange spike shape	Very broad spikes on soma as the result of synaptic input
L-type calcium (CaL)	No activation of slow AHP, role in synaptic integration (Metz et al., 2007)	No activation of slow AHP	
Slow AHP potassium	Elimination of slow AHP	No activation of slow AHP which led to slight change in the spiking pattern	
T-type calcium (CaT)	Impact on pacemaking and repetitive firing (Metz et al., 2007)	Fewer action potentials, visible at 0.15 nA and 0.2 nA as an impact on pacemaking and repetitive firing	
SK-type calcium-dependent. Potassium	Elimination of medium AHP (Vogalis et al., 2003), effect on spike timing precision (Stocker, 2004),	More spikes at 0.1 and 0.15 nA as a result of the channel’s regulating medium AHP and spike timing precision	
R-type calcium (CaR)	Impact on burst firing under synaptic excitation, but no visible effect under somatic current injection (Metz et al., 2007)	No visible effect under somatic current injection	

The second column contains the expected effect of the blocking based on the experimental literature, while the third and fourth columns contain the effect on the model’s soma and dendrites, respectively. First, the Somatic Features Test was re-evaluated, and then the Backpropagating action potential test. In those cases where the fourth column regarding the dendritic behavior was left empty, there was either no effect on the dendritic behavior or the somatic behavior changed so much that the individual effect on the dendrites was inseparable.

In most cases, the effects of these manipulations in the model matched our expectations based on the experimental literature. Blocking sodium channels completely prevented the model from firing action potentials. Several potassium channels, including delayed-rectifier, A-type, and BK-type K channels contribute to action potential repolarization in CA1 PCs, and blocking any of these conductances in the model significantly altered both the action potential shape and the firing pattern. Blocking A-type potassium channels, which have the highest density in distal apical dendrites, also had a large impact on back-propagating action potentials, effectively eliminating the distance-dependent reduction of AP amplitude in apical dendrites. Blocking M-type and D-type potassium channels changed the spiking pattern (including spike frequency adaptation and the delay to the first spike) and also affected the excitability of the neuron (including its resting potential). The spiking pattern and the shape of the spike after-hyperpolarization were also changed in characteristic ways by blocking the calcium-dependent SK-type and slow AHP potassium channels.

The hyperpolarization-activated mixed cation current (Ih) is responsible for the voltage sag observed during negative current injections, and blocking these channels in the model led to the elimination of the sag response.

Blocking T-type calcium channels had a more visible impact on lower current injection amplitudes, where it resulted in the model firing fewer action potentials. Blocking R-type calcium channels had no visible effect during somatic current injections. Since this channel mainly affects burst firing, which cannot be elicited with somatic current injections in CA1 PCs, seeing no change under these circumstances was the expected outcome. Finally, certain types of Ca channels are structurally and functionally associated with specific calcium-dependent K channels, and therefore blocking these Ca channels also prevents the activation of the associated K channels. More specifically, we found that (as a result of their strong Ca-concentration-mediated coupling in the model), blocking N-type Ca channels had an effect that was similar to the effect of blocking BK-type K channels, and blocking the L-type Ca conductance had a similar effect to blocking the slow-AHP potassium conductance.

Completely blocking channels is an extreme form of manipulation that often pushes the model completely outside the physiological range. In order to probe how sensitive the model is to the exact values of the parameter that we tuned, we also performed more gradual perturbations of the model. More specifically, we individually changed all density-like parameters (including those expressed as a scaling factor for non-uniform densities) and the parameter representing the slope of the steady-state activation function of KA by either +20% or −20%, and shifted all membrane potential-like parameters by either +5 mV or −5 mV. We re-evaluated each of these modified models using the tests of HippoUnit, and calculated the change in the error for every test feature (Supplementary Figures 10–13). We found that there were a few critical parameters (such as the density and properties of voltage-gated Na channels) that had a dominant effect on the physiological responses of the model and affected the majority of features in every test. Some of these manipulations even prevented some of the tests from working properly by making the neuron either spontaneously active or completely silent. However, most other perturbations had more selective effects, influencing the model’s performance only in certain tests and a limited set of features.

#### The effect of explicitly modeling dendritic spines on the behavior of the model

All the results that we have presented so far were obtained with models in which most dendritic spines were not explicitly modeled. More specifically, for the parameter optimization and for all validations except the Oblique Integration Test, we modeled all dendritic spines indirectly, essentially merging their membrane surface (including all the spine-associated conductances) into the model of the corresponding dendritic segment (see Methods). For the Oblique Integration Test, we explicitly modeled all the spines that received synaptic input at any stage during the test, but we still modeled the rest of the spines using the F-factor method (modified to exclude the explicitly modeled spines; see Methods). The main reason for using these morphologically simplified models in these in silico experiments was that explicitly modeling tens of thousands of spines increases the computational demands of simulating the model (including the simulation time) by more than an order of magnitude. This becomes a serious issue when many simulations are required, such as during parameter optimization, even with the use of high-performance computing resources. However, to validate our methodology, we carried out an explicit comparison of different versions of our final models with either explicitly or indirectly modeled dendritic spines.

We constructed and simulated three different versions of the models. In the first version, all the spines were modelled indirectly with the F-factor method. This was our baseline model, and this was also how the parameters were fitted during the optimization process. In the second case, only those spines that received synaptic input were explicitly modelled. These models effectively differed from the first case only in tests where there was synaptic input, i.e., the Oblique Integration Test. Our third case was where all dendritic spines were individually modelled, whether they got synaptic input or not. In this case, all the HippoUnit tests were re-evaluated.

In the Somatic Features Test the optimized models and the models where all spines were present behaved very similarly. We found only a slight difference in the resting membrane potential (voltage base), where the models with all the spines were hyperpolarized by 0.5–1 mV compared to the optimized model. Every other difference was the indirect result of this hyperpolarization. Its origin can be traced back to the T-type calcium channels that are active at the resting membrane potential, and some of them were relocated to the spine heads in the all-spine case (Supplementary Figure 14).

In the case of the Backpropagating Action Potential Test, the PSP attenuation test, and the Depolarization Block Test, the cells that had all their spines modeled explicitly behaved in exactly the same way as the optimized models (Supplementary Figure 15).

However, more differences could be found between the models in the Oblique Integration Test. We compared three different cases: one where no spines were modelled, and the synapses targeted the dendritic shaft, one where only the spines that received synapses were modeled, and a third where all the dendritic spines were modeled. In the second case, we modified the Oblique Integration Test to first put a spine at the chosen dendritic location before connecting the synapse to the spine head. Test results and comparisons of the optimized models versus the spiny models can be seen in Figure 6 and Supplementary Figure 16. Figure 6B shows that in the cases when synapses arrive at the spine head, it does not matter if we model all dendritic spines or just the ones receiving synapses. However, the results were clearly different between the cases when synapses arrived at the shaft versus the spine. The degree of nonlinearity at threshold in the case when synapses arrived at the dendritic shaft was lower than the experimental data, but when synapses arrived at the spines, signal integration became supralinear. We investigated the individual traces to determine the reason for this difference. We found that the sodium spike at the beginning of the voltage response has a slightly lower amplitude when synapses arrive at the spine heads, but the NMDA component has a higher amplitude and longer duration (Figure 6A), which is responsible for stronger nonlinearities in signal integration.

**Figure 6:**
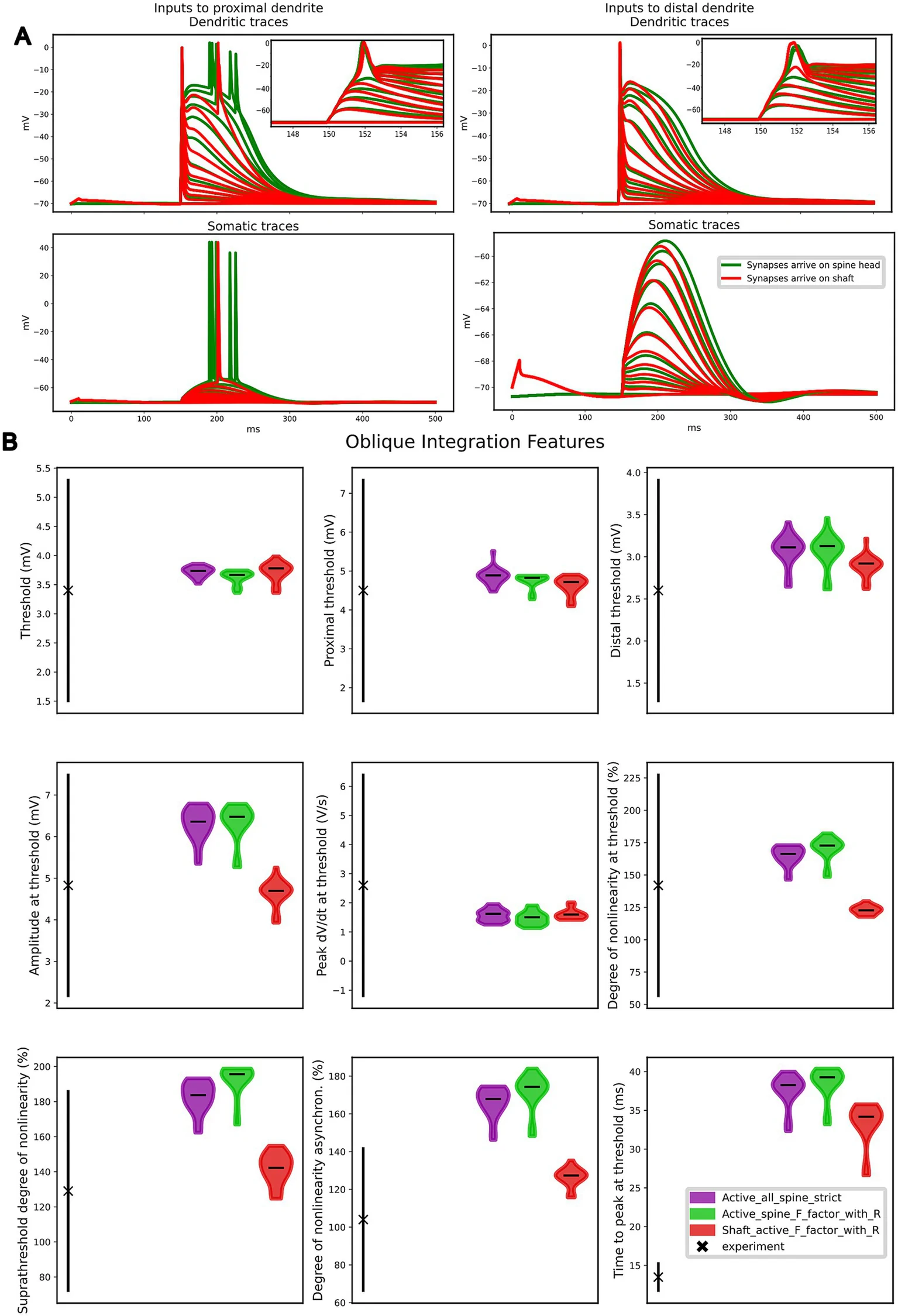
The effects of dendritic spines on the integration of synaptic inputs in oblique dendrites. **(A)** Examples of the voltage traces resulting from running the Oblique Integration Test of HippoUnit. The first row shows the dendritic traces when inputs target the proximal part of the dendrite (first column) or the distal part of the same dendrite (second column), and the second row shows the resulting voltage measured on the soma. Red traces correspond to the version of the model without explicitly modeled dendritic spines, where synapses arrive at the dendritic shaft, and green traces show the results when the synapses targeted the head of a spine connected to the dendrite at the same location. Insets in the first row show a magnified view of the early portion of the response. **(B)** Validation results of the Oblique Integration Test in 3 different cases. Red: Results from our optimized models, where all spines were modeled with the F-factor method and synapses targeted the dendritic shaft; Green: Those spines that received synaptic input were modeled explicitly, and others were taken into account by the F-factor method; Purple: All spines were modeled explicitly.

## Discussion

In this paper, we described the systematic and detailed modeling process that allowed us to build a new up-to-date model of CA1 pyramidal neurons that captures a wide variety of different cell behaviors and is expected to be useful in many different modeling scenarios. In addition to *in silico* studies of hippocampal pyramidal cell physiology, the model can be used, among others, as a key component of realistic models of hippocampal network dynamics and function (Bezaire et al., 2016; Romani et al., 2024), but also as a basis for studying the interactions between biophysical and biochemical processes in various neuronal compartments, including dendritic spines.

Unlike most previous models of CA1 PCs that were designed to study specific phenomena, our model was explicitly built to enable diverse applications. This was achieved, on the one hand, by constructing the model from carefully curated and calibrated components such as its morphology (including dendritic spines), intrinsic biophysical mechanisms and synaptic inputs, and, on the other hand, by systematically validating the physiological behavior of the model in a variety of different settings. The resulting models reproduce the relevant experimental results regarding the subthreshold and spiking voltage response to somatic current injections, the amplitude of back-propagating action potentials, the attenuation of single synaptic inputs and the nonlinear integration of spatially and temporally clustered synaptic inputs.

One crucial aspect of the new model is its updated and carefully evaluated set of ion channel models, which should be a useful resource in itself for the hippocampal and the broader neural modelling community. Our results also highlight the need for more high-quality, cell-type-specific experimental data on the key properties of ion channels, including their molecular identity, intracellular spatial distribution, and gating via the membrane potential, ionic concentrations, and various biochemical modulators.

Our results also demonstrate the utility of our previously introduced software tools for the automated optimization of model parameters (Mohácsi et al., 2024) and for the systematic validation of single cell physiological behavior (Sáray et al., 2021), and we hope that the current showcase will encourage the modeling community to include these or other similar tools in their workflows, which in turn would increase the reproducibility, robustness and transparency of computational neuroscience research.

### Modeling dendritic spines

Dendritic spines contribute substantially to the membrane surface of cortical pyramidal cells, receive the majority of excitatory synaptic inputs, and are believed to play important roles in electrical and chemical signaling in these neurons (Tsay and Yuste, 2004; Yuste, 2011; Tønnesen and Nägerl, 2016; Matsuzaki et al., 2001). On the other hand, due to their large number, modeling all spines in detail increases the number of compartments and thus the computational cost of simulating the electrical behavior of pyramidal cells by at least an order of magnitude, even using simplified models of spine morphology. Therefore, many previous studies, starting from the seminal work of Rapp et al. (1992), used models that, rather than modeling each individual spine explicitly, captured their overall effect on dendritic electrical signaling by adjusting the membrane capacitance and membrane resistance of spine-bearing dendritic shafts to account for the surface of the corresponding spines. This approximation has been shown to work well when the spines are passive (i.e., they contain no voltage-dependent mechanisms) and the spine head and the spine base are approximately isopotential, which generally holds in the absence of external (synaptic) input to the spines (Cornejo et al., 2022; Yuste, 2013; Palmer and Stuart, 2009). Several studies have also successfully applied a hybrid approach where spines receiving synaptic input are modeled explicitly while the rest are taken into account via the F-factor method (Eyal et al., 2018), and we also followed this route in most of our simulations.

However, the spines of CA1 PCs are known to be active, contain several voltage-gated and calcium ion concentration-dependent ion channels, and receive synaptic inputs that are partly mediated by voltage-dependent NMDA receptors. We aimed to capture these mechanisms in our detailed model, and we therefore developed and tested a version of the model where all spines were explicitly modeled, including these nonlinear mechanisms. We compared the behavior of this full model with the simplified version that resulted from parameter optimization, where the spines were omitted but, analogously to the F-factor method, all the conductances (including active and passive ones) as well as the capacitance of the spine were merged into the model of the dendritic shaft. Although some previous studies suggested that this method may lead to incorrect results (Zhang et al., 2023; Lagache et al., 2019), we found that the two versions of our model gave almost indistinguishable results regarding the somatic voltage response to somatic current injections, the attenuation of single postsynaptic potentials from various dendritic sites to the soma, and even the backpropagation of somatic action potentials into the apical dendrites. On the other hand, the model without any explicitly implemented dendritic spines behaved quite differently from the full model when we looked at the integration of synaptic inputs to oblique dendrites. This paradigm is based on synaptic inputs to dendritic spines, and the experimental results, which are successfully replicated by the full model, are known to depend on various voltage-dependent mechanisms including the nonlinear NMDA response at the synapse. The localization of these mechanisms to the spine matters in this case because synaptic inputs to the electrotonically isolated, high-impedance spine head evoke large local voltage transients that strongly activate voltage-dependent mechanisms in the spine, leading to a boosted response there. Although this signal is then attenuated when it spreads to the dendritic shaft, it remains boosted compared to the effect of an identical synaptic input that arrives directly at the dendritic shaft, which evokes a smaller local response and thus causes much less activation of voltage-dependent mechanisms. On the other hand, voltage signals originating outside a particular dendritic spine (such as back-propagating action potentials) spread effectively from the dendritic shaft to the spine, causing a very similar activation of voltage-dependent mechanisms in the shaft and the spine. This is the likely reason why we did not see any significant effect of explicitly modeling spines compared to the generalized F-factor method in all the tests that do not involve the activation of synaptic inputs.

However, even in the Oblique Integration Test, results very similar to those of the full model could be obtained from a third version of the model where only the spines receiving synaptic input were modeled explicitly. The computational cost of this model is intermediate between the full model and the one without any spines, although the exact cost depends on the number of spines that receive active synapses. Therefore, models of this type can be a good choice when synaptic inputs are important, but the resources available for simulating a single neuron need to be kept reasonably low, which is the typical case for large-scale realistic network models, and also for exploring the parameter space of single neurons responding to realistic patterns of synaptic input. However, it should be noted that such hybrid models can be used only if it is known *a priori* which spines may receive synaptic inputs during the simulation.

### Challenges in automated parameter fitting

Building models systematically and fitting their parameters to experimental data while keeping all the other properties of the chosen cell type that are not included in the parameter fitting process in mind raises several challenges. Our final set of models was created by optimizing neuronal parameters (mostly the densities and, in a few cases, the properties of ion channels) to match the somatic voltage response of real CA1 PCs to somatically injected step currents, as measured by a carefully chosen (sufficiently rich, but not strongly redundant) set of physiological features. Parameter optimization was carried out in a fully automated manner; however, a substantial amount of human expertise was still required to set up the optimization, e.g., to define the mechanisms included in the model, to choose the parameters to be optimized as well as their plausible ranges, and to set the values of the fixed (non-optimized) parameters. Our choices were driven mainly by the available experimental data, which were utilized in three distinct but complementary ways: first, to constrain (optimized and non-optimized) model parameters; second, to define target values for optimized model features; and third, to define further quantitative tests that we used to validate the behavior of the optimized models in diverse paradigms. Optimization and validation targets were kept fixed during model development, but our assumptions about model parameters were revised several times based on the results of model optimization and validation.

There was substantial diversity in the recordings available for the extraction of the feature values used as the targets for optimization and somatic feature validation. Since the target values correspond to the mean of the experimental features (with the error scale defined by the standard deviation), including qualitatively different recordings (with a possibly multimodal distribution of some features) could result in large standard deviations and mean feature values that are far from the feature values that characterize any of the recorded neurons. To avoid this situation, we excluded some atypical recordings, resulting in experimental data that were qualitatively similar but still sufficiently diverse at the quantitative level.

The diversity of the recordings (that remained even after removing outliers) also caused some technical issues that we needed to handle. In particular, we encountered cases where a given current injection evoked a spike (or several spikes) in some recordings but no spike in other recordings (from the same or a different neuron). In these cases, the mean values of action potential features across all the recordings at this current amplitude could not be defined in a meaningful way, and we evaluated models only on the features that were valid for all experimental recordings. On the other hand, when all the experimental traces for a certain stimulus contained action potentials but the model did not fire and thus errors in spike features could not be computed (and did not contribute to the total error), we added a large penalty (+100) to the final error score to make sure that models could not achieve a better score simply by not firing action potentials.

To fit the parameters of the models, features from the Electrophys Feature Extraction Library (eFEL) were used instead of using the voltage traces directly, which makes parameter fitting more reliable and robust (Almog and Korngreen, 2016; Almog and Korngreen, 2014; Druckmann et al., 2007; Hayes et al., 2005). eFEL is a validated and widely used library that contains a large variety of features, which allowed us to cover many aspects of the voltage response rather than focusing on a few hand-picked features that could easily lead to overfitting. However, even with this wide range of features, sometimes we still encountered model behaviors that seemed acceptable based on the feature values and resulted in a small overall error score, but a trained human observer could immediately see that something was amiss. In one case, the size and shape of the afterdepolarization in the tuned models were clearly different from the data, but this was not properly captured by the features calculated by eFEL. In another case, the best-fitting models matched the experimental spike pattern by producing a visible calcium spike in the somatic trace, although there was no sign of a slow calcium spike in the experimental recordings, and it is known that CA1 PCs do not tend to produce Ca spikes in response to weak somatic current injections (Golding et al., 1999). In these cases, after carefully evaluating the causes of the strange behavior, the model’s boundaries needed to be adjusted in a way that might not lead to an improvement in the error scores. In some cases, we also needed to adjust boundaries when the optimization algorithm consistently found solutions that were very close to one edge of the allowed parameter range, which could indicate that our initial guess regarding the plausible range of that parameter was incorrect, and extending the range could lead to better solutions.

Finally, optimizing the model for somatic features does not constrain parameters that have more impact on dendritic behavior but have little effect on what is happening at the soma. To obtain realistic results for dendritic behavior, the properties of the channels that strongly affect dendritic excitability (mainly the SK-channels and R-type calcium channels) or the boundaries corresponding to their densities (which were optimized parameters) had to be adjusted, and the parameter fitting based on somatic behavior was repeated.

In conclusion, even with our current workflow relying on automated tools, building good models still needs a lot of human input, and several rounds of optimization and validation may be necessary, which can be rather time-consuming both in terms of computations and human effort. One solution could be an extended multi-objective approach in which somatic features and dendritic behavior can be fitted at once, but this would heavily increase the computational demands, and new tools should be developed, or the current tools should be extended to support this scenario.

### The performance of models on the tests of HippoUnit: challenges and limitations

We used five previously created tests implemented by the HippoUnit package (Sáray et al., 2021) to evaluate models under several different conditions. The final models that we created achieved good scores on four of the five tests, while most of the models failed the Depolarization Block test. This test is designed to capture the experimental observation that sustained strong depolarizing input drives CA1 pyramidal cells (and many other types of neuron) into a state where, following a few initial spikes of decreasing amplitude, they stay depolarized but are unable to fire full-amplitude somatic action potentials. However, there is considerable variability between different experimental studies regarding the nature of the somatic membrane potential response during depolarization block. Several *in vitro* studies, including the one from which the target data for HippoUnit’s Depolarization Block Test originated (Bianchi et al., 2012), reported an essentially flat membrane potential during depolarization block, while others showed smaller somatic spikelets that likely reflected axonal or dendritic spikes that failed to induce full amplitude somatic action potentials due to the sustained inactivation of somatic sodium channels (Apostolides et al., 2016). Our models consistently showed the latter behavior, which was inconsistent with the results of Bianchi et al. (2012). The few models that passed the test had qualitatively similar behavior but slightly smaller spikelets whose amplitude was below the detection threshold of the test. The cause of these qualitative differences between different experimental studies is currently unclear, but differences between *in vitro* and *in vivo* conditions, and also in recording conditions (temperature, electrodes, solutions) between different *in vitro* studies, are likely candidates.

We found that the spikelets during depolarization block in the model are caused by full amplitude action potentials in the axon, and the axon (and especially its initial segment) has been identified as a possible source of somatic spikelets in other studies as well (Apostolides et al., 2016). Therefore, we expect that the (morphological and biophysical) properties of the axon will have a large impact on the generation of spikelets during depolarization block, and a more realistic model of the axon could be used to investigate this matter further.

The resting membrane potential of neurons can vary significantly even within a given cell type, and it can also change as a result of synaptic inputs, neuronal activity, or experimental manipulations (including the effects of recording electrodes). The recordings that we used as the target for the Somatic Features Test standardized the baseline voltage by injecting a constant current to achieve a membrane potential around −65 mV before the injection of current steps. This experimental manipulation was not replicated in our simulations; instead, a resting potential around −65 mV was among the target features for parameter optimization, and this target was successfully achieved in all of our optimized models.

A potentially related issue regarding the Oblique Integration Test was that, in many cases, combinations of synaptic inputs to oblique dendrites that triggered supralinear dendritic integration also evoked somatic action potentials, which had a dominant effect on the amplitude of the somatic voltage response. The target data from Losonczy et al. did not seem to contain somatic APs, although it was not clear whether this was due to any filtering or manipulations (such as current injections). Therefore, we injected a small negative constant current into the somata of our models while running the Oblique Integration Test, which significantly reduced the occurrence of somatic spikes and increased the number of dendritic segments from which meaningful results could be obtained.

Validating our optimized models also revealed one shortcoming of the Oblique Integration Test of HippoUnit. We observed that some models that achieved good scores on most features of this test and whose behavior also appeared acceptable upon visual inspection of the voltage traces, nevertheless received a high error score on the “time to peak at threshold” feature. We found out that this was due to the relevant voltage signal having two components: the fast sodium spike component and the slower NMDA component. HippoUnit currently measures the latency of the highest peak, which may correspond to either the fast or the slow component, depending on the overall shape of the voltage response of the model, while the data from the study of Losonczy and Magge (2006) always refer to the latency of the fast component. This results in an artificially inflated error for models where the overall highest voltage is achieved during the NMDA component of the response. This problem could be solved by modifying the Oblique Integration Test of HippoUnit to separate the two components of the signal and always measure the timing of the first component.

One important general conclusion of our study is that building high-quality general-purpose models requires the evaluation of model behavior in diverse settings. HippoUnit currently consists of five tests that validate different aspects of CA1 PC physiology, and such diverse testing was critical for sufficiently constraining the unknown parameters of the model. For example, we found that optimized CA1 PC models with NMDA receptor models from previous studies could achieve good scores on the Somatic Features Test, the PSP Attenuation Test, and the Oblique Integration Test if the density of dendritic Na channels was assumed to be relatively high; however, these models performed badly on the Backpropagating Action Potential Test. On the other hand, by modifying the voltage dependence of the NMDA receptor model and using a lower density of dendritic Na channels, we could build models that performed well simultaneously on all of these tests. Finally, although we believe that using the current tests of HippoUnit one can constrain most parameters well, and build models that generalize to novel situations, extending HippoUnit with more tests could lead to further improvements in model quality and allow the verification of model behavior in other relevant scenarios.

### Further modelling opportunities

#### Morphology

Nowadays, many reconstructed rat CA1 pyramidal cell morphologies are available, and several recent modelling studies used more than one morphology to strengthen their conclusions (Migliore et al., 2018; Reva et al., 2023; Tomko et al., 2021). For this study, we used only one morphological reconstruction from Megías et al. (Megías et al., 2001) because of the high computational demands of parameter optimization and validation, especially for the more detailed versions of the models. However, it would be interesting to see how using different morphologies influences the results. In general, we expect that major differences in morphology need to be compensated by altered biophysical properties (such as channel densities) to achieve similar physiological behavior, so that models with different morphology would need to be tuned separately. Studies that examined how switching to a different morphology while keeping the same channel densities affected the performance of models found limited generalization (Migliore et al., 2018; Reva et al., 2023; Arnaudon et al., 2023). Therefore, simply re-evaluating our model with one or a few other morphologies may not lead to robust conclusions (the results could depend on the morphologies selected), and a reliable study of these issues would likely require tuning a large set of models with different morphologies and then examining how variations of anatomical and biophysical properties influence the various physiological characteristics of the neurons. We also note that systematic errors in the morphological reconstructions are expected to have a major impact on the accuracy of the modeling results, so any extension of this work should be based on morphologies that are similarly accurate, especially regarding dendritic diameters.

#### Ion channels

Ionic currents in our model are described by a conductance-based formalism or, in the case of Ca currents, by a permeability-based approach captured by the Goldman-Hodgkin-Katz equations. In both cases, the open probability of the channels is described by the formalism introduced by Hodgkin and Huxley (1952), which essentially assumes that the opening and closing of channels is due to the operation of several, independently operating gates, and the open probabilities of individual gates (the gating variables) follow first-order kinetics with transition rates that may depend on the membrane potential or other variables. Among several possible parameterizations of the gating equations, one that is closely tied to the results of typical voltage-clamp experiments was suggested by Borg-Graham (1991). We used this formalism to describe the gating of most channels in our model, and set the parameters to capture the experimentally measured voltage dependence of the steady state value and time constant of channel activation and inactivation.

However, recent experimental data indicate that these types of channel models may not fully capture the behavior of certain ion channels; for instance, channel activation and inactivation may not be independent processes (Mitrovic et al., 1998; Pan et al., 2011; Cuello et al., 2010). A more general formalism for describing ion channels is based on Markov chains, which explicitly model the transitions between their states. There are several ion channels in CA1 PCs for which implementations as Markov-type models are available (Lampert and Korngreen, 2014; Siekmann et al., 2016; Sansom et al., 1989). However, the parameters of Markov-type models are not directly related to electrophysiological measurements, and may not even be sufficiently constrained by the data that are available for most channels. Markov-type channel models also tend to require more computational resources than Hodgkin-Huxley-type models. For these reasons, we used Hodgkin-Huxley-type models of all voltage-gated channels in our model. On the other hand, it would be interesting to see how the model’s behavior changes if certain ion channels are changed to potentially more accurate Markov-type channel models.

Knowing the exact molecular composition of the channels responsible for ionic currents can potentially lead to better neuronal models. However, building a cellular model based on molecularly identified ion channels would require not only knowing the identities and total amounts of all the relevant ion channel proteins, but also their spatial distribution within the neuron, the exact ways they combine with other subunits (including modulatory ones), and the functional properties of the resulting channels that could be cell-type- and even state-dependent. All the required information is typically not yet available, and building models based on partial information can be counterproductive. For instance, we attempted to further improve our model by separately modeling the three molecular subtypes of T-type Ca channels that were detected in CA1 PCs. This approach seemed promising since we found quantitative data on the distribution of the three relevant channel proteins (Cav3.1, Cav3.2, and Cav3.3) (McKay et al., 2006), and another study described the voltage-dependent gating of Ca channels containing these subunits (Iftinca et al., 2006). However, these molecularly identified channels generated currents whose properties were different from those of T-type currents previously measured in CA1 PCs, and including these channel models in our cell model (and optimizing its parameters) led to unrealistic subthreshold physiological behavior. The discrepancies could be due to the cell-type-specific molecular composition and modulation of the channels, but more data (preferably from molecularly characterized channels in CA1 PCs) will be required to resolve the issue.

On the other hand, we indirectly used molecular information several times when we constructed our model. Modelling sodium channels, knowing that the dendritic, somatic, and axonal sodium channels have different kinetics (due to the distinct spatial distribution of Nav1.6 and Nav1.2 channel proteins in the cell) (Lőrincz and Nusser, 2010), improved the behavior of the model. Similarly, knowing that D-type potassium currents are generated by a heteromer of potassium channels, which, as homomers, mediate delayed-rectifier potassium currents (Coetzee et al., 1999) implied that a D-type potassium current may exist on the axon as well, which also improved our model.

There are also ion channels that were excluded from this model, but, following the ever-growing literature, it could be an interesting experiment to determine how including these channels would alter the model’s behavior. One of the candidates would be the inward-rectifier potassium channels that are also present in the dendrites and dendritic spines and influence the excitability of the cells (Chen and Johnston, 2005) and can play a role in synaptic plasticity mechanisms (Victoria et al., 2016). Modelling chloride channels are also a possible direction for adding further ion channel models since they play an important role in maintaining the balance between excitation and inhibition, shaping synaptic responses (Huang et al., 2012), and protecting against pathological conditions like ischemia and epilepsy (Gomez et al., 2019).

It should be noted that we used experimental data recorded at a physiological temperature (33 °C) for building the ion channels, parameter fitting and validation. Most of our ion channel models are not corrected for temperature, due to lack of data about their temperature dependance, therefore our models should be used and evaluated at body temperature.

#### Axonal characteristics

The least accurate part of our current model is probably its axon. This is important because the axon is typically the site of action potential initiation in CA1 PCs (Stuart and Sakmann, 1994; Palmer and Stuart, 2006; Kole et al., 2008; Leterrier, 2018), it has a major impact on somatic spiking behavior, and likely affects the appearance and characteristics of depolarization block as well. The main reason for the potentially lower quality of the model of the axon compared to the rest of the model is the relative sparsity of experimental data. The high-quality reconstructed morphology from Megías et al. (2001) that formed the anatomical basis of our model did not include the reconstruction of the axon, and we were unable to find a CA1 PC morphology with a similarly reliable reconstruction of both the dendrites and the axon. Therefore, we complemented our detailed soma-dendritic morphology with a simpler axonal morphology from the model of Bianchi et al. (2012). We did not follow Bianchi et al. (2012) regarding the biophysical properties of the axon; instead, we set the densities and properties of ion channels in the axon based on our detailed review of the relevant experimental literature (see Methods). This approach allowed our tuned models to reproduce many of the physiological features of CA1 PCs. However, the axonal morphology that we copied from Bianchi et al. (2012) lacks some important features of real CA1 PC axons. Most importantly, it does not contain a separate axon initial segment (AIS), which is known to be an anatomically and functionally distinct part of the axon where action potentials are typically initiated (Kole et al., 2008; Leterrier, 2018; Kole and Stuart, 2012; Rasband, 2010). Therefore, replacing our current model of the axon with a more realistic one that includes the AIS would allow us to accurately capture both the distinctive geometry and the special set of biophysical mechanisms in the AIS, which would (following a new round of parameter optimization) likely lead to further improvements in somatic spiking properties, and may also allow us to resolve the controversy regarding the generation of depolarization block in CA1 PCs.

## Conclusion

We hope that the modelling and experimental community will benefit from our new multi-purpose model of CA1 pyramidal neurons. According to the validation results the model can capture the behavior of these cells under somatic current injections (with the possible exception of depolarization block), the propagation of (passive and active) signals along the dendritic tree (both from and toward the soma), and integrates synaptic input on the oblique dendrites properly. In silico experiments requiring these features can be safely carried out on our model. On the other hand, the axonal behavior of the model can probably be improved by using a more detailed model of the axon and incorporating more specific experimental constraints (possibly including the development of a new, targeted HippoUnit validation for axonal voltage responses). The behavior of the model under *in vivo* conditions and as a component of network models also needs to be evaluated.

Most of our workflow should generalize to modeling other cell types and even other kinds of neural systems. The Neuroptimus tool has already been used to fit parameters of neuronal models of various types (Takács et al., 2024), as well as the parameters of subcellular biochemical pathways and synaptic parameters of large networks. While the current tests of HippoUnit are specific to CA1 pyramidal neurons, these tests can be easily adapted to other cell types. The most critical requirement (and potential bottleneck) for the construction of faithful data-driven models of different types of neurons is the availability of all the necessary types of experimental data, as the quality and quantity of these data determine the success of both model construction (and tuning) and model validation.

## Data Availability

The original contributions presented in the study are included in the article/Supplementary material, further inquiries can be directed to the corresponding author/s.

## References

[ref1] Almog M. Korngreen A. (2014). A quantitative description of dendritic Conductances and its application to dendritic excitation in layer 5 pyramidal neurons. J. Neurosci. 34, 182–196. doi: 10.1523/JNEUROSCI.2896-13.2014, 24381280 PMC6608163

[ref2] Almog M. Korngreen A. (2016). Is realistic neuronal modeling realistic? J. Neurophysiol. 116, 2180–2209. doi: 10.1152/jn.00360.2016, 27535372 PMC5102320

[ref3] Andrásfalvy B. K. Magee J. C. (2001). Distance-dependent increase in AMPA receptor number in the dendrites of adult hippocampal CA1 pyramidal neurons. J. Neurosci. 21, 9151–9159. doi: 10.1523/JNEUROSCI.21-23-09151.2001, 11717348 PMC6763889

[ref5] Apostolides P. F. Milstein A. D. Grienberger C. Bittner K. C. Magee J. C. (2016). Axonal filtering allows reliable output during dendritic plateau-driven complex spiking in CA1 neurons. Neuron 89, 770–783. doi: 10.1016/j.neuron.2015.12.040, 26833135

[ref6] Arnaudon A. Reva M. Zbili M. Markram H. Van Geit W. Kanari L. (2023). Controlling morpho-electrophysiological variability of neurons with detailed biophysical models. iScience 26:108222. doi: 10.1016/j.isci.2023.108222, 37953946 PMC10638024

[ref7] Ballesteros-Merino C. Lin M. Wu W. W. Ferrandiz-Huertas C. Cabañero M. J. Watanabe M. . (2012). Developmental profile of SK2 channel expression and function in CA1 neurons. Hippocampus 22, 1467–1480. doi: 10.1002/hipo.20986, 22072564 PMC3359419

[ref8] Bell D. C. Butcher A. J. Berrow N. S. Page K. M. Brust P. F. Nesterova A. . (2001). Biophysical properties, pharmacology, and modulation of human, neuronal L-type (α1D, CaV1.3) voltage-dependent calcium currents. J. Physiol. 85, 816–827. doi: 10.1152/jn.2001.85.2.816, 11160515

[ref9] Bernard C. Johnston D. (2003). Distance-dependent modifiable threshold for action potential back-propagation in hippocampal dendrites. J. Neurophysiol. 90, 1807–1816. doi: 10.1152/jn.00286.2003, 12966178

[ref10] Bezaire M. J. Raikov I. Burk K. Vyas D. Soltesz I. (2016). Interneuronal mechanisms of hippocampal theta oscillations in a full-scale model of the rodent CA1 circuit. eLife 5, 1–106. doi: 10.7554/eLife.18566, 28009257 PMC5313080

[ref11] Bianchi D. Marasco A. Limongiello A. Marchetti C. Marie H. Tirozzi B. . (2012). On the mechanisms underlying the depolarization block in the spiking dynamics of CA1 pyramidal neurons. J. Comput. Neurosci. 33, 207–225. doi: 10.1007/s10827-012-0383-y, 22310969

[ref12] Bloodgood B. L. Sabatini B. L. (2007). Nonlinear regulation of unitary synaptic signals by CaV2.3 voltage-sensitive calcium channels located in dendritic spines. Neuron 53, 249–260. doi: 10.1016/j.neuron.2006.12.017, 17224406

[ref13] Bologna L. L. Smiriglia R. Curreri D. Migliore M. (2021). The EBRAINS NeuroFeatureExtract: an online resource for the extraction of neural activity features from electrophysiological data. Front. Neuroinform. 15:713899. doi: 10.3389/fninf.2021.713899, 34512300 PMC8427185

[ref14] Borg-Graham L. (1991). Modelling the non-linear conductances of excitable membranes. Cellular Neurobiology 13, 247–275. doi: 10.1093/oso/9780199631063.003.0013, 37701723

[ref15] Borg-Graham L. J. (1999). “Interpretations of data and mechanisms for hippocampal pyramidal cell models,” in Cerebral Cortex, (New York: Plenum Press), 19–138.

[ref16] Bowden S. E. H. Fletcher S. Loane D. J. Marrion N. V. (2001). Somatic colocalization of rat SK1 and D class (Cav 1.2) L-type calcium channels in rat CA1 hippocampal pyramidal neurons. J. Neurosci. 21:RC175. doi: 10.1523/jneurosci.21-20-j0006.2001, 11588205 PMC6763866

[ref17] Cai X. Liang C. W. Muralidharan S. Kao J. P. Y. Tang C. M. Thompson S. M. (2004). Unique roles of SK and Kv4.2 potassium channels in dendritic integration. Neuron 44, 351–364. doi: 10.1016/j.neuron.2004.09.026, 15473972

[ref18] Carnevale N. T. Hines M. L. (2009). The Neuron Book. New York, NY, USA: Cambridge University Press.

[ref19] Chen X. Johnston D. (2004). Properties of single voltage-dependent K+ channels in dendrites of CA1 pyramidal neurones of rat hippocampus. J. Physiol. 559, 187–203. doi: 10.1113/jphysiol.2004.068114, 15218076 PMC1665081

[ref20] Chen X. Johnston D. (2005). Constitutively active G-protein-gated inwardly rectifying K+ channels in dendrites of hippocampal CA1 pyramidal neurons. J. Neurosci. 25, 3787–3792. doi: 10.1523/JNEUROSCI.5312-04.2005, 15829630 PMC6724929

[ref21] Coetzee W. A. Amarillo Y. Chiu J. Chow A. Lau D. McCormack T. . (1999). Molecular diversity of K+ channels. Ann. N. Y. Acad. Sci. 868, 233–255. doi: 10.1111/j.1749-6632.1999.tb11293.x, 10414301

[ref22] Colbert C. M. Magee J. C. Hoffman D. A. Johnston D. (1997). Slow recovery from inactivation of Na+ channels underlies the activity-dependent attenuation of dendritic action potentials in hippocampal CA1 pyramidal neurons. J. Neurosci. 17, 6512–6521. doi: 10.1523/JNEUROSCI.17-17-06512.1997, 9254663 PMC6573147

[ref23] Cornejo V. H. Ofer N. Yuste R. (2022). Voltage compartmentalization in dendritic spines in vivo. Science 375, 82–86. doi: 10.1126/science.abg0501, 34762487 PMC8942082

[ref24] Cuello L. G. Jogini V. Cortes D. M. Pan A. C. Gagnon D. G. Dalmas O. . (2010). Structural basis for the coupling between activation and inactivation gates in K+ channels. Nature 466, 272–275. doi: 10.1038/nature09136, 20613845 PMC3033755

[ref25] Druckmann S. Banitt Y. Gidon A. Schürmann F. Markram H. Segev I. (2007). A novel multiple objective optimization framework for constraining conductance-based neuron models by experimental data. Fronit Neurosci 1, 7–18. doi: 10.3389/neuro.01.1.1.001.2007, 18982116 PMC2570085

[ref26] Eyal G. Verhoog M. B. Testa-Silva G. Deitcher Y. Benavides-Piccione R. DeFelipe J. . (2018). Human cortical pyramidal neurons: from spines to spikes via models. Front. Cell. Neurosci. 12:181. doi: 10.3389/fncel.2018.00181, 30008663 PMC6034553

[ref27] Foehring R. C. Mermelstein P. G. Song W. J. Ulrich S. Surmeier D. J. (2000). Unique properties of R-type calcium currents in neocortical and Neostriatal neurons. J. Neurophysiol. 84, 2225–2236. doi: 10.1152/jn.2000.84.5.2225, 11067968

[ref28] Francioni V. Harnett M. T. (2022). Rethinking single neuron electrical compartmentalization: dendritic contributions to network computation in vivo. Neuroscience 489, 185–199. doi: 10.1016/j.neuroscience.2021.05.038, 34116137

[ref29] Gao C. Gill M. B. Tronson N. C. Guedea A. L. Guzmán Y. F. Huh K. H. . (2010). Hippocampal NMDA receptor subunits differentially regulate fear memory formation and neuronal signal propagation. Hippocampus 20, 1072–1082. doi: 10.1002/hipo.20705, 19806658 PMC2891656

[ref30] Gasparini S. Losonczy A. Chen X. Johnston D. Magee J. C. (2007). Associative pairing enhances action potential back-propagation in radial oblique branches of CA1 pyramidal neurons. J. Physiol. 580, 787–800. doi: 10.1113/jphysiol.2006.121343, 17272353 PMC2075451

[ref32] Golding N. L. Jung H. Mickus T. Spruston N. (1999). Dendritic calcium spike initiation and repolarization are controlled by distinct potassium channel subtypes in CA1 pyramidal neurons. J. Neurosci. 19, 8789–8798. doi: 10.1523/JNEUROSCI.19-20-08789.1999, 10516298 PMC6782757

[ref33] Golding N. L. Kath W. L. Spruston N. (2001). Dichotomy of action-potential backpropagation in CA1 pyramidal neuron dendrites. J. Neurophysiol. 86, 2998–3010. doi: 10.1152/jn.2001.86.6.2998, 11731556

[ref34] Golomb D. Donner K. Shacham L. Shlosberg D. Amitai Y. Hansel D. (2007). Mechanisms of firing patterns in fast-spiking cortical interneurons. PLoS Comput. Biol. 3:e156. doi: 10.1371/journal.pcbi.0030156, 17696606 PMC1941757

[ref35] Gomez C. D. Read J. Acharjee S. Pittman Q. J. (2019). Early life inflammation increases CA1 pyramidal neuron excitability in a sex and age dependent manner through a chloride homeostasis disruption. J. Neurosci. 39, 7244–7259. doi: 10.1523/JNEUROSCI.2973-18.2019, 31308096 PMC6759023

[ref36] Grunditz Å. Holbro N. Tian L. Zuo Y. Oertner T. G. (2008). Spine neck plasticity controls postsynaptic calcium signals through electrical compartmentalization. J. Neurosci. 28, 13457–13466. doi: 10.1523/JNEUROSCI.2702-08.2008, 19074019 PMC6671740

[ref37] Gu N. Vervaeke K. Storm J. F. (2007). BK potassium channels facilitate high-frequency firing and cause early spike frequency adaptation in rat CA1 hippocampal pyramidal cells. J. Physiol. 580, 859–882. doi: 10.1113/jphysiol.2006.126367, 17303637 PMC2075463

[ref38] Guan D. Lee J. C. F. Higgs M. H. Spain W. J. Foehring R. C. (2007). Functional roles of Kv1 channels in neocortical pyramidal neurons. J. Neurophysiol. 97, 1931–1940. doi: 10.1152/jn.00933.2006, 17215507

[ref39] Harnett M. T. Makara J. K. Spruston N. Kath W. L. Magee J. C. (2012). Synaptic amplification by dendritic spines enhances input cooperativity. Nature 491, 599–602. doi: 10.1038/nature11554, 23103868 PMC3504647

[ref40] Hay E. Hill S. Schürmann F. Markram H. Segev I. (2011). Models of neocortical layer 5b pyramidal cells capturing a wide range of dendritic and Perisomatic active properties. PLoS Comput. Biol. 7:e1002107. doi: 10.1371/journal.pcbi.1002107, 21829333 PMC3145650

[ref41] Hayes R. D. Byrne J. H. Cox S. J. Baxter D. A. (2005). Estimation of single-neuron model parameters from spike train data. Neurocomputing 65–66, 517–529. doi: 10.1016/j.neucom.2004.10.039

[ref42] Hirschberg B. Maylie J. Adelman J. P. Marrion N. V. (1998). Gating of recombinant small-conductance ca-activated K+ channels by calcium. J. Gen. Physiol. 111, 565–581. doi: 10.1085/jgp.111.4.565, 9524139 PMC2217120

[ref43] Hodgkin A. L. Huxley A. F. (1952). A quantitative description of membrane current and its application to conduction and excitation in nerve. J. Physiol. 117, 500–544. doi: 10.1113/jphysiol.1952.sp004764, 12991237 PMC1392413

[ref44] Hoffman D. A. Magee J. C. Colbert C. M. Johnston D. (1997). K+ channel regulation of signal propagation in dendrites of hippocampal pyramidal neurons. Nature 387, 869–875. doi: 10.1038/43119, 9202119

[ref45] Hönigsperger C. Marosi M. Murphy R. Storm J. F. (2015). Dorsoventral differences in Kv7/M-current and its impact on resonance, temporal summation and excitability in rat hippocampal pyramidal cells. J. Physiol. 593, 1551–1580. doi: 10.1113/jphysiol.2014.280826, 25656084 PMC4386960

[ref46] Huang W. C. Xiao S. Huang F. Harfe B. D. Jan Y. N. Jan L. Y. (2012). Calcium-activated chloride channels (CaCCs) regulate action potential and synaptic response in hippocampal neurons. Neuron 74, 179–192. doi: 10.1016/j.neuron.2012.01.033, 22500639 PMC3329964

[ref47] Iftinca M. McKay B. E. Snutch T. P. McRory J. E. Turner R. W. Zamponi G. W. (2006). Temperature dependence of T-type calcium channel gating. Neuroscience 142, 1031–1042. doi: 10.1016/j.neuroscience.2006.07.010, 16935432

[ref48] Jahr C. E. Stevens C. F. (1990). Voltage dependence of NMDA-activated macroscopic conductances predicted by single-channel kinetics. J. Neurosci. 10, 3178–3182. doi: 10.1523/JNEUROSCI.10-09-03178.1990, 1697902 PMC6570236

[ref49] Jerng H. H. Pfaffinger P. J. (2014). Modulatory mechanisms and multiple functions of somatodendritic A-type K+ channel auxiliary subunits. Front. Cell. Neurosci. 8:82. doi: 10.3389/fncel.2014.00082, 24723849 PMC3973911

[ref50] Johnston D. Hoffman D. A. Magee J. C. Poolos N. P. Watanabe S. Colbert C. M. . (2000). Dendritic potassium channels in hippocampal pyramidal neurons. J. Physiol. 525, 75–81. doi: 10.1111/j.1469-7793.2000.00075.x, 10811726 PMC2269937

[ref51] Jung H. Y. Mickus T. Spruston N. (1997). Prolonged Sodium Channel inactivation contributes to dendritic action potential attenuation in hippocampal pyramidal neurons. J. Neurosci. 17, 6639–6646. doi: 10.1523/JNEUROSCI.17-17-06639.1997, 9254676 PMC6573150

[ref52] Káli S. Freund T. F. (2005). Distinct properties of two major excitatory inputs to hippocampal pyramidal cells: a computational study. Eur. J. Neurosci. 22, 2027–2048. doi: 10.1111/j.1460-9568.2005.04406.x, 16262641

[ref53] Kerti K. Lorincz A. Nusser Z. (2011). Unique somato-dendritic distribution pattern of Kv4.2 channels on hippocampal CA1 pyramidal cells. Eur. J. Neurosci. 35, 66–75. doi: 10.1111/j.1460-9568.2011.07907.x, 22098631 PMC3428895

[ref54] Kim J. Wei D. S. Hoffman D. A. (2005). Kv4 potassium channel subunits control action potential repolarization and frequency-dependent broadening in rat hippocampal CA1 pyramidal neurones. J. Physiol. 569, 41–57. doi: 10.1113/jphysiol.2005.095042, 16141270 PMC1464206

[ref55] King B. Rizwan A. P. Asmara H. Heath N. C. Engbers J. D. T. Dykstra S. . (2015). IKCa channels are a critical determinant of the slow AHP in CA1 pyramidal neurons. Cell Rep. 11, 175–182. doi: 10.1016/j.celrep.2015.03.026, 25865881

[ref56] Kise Y. Kasuya G. Okamoto H. H. Yamanouchi D. Kobayashi K. Kusakizako T. . (2021). Structural basis of gating modulation of Kv4 channel complexes. Nature 599:158. doi: 10.1038/s41586-021-03935-z, 34552243 PMC8566240

[ref57] Kole M. H. P. Ilschner S. U. Kampa B. M. Williams S. R. Ruben P. C. Stuart G. J. (2008). Action potential generation requires a high sodium channel density in the axon initial segment. Nat. Neurosci. 11, 178–186. doi: 10.1038/nn2040, 18204443

[ref58] Kole M. H. P. Stuart G. J. (2012). Signal processing in the axon initial segment. Neuron 73, 235–247. doi: 10.1016/j.neuron.2012.01.007, 22284179

[ref59] Lagache T. Jayant K. Yuste R. (2019). Electrodiffusion models of synaptic potentials in dendritic spines. J. Comput. Neurosci. 47, 77–89. doi: 10.1007/s10827-019-00725-5, 31410632 PMC7350286

[ref60] Lampert A. Korngreen A. (2014). Markov modeling of ion channels: implications for understanding disease. Prog. Mol. Biol. Transl. Sci. 123, 1–21. doi: 10.1016/B978-0-12-397897-4.00009-7, 24560138

[ref61] Larkum M. E. Nevian T. Sandler M. Polsky A. Shiller J. (2009). Synaptic integration in tuft dendrites of layer 5 pyramidal neurons: a new unifying principle. Science 325, 756–760. doi: 10.1126/science.1171958, 19661433

[ref62] Leterrier C. (2018). The axon initial segment: an updated viewpoint. J. Neurosci. 38, 2135–2145. doi: 10.1523/JNEUROSCI.1922-17.2018, 29378864 PMC6596274

[ref63] Lindroos R. Dorst M. C. Du K. Filipović M. Keller D. Ketzef M. . (2018). Basal ganglia neuromodulation over multiple temporal and structural scales—simulations of direct pathway MSNs investigate the fast onset of dopaminergic effects and predict the role of Kv4.2. Front. Neural Circuits 12:12. doi: 10.3389/fncir.2018.00003, 29467627 PMC5808142

[ref64] Liu P. W. Bean B. P. (2014). Kv2 channel regulation of action potential repolarization and firing patterns in superior cervical ganglion neurons and hippocampal CA1 pyramidal neurons. J. Neurosci. 34, 4991–5002. doi: 10.1523/JNEUROSCI.1925-13.2014, 24695716 PMC3972724

[ref65] Lörincz A. Notomi T. Tamás G. Shigemoto R. Nusser Z. (2002). Polarized and compartment-dependent distribution of HCN1 in pyramidal cell dendrites. Nat. Neurosci. 5, 1185–1193. doi: 10.1038/nn962, 12389030

[ref66] Lőrincz A. Nusser Z. (2010). Molecular identity of dendritic voltage-gated sodium channels. Science 328, 906–909. doi: 10.1126/science.1187958, 20466935 PMC3546315

[ref68] Losonczy A. Magee J. C. (2006). Integrative properties of radial oblique dendrites in hippocampal CA1 pyramidal neurons. Neuron 50, 291–307. doi: 10.1016/j.neuron.2006.03.016 16630839

[ref69] Magee J. C. (1998). Dendritic hyperpolarization-activated currents modify the integrative properties of hippocampal CA1 pyramidal neurons. J. Neurosci. 18, 7613–7624. doi: 10.1523/JNEUROSCI.18-19-07613.1998, 9742133 PMC6793032

[ref70] Magee J. C. Cook E. P. (2000). Somatic EPSP amplitude is independent of synapse location in hippocampal pyramidal neurons. Nat. Neurosci. 3, 895–903. doi: 10.1038/78800, 10966620

[ref71] Magee J. Hoffman D. Colbert C. Johnston D. (1998). Electrical and calcium signaling in dendrites of hippocampal pyramidal neurons. Annu. Rev. Physiol. 60, 327–346. doi: 10.1146/annurev.physiol.60.1.327, 9558467

[ref72] Major G. Polsky A. Denk W. Schiller J. Tank D. W. (2008). Spatiotemporally graded NMDA spike/plateau potentials in basal dendrites of neocortical pyramidal neurons. J. Neurophysiol. 99, 2584–2601. doi: 10.1152/jn.00011.2008, 18337370

[ref73] Markram H. Muller E. Ramaswamy S. Reimann M. W. Abdellah M. Sanchez C. A. . (2015). Reconstruction and simulation of neocortical microcircuitry. Cell 163, 456–492. doi: 10.1016/j.cell.2015.09.029 26451489

[ref74] Marrion N. V. Tavalin S. J. (1998). Selective activation of Ca2+−activated K+ channels by co-localized Ca2+ channels in hippocampal neurons. Nature 395, 900–905. doi: 10.1038/27674, 9804423

[ref75] Martina M. Jonas P. (1997). Functional differences in Na+ channel gating between fast-spiking interneurones and principal neurones of rat hippocampus. J. Physiol. 505, 593–603. doi: 10.1111/j.1469-7793.1997.593ba.x, 9457638 PMC1160038

[ref76] Matsuzaki M. Ellis-Davies G. C. R. Nemoto T. Miyashita Y. Iino M. Kasai H. (2001). Dendritic spine geometry is critical for AMPA receptor expression in hippocampal CA1 pyramidal neurons. Nat. Neurosci. 4, 1086–1092. doi: 10.1038/nn736, 11687814 PMC4229049

[ref77] Maurice N. Mercer J. Chan C. S. Hernandez-Lopez S. Held J. Tkatch T. . (2004). D2 dopamine receptor-mediated modulation of voltage-dependent Na+ channels reduces autonomous activity in striatal cholinergic interneurons. J. Neurosci. 24, 10289–10301. doi: 10.1523/JNEUROSCI.2155-04.2004, 15548642 PMC6730305

[ref78] McDermott C. M. Hardy M. N. Bazan N. G. Magee J. C. (2006). Sleep deprivation-induced alterations in excitatory synaptic transmission in the CA1 region of the rat hippocampus. J. Physiol. 570, 553–565. doi: 10.1113/jphysiol.2005.093781, 16322058 PMC1479879

[ref79] McKay B. E. McRory J. E. Molineux M. L. Hamid J. Snutch T. P. Zamponi G. W. . (2006). Cav3 t-type calcium channel isoforms differentially distribute to somatic and dendritic compartments in rat central neurons. Eur. J. Neurosci. 24, 2581–2594. doi: 10.1111/j.1460-9568.2006.05136.x, 17100846

[ref80] McNaughton N. C. L. Randall A. D. (1997). Electrophysiological properties of the human N-type Ca2+ channel: I. Channel gating in Ca2+, Ba2+ and Sr2+ containing solutions. Neuropharmacology 36, 895–915. doi: 10.1016/S0028-3908(97)00085-3, 9257935

[ref81] Megías M. Emri Z. Freund T. F. Gulyás A. I. (2001). Total number and distribution of inhibitory and excitatory synapses on hippocampal CA1 pyramidal cells. Neuroscience 102, 527–540. doi: 10.1016/S0306-4522(00)00496-6 11226691

[ref82] Metz A. E. Spruston N. Martina M. (2007). Dendritic D-type potassium currents inhibit the spike afterdepolarization in rat hippocampal CA1 pyramidal neurons. J. Physiol. 581, 175–187. doi: 10.1113/jphysiol.2006.127068, 17317746 PMC2075224

[ref83] Migliore M. Hoffman D. A. Magee J. C. Johnston D. (1999). Role of an A-type K+ conductance in the Back-propagation of action potentials in the dendrites of hippocampal pyramidal neurons. J. Comput. Neurosci. 7, 5–15. doi: 10.1023/A:1008906225285, 10481998

[ref84] Migliore R. Lupascu C. A. Bologna L. L. Romani A. Courcol J-D. Antonel S. . (2018). The physiological variability of channel density in hippocampal CA1 pyramidal cells and interneurons explored using a unified data-driven modeling workflow. PLoS Comput. Biol. 14:e1006423. doi: 10.1371/journal.pcbi.1006423 PMC6160220 30222740

[ref85] Misonou H. Mohapatra D. P. Trimmer J. S. (2005). Kv2.1: a voltage-gated k+ channel critical to dynamic control of neuronal excitability. Neurotoxicology 26, 743–752. doi: 10.1016/j.neuro.2005.02.003, 15950285

[ref86] Mitrovic N. George A. L. Jr. Horn R. (1998). Independent versus coupled inactivation in sodium channels: role of the domain 2 S4 segment. J. Gen. Physiol. 111, 451–462. 10.1085/jgp.111.3.451 PMC2217117 9482711

[ref87] Mohácsi M. Török M. P. Sáray S. Tar L. Farkas G. Káli S. (2024). Evaluation and comparison of methods for neuronal parameter optimization using the Neuroptimus software framework. PLoS Comput. Biol. 20:e1012039. doi: 10.1371/journal.pcbi.1012039, 39715260 PMC11706405

[ref88] Murphy J. G. Gutzmann J. J. Lin L. Hu J. Petralia R. S. Wang Y. X. . (2022). R-type voltage-gated Ca2+ channels mediate A-type K+ current regulation of synaptic input in hippocampal dendrites. Cell Rep. 38:110264. doi: 10.1016/j.celrep.2021.110264, 35045307 PMC10496648

[ref89] Otmakhova N. A. Otmakhov N. Lisman J. E. (2002). Pathway-specific properties of AMPA and NMDA-mediated transmission in CA1 hippocampal pyramidal cells. J. Neurosci. 22, 1199–1207. doi: 10.1523/JNEUROSCI.22-04-01199.2002, 11850447 PMC6757557

[ref90] Palmer L. M. Stuart G. J. (2006). Site of action potential initiation in layer 5 pyramidal neurons. J. Neurosci. 26, 1854–1863. doi: 10.1523/JNEUROSCI.4812-05.2006, 16467534 PMC6793621

[ref91] Palmer L. M. Stuart G. J. (2009). Membrane potential changes in dendritic spines during action potentials and synaptic input. J. Neurosci. 29, 6897–6903. doi: 10.1523/JNEUROSCI.5847-08.2009, 19474316 PMC6665597

[ref92] Pan A. C. Cuello L. G. Perozo E. Roux B. (2011). Thermodynamic coupling between activation and inactivation gating in potassium channels revealed by free energy molecular dynamics simulations. J. Gen. Physiol. 138, 571–580. doi: 10.1085/jgp.201110670, 22124115 PMC3226968

[ref93] Poirazi P. Brannon T. Mel B. W. (2003). Arithmetic of subthreshold synaptic summation in a model CA1 pyramidal cell. Neuron 37, 977–987. doi: 10.1016/S0896-6273(03)00148-X, 12670426

[ref94] Poolos N. P. Johnston D. (1999). Calcium-activated potassium Conductances contribute to action potential repolarization at the Soma but not the dendrites of hippocampal CA1 pyramidal neurons. J. Neurosci. 19, 5205–5212. doi: 10.1523/JNEUROSCI.19-13-05205.1999, 10377332 PMC6782335

[ref95] Randall A. D. Tsien R. W. (1997). Contrasting biophysical and pharmacological properties of T-type and R-type calcium channels. Neuropharmacology 36, 879–893. doi: 10.1016/S0028-3908(97)00086-5, 9257934

[ref96] Rapp M. Yarom Y. Segev I. (1992). The impact of parallel fiber background activity on the cable properties of cerebellar Purkinje cells. Neural Comput. 4, 518–533. doi: 10.1162/neco.1992.4.4.518

[ref97] Rasband M. N. (2010). The axon initial segment and the maintenance of neuronal polarity. Nat. Rev. Neurosci. 11, 552–562. doi: 10.1038/nrn2852, 20631711

[ref98] Reva M. Rössert C. Arnaudon A. Damart T. Mandge D. Tuncel A. . (2023). A universal workflow for creation, validation, and generalization of detailed neuronal models. Patterns 4:100855. doi: 10.1016/j.patter.2023.100855, 38035193 PMC10682753

[ref99] Romani A. Antonietti A. Bella D. Budd J. Giacalone E. Kurban K. . (2024). Community-based reconstruction and simulation of a full-scale model of the rat hippocampus CA1 region. PLoS Biol. 22:e3002861. doi: 10.1371/journal.pbio.3002861 PMC11537418 39499732

[ref100] Rudya B. McBain C. J. (2001). Kv3 channels: voltage-gated K+ channels designed for high-frequency repetitive firing. Trends Neurosci. 24, 517–526. doi: 10.1016/S0166-2236(00)01892-0, 11506885

[ref101] Sansom M. S. Ball F. G. Kerry C. J. McGee R. Ramsey R. L. Usherwood P. N. (1989). Markov, fractal, diffusion, and related models of ion channel gating. A comparison with experimental data from two ion channels. Biophys. J. 56, 1229–1243. doi: 10.1016/S0006-3495(89)82770-5, 2482085 PMC1280626

[ref102] Sáray S. Rössert C. A. Appukuttan S. Migliore R. Vitale P. Lupascu C. A. . (2021). HippoUnit: a software tool for the automated testing and systematic comparison of detailed models of hippocampal neurons based on electrophysiological data. PLoS Comput. Biol. 17, 1–38. doi: 10.1371/journal.pcbi.1008114, 33513130 PMC7875359

[ref103] Schneider C. J. Bezaire M. Soltesz I. (2012). Toward a full-scale computational model of the rat dentate gyrus. Front. Neural Circuits 6, 1–8. doi: 10.3389/fncir.2012.00083, 23162433 PMC3499761

[ref104] Schutter E. D. Bower J. M. (1994). An active membrane model of the cerebellar Purkinje cell. I. Simulation of current clamps in slice. J. Neurophysiol. 71, 375–400. doi: 10.1152/jn.1994.71.1.375, 7512629

[ref105] Shah M. M. Migliore M. Brown D. A. (2011). Differential effects of Kv7 (M-) channels on synaptic integration in distinct subcellular compartments of rat hippocampal pyramidal neurons. J. Phys. 589, 6029–6038. doi: 10.1113/jphysiol.2011.220913, 22041186 PMC3245855

[ref106] Siekmann I. Fackrell M. Crampin E. J. Taylor P. (2016). Modelling modal gating of ion channels with hierarchical Markov models. Proceedings Mathematical Physical Engineering Sciences 472:20160122. doi: 10.1098/rspa.2016.0122, 27616917 PMC5014102

[ref107] Sivagnanam S. Majumdar A. Yoshimoto K. Astakhov V. Bandrowski A. Martone ME . Introducing the Neuroscience Gateway CEUR Workshop Proceedings 2013. 993

[ref108] Siwiec M. Kusek M. Sowa J. E. Tokarski K. Hess G. (2020). 5-HT7 receptors increase the excitability of hippocampal CA1 pyramidal neurons by inhibiting the A-type potassium current. Neuropharmacology 177:108248. doi: 10.1016/j.neuropharm.2020.108248, 32736087

[ref109] Spruston N. (2008). Pyramidal neurons: dendritic structure and synaptic integration. Nat. Rev. Neurosci. 9, 206–221. doi: 10.1038/nrn2286, 18270515

[ref110] Stocker M. (2004). Ca2+−activated K+ channels: molecular determinants and function of the SK family. Nat. Rev. Neurosci. 5, 758–770. doi: 10.1038/nrn1516, 15378036

[ref111] Storm J. F. (1988). Temporal integration by a slowly inactivating K+ current in hippocampal neurons. Nature 336:698. doi: 10.1038/336698b0, 3194020

[ref112] Storm J. F. (1990). Potassium currents in hippocampal pyramidal cells. Prog. Brain Res. 83, 161–187. doi: 10.1016/s0079-6123(08)61248-0, 2203097

[ref113] Stuart G. J. Sakmann B. (1994). Active propagation of somatic action potentials into neocortical pyramidal cell dendrites. Nature 367, 69–72. doi: 10.1038/367069a0, 8107777

[ref114] Takács V. Bardóczi Z. Orosz Á. Major A. Tar L. Berki P. . (2024). Synaptic and dendritic architecture of different types of hippocampal somatostatin interneurons. PLoS Biol. 22:e3002539. doi: 10.1371/journal.pbio.3002539, 38470935 PMC10959371

[ref115] Terbe D. Szoboszlay M. Nusser Z. Káli S. (2022). Reliable estimation of neuronal biophysical parameters from whole-cell recordings using model simulations and probabilistic inference Program No. 524.10. 2022 Neuroscience Meeting Planner. San Diego, CA: Society for Neuroscience. Available online at: https://www.abstractsonline.com/pp8/#!/10619/presentation/83939

[ref116] Tomko M. Benuskova L. Jedlicka P. (2021). A new reduced-morphology model for CA1 pyramidal cells and its validation and comparison with other models using HippoUnit. Sci. Rep. 11:7615. doi: 10.1038/s41598-021-87002-7, 33828151 PMC8027802

[ref117] Tønnesen J. Nägerl U. V. (2016). Dendritic spines as tunable regulators of synaptic signals. Front. Psych. 7:101. doi: 10.3389/fpsyt.2016.00101 PMC4899469 27340393

[ref118] Traub R. D. Contreras D. Cunningham M. O. Murray H. LeBeau F. E. N. Roopun A. . (2005). Single-column thalamocortical network model exhibiting gamma oscillations, sleep spindles, and epileptogenic bursts. J. Neurophysiol. 93, 2194–2232. doi: 10.1152/jn.00983.2004, 15525801

[ref119] Traub R. D. Wong R. K. Miles R. Michelson H. (1991). A model of a CA3 hippocampal pyramidal neuron incorporating voltage-clamp data on intrinsic conductances. J. Physiol. 66, 635–650. doi: 10.1152/jn.1991.66.2.635, 1663538

[ref120] Tsay D. Yuste R. (2004). On the electrical function of dendritic spines. Trends Neurosci. 27, 77–83. doi: 10.1016/j.tins.2003.11.008, 15102486

[ref121] Ujfalussy B. B. Makara J. K. (2020). Impact of functional synapse clusters on neuronal response selectivity. Nat. Commun. 11:1413. doi: 10.1038/s41467-020-15147-6, 32179739 PMC7075899

[ref31] Van Geit W. Gevaert M. Chindemi G. Rössert C. Courcol J. D. Muller E. B. . (2016). BluePyOpt: leveraging open source software and cloud infrastructure to optimise model parameters in neuroscience. Front. Neuroinform. 10:17. doi: 10.3389/fninf.2016.00017 PMC4896051 27375471

[ref4] Varga A. W. Yuan L-L. Anderson A. E. Schrader L. A. Wu G. Y. Gatchel J. R. . (2004). Calcium–calmodulin-dependent kinase II modulates Kv4.2 channel expression and upregulates neuronal A-type potassium currents. J. Neurosci. 24, 3643–3654. doi: 10.1523/JNEUROSCI.0154-04.2004 PMC6729731 15071113

[ref122] Victoria N. C. Velasco E. M. F. d. Ostrovskaya O. Metzger S. Xia Z. Kotecki L. . (2016). G protein-gated K+ channel ablation in forebrain pyramidal neurons selectively impairs fear learning. Biol. Psychiatry 80, 796–806. doi: 10.1016/j.biopsych.2015.10.004, 26612516 PMC4862939

[ref123] Vogalis F. Storm J. F. Lancaster B. (2003). SK channels and the varieties of slow after-hyperpolarizations in neurons. Eur. J. Neurosci. 18, 3155–3166. doi: 10.1111/j.1460-9568.2003.03040.x, 14686890

[ref124] Yasuda R. Sabatini B. L. Svoboda K. (2003). Plasticity of calcium channels in dendritic spines. Nat. Neurosci. 6, 948–955. doi: 10.1038/nn1112, 12937422

[ref125] Yuste R. (2011). Dendritic spines and distributed circuits. Neuron 71, 772–781. doi: 10.1016/j.neuron.2011.07.024, 21903072 PMC4071954

[ref126] Yuste R. (2013). Electrical compartmentalization in dendritic spines. Annu. Rev. Neurosci. 36, 429–449. doi: 10.1146/annurev-neuro-062111-150455, 23724997

[ref127] Zemankovics R. Káli S. Paulsen O. Freund T. F. Hájos N. (2010). Differences in subthreshold resonance of hippocampal pyramidal cells and interneurons: the role of h-current and passive membrane characteristics. J. Physiol. 588, 2109–2132. doi: 10.1113/jphysiol.2009.185975, 20421280 PMC2905616

[ref128] Zhang Y. He G. Ma L. Liu X. Hjorth J. J. J. Kozlov A. . (2023). A GPU-based computational framework that bridges neuron simulation and artificial intelligence. Nat. Commun. 14:5798. doi: 10.1038/s41467-023-41553-7, 37723170 PMC10507119

