# Supplementary material for: How to train your neuron: developing a detailed, up-to-date, multipurpose model of hippocampal CA1 pyramidal cells
Source: Front Comput Neurosci. 2026 Sep 22;20:1897214. doi: 10.3389/fncom.2026.1897214 (PMC13638379; doi:10.3389/fncom.2026.1897214)
Supplement: Supplementary file 1 [file Data_Sheet_1.pdf]

Supplementary information for Tar et al., „How to train your neuron: Developing a detailed, up-to-date, multipurpose model of hippocampal CA1 pyramidal cells"

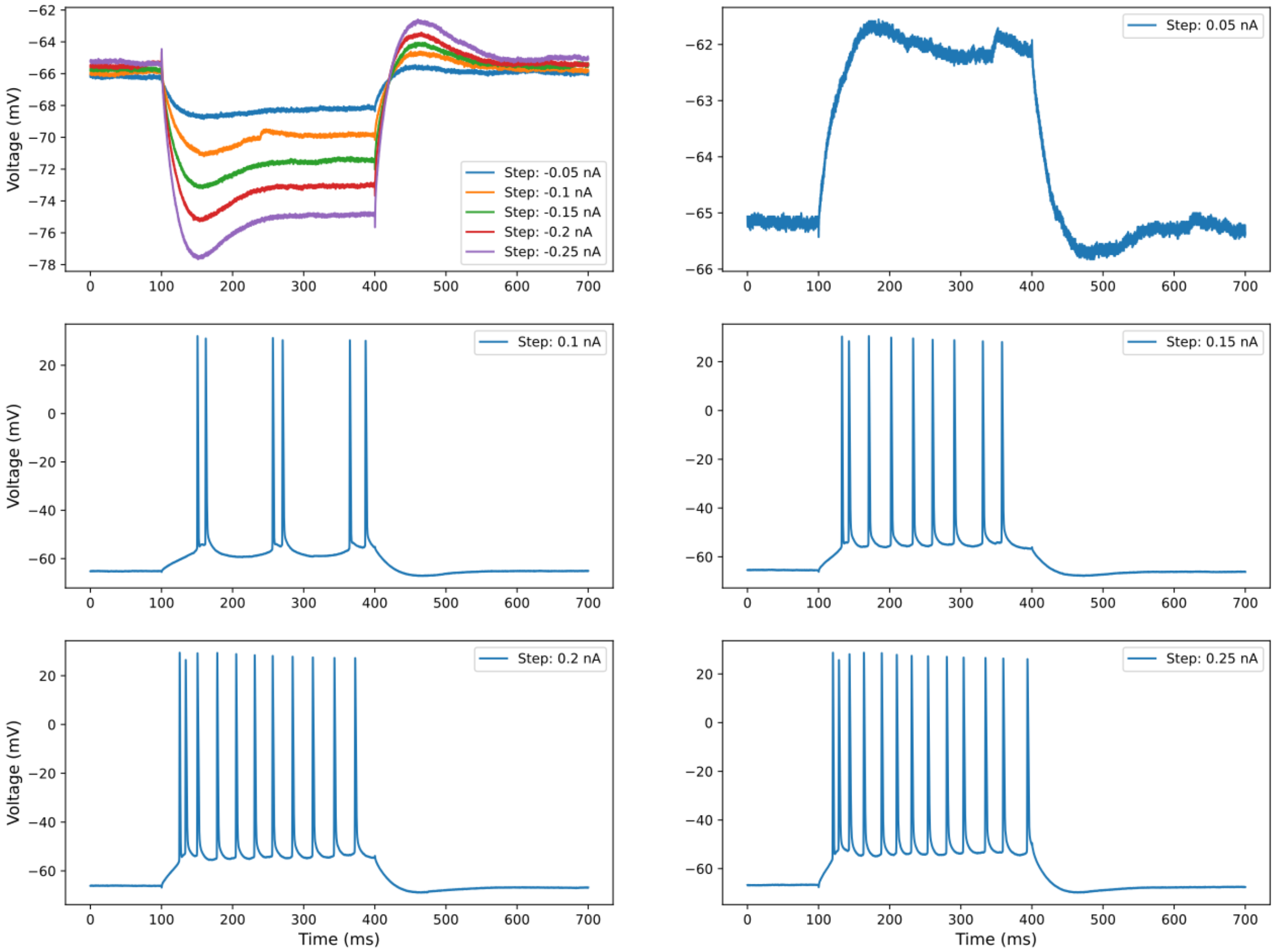

Supplementary Figure 1: Experimental voltage traces from our standard current step protocol in the cell that was excluded from the feature extraction. This cell (unlike others) had a clear tendency to fire spike doublets or brief bursts at the beginning of the current step.

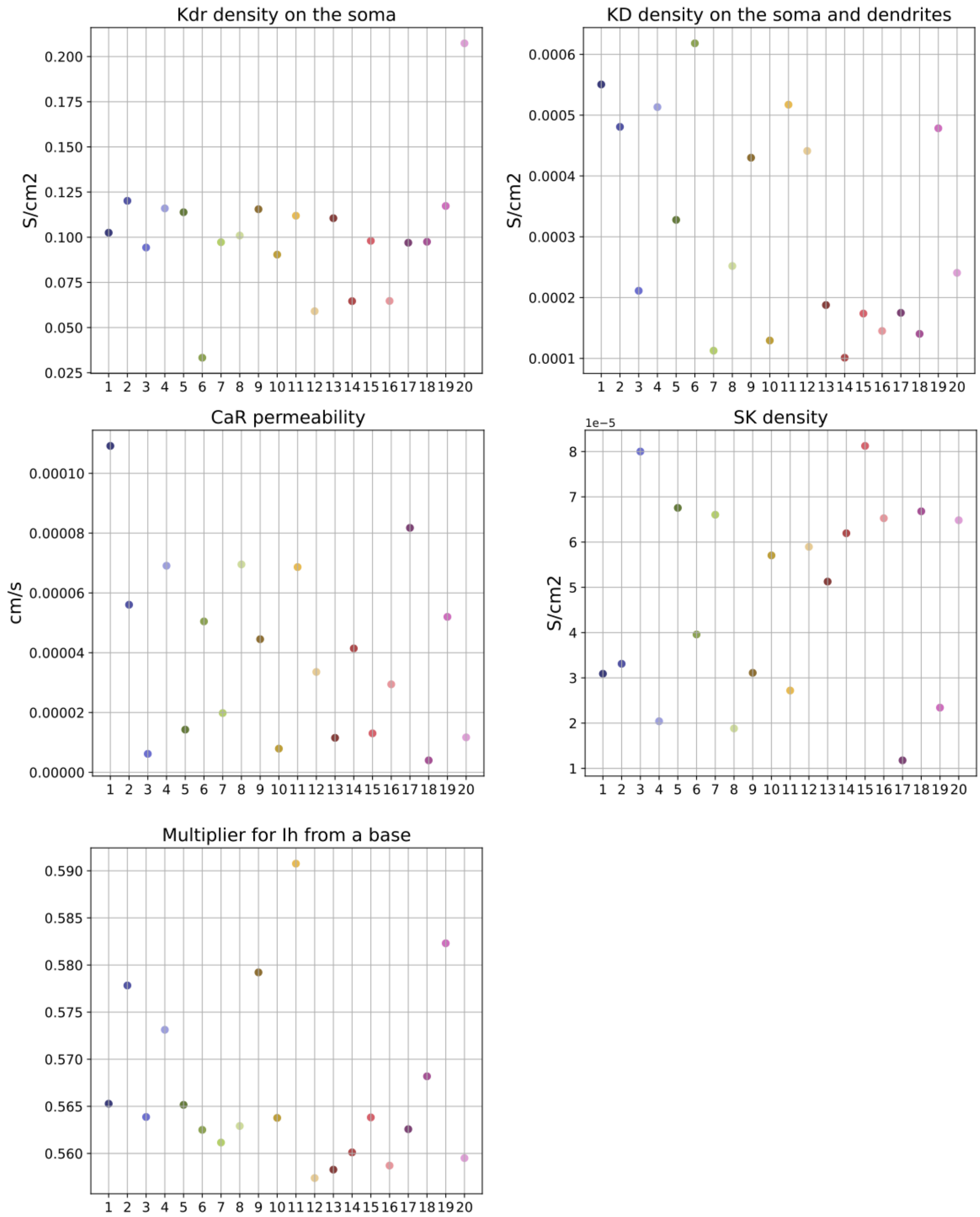

Supplementary Figure 2: A magnified view of five parameters from Figure 2 without their bounds. The x-axis and the different colors represent the individual models by their numbers, while on the y-axis there are the parameter values for each model and parameter.

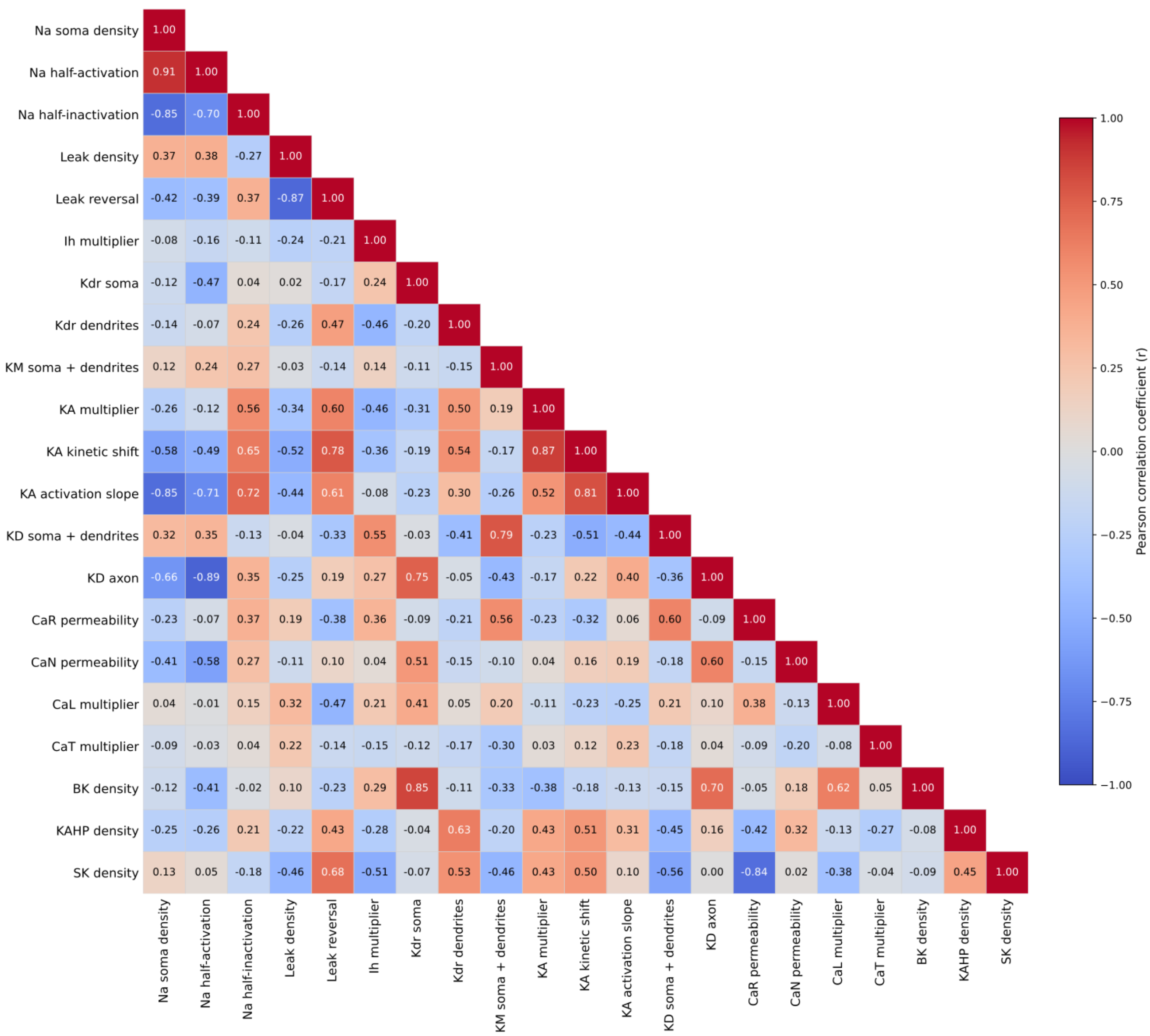

Supplementary Figure 3: Pairwise correlation coefficients for the 21 optimized parameters. We calculated the Pearson correlation coefficient for each parameter pair and represented them on a heatmap. Red indicates positive correlation between the pairs while blue indicates negative correlations. The exact value of the Pearson correlation coefficients is displayed in each cell.

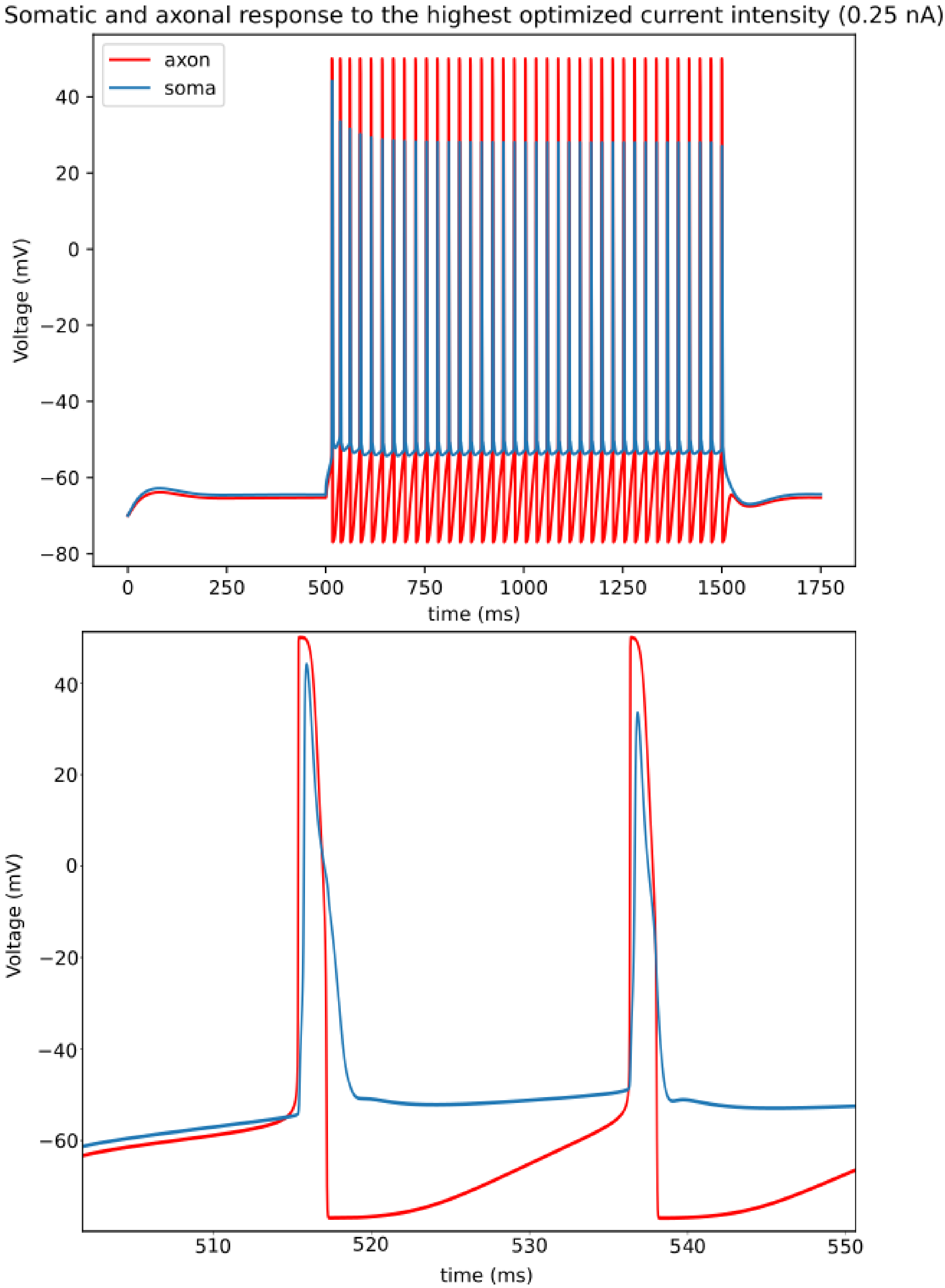

Supplementary Figure 4: Voltage response of one of the optimized models at the soma (blue) and in the axon (red) to a 0.25 nA current step input to the soma. The bottom figure shows a close-up of the first two spikes, proving that the spike is indeed initiated in the axon.

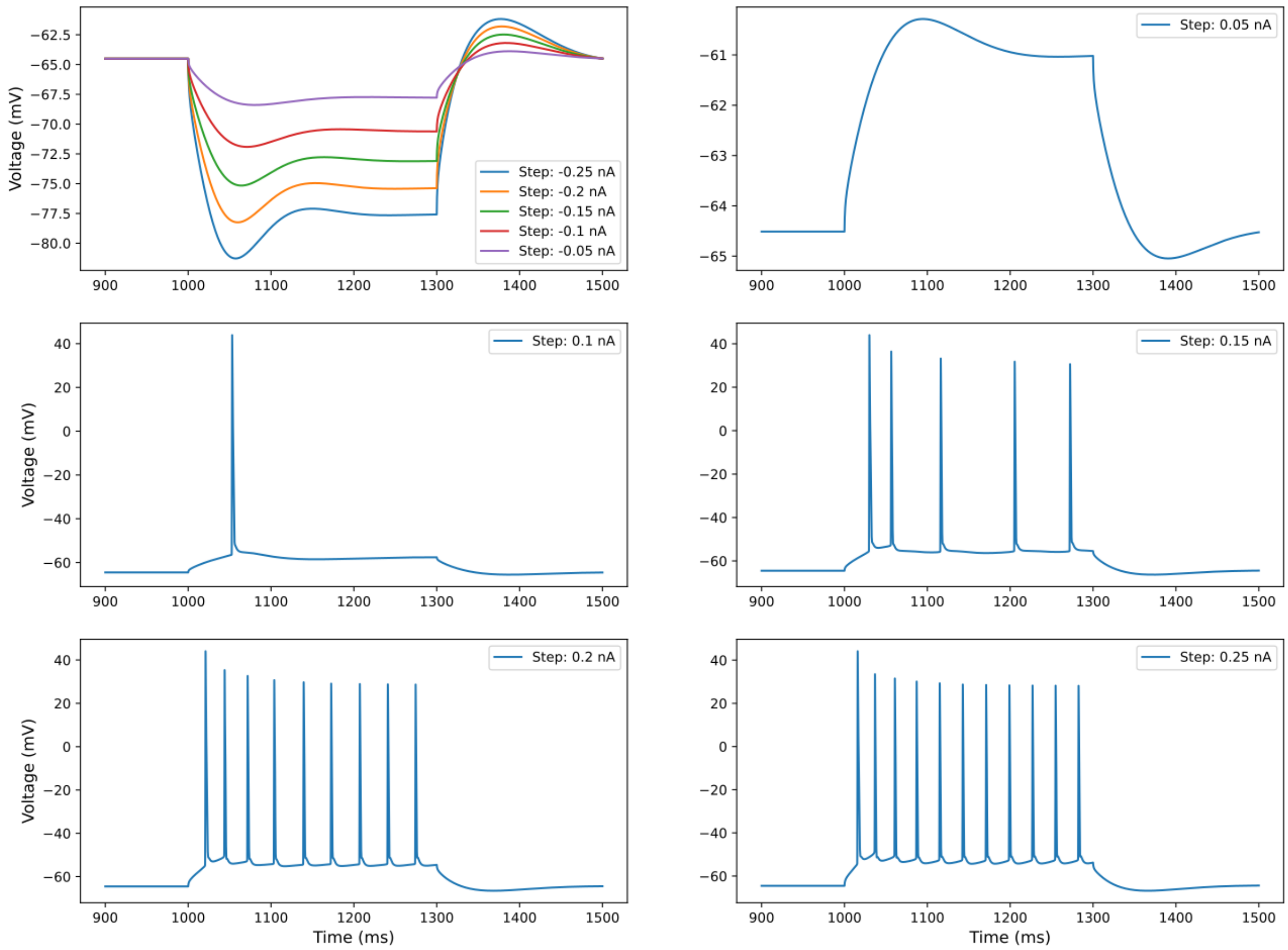

Supplementary Figure 5: Example of the models' response to the current step protocol. This protocol was used for parameter fitting and for validation of somatic features, and the resulting traces served as the basis for the extraction of the eFEL features. The long delay was used during the Somatic Features validation to stabilize the resting membrane potential.

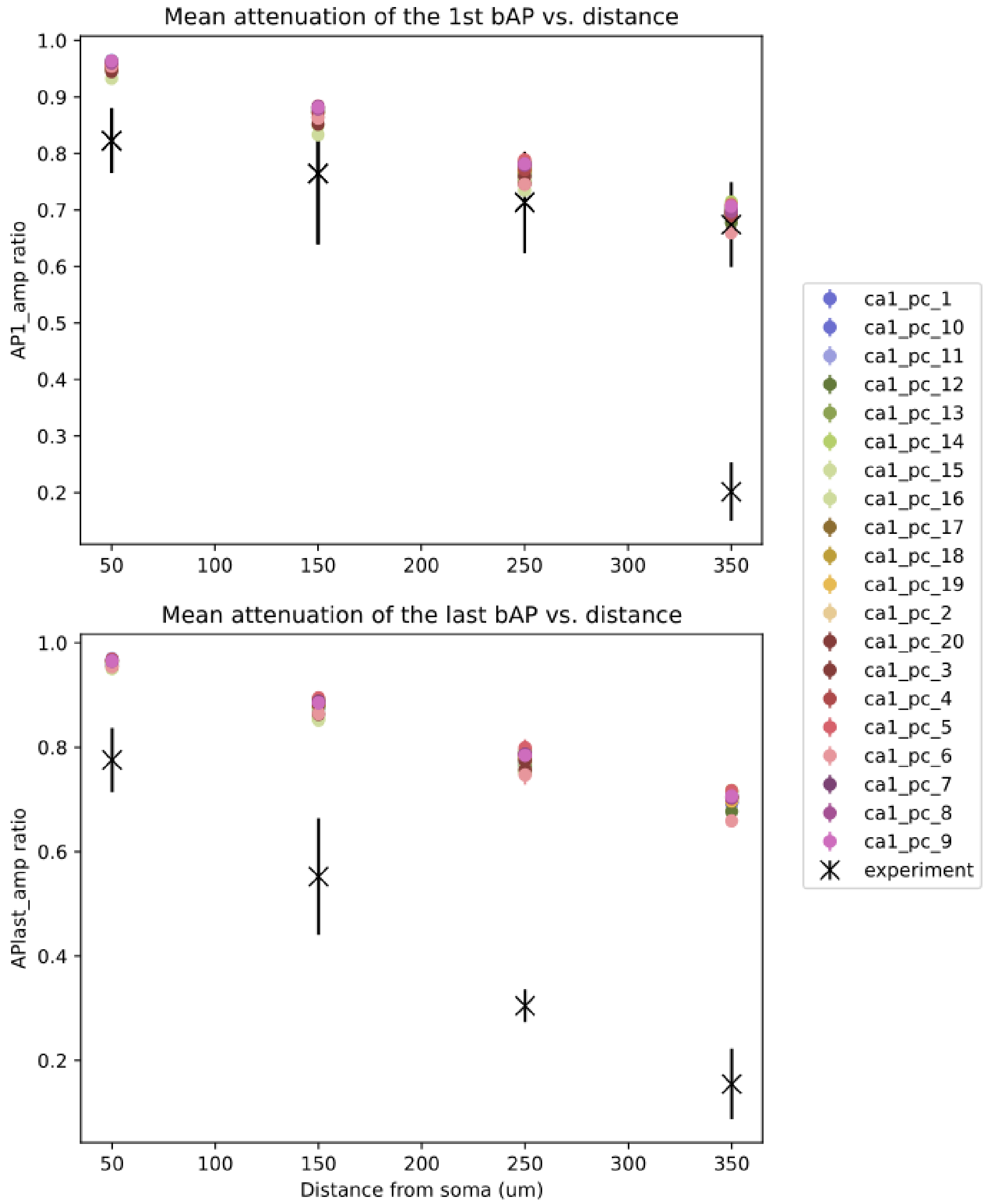

Supplementary Figure 6: Removing the slow inactivation of dendritic sodium channels prevents the shrinking of the spikes during the action potential train in the Backpropagating Action Potential Test.

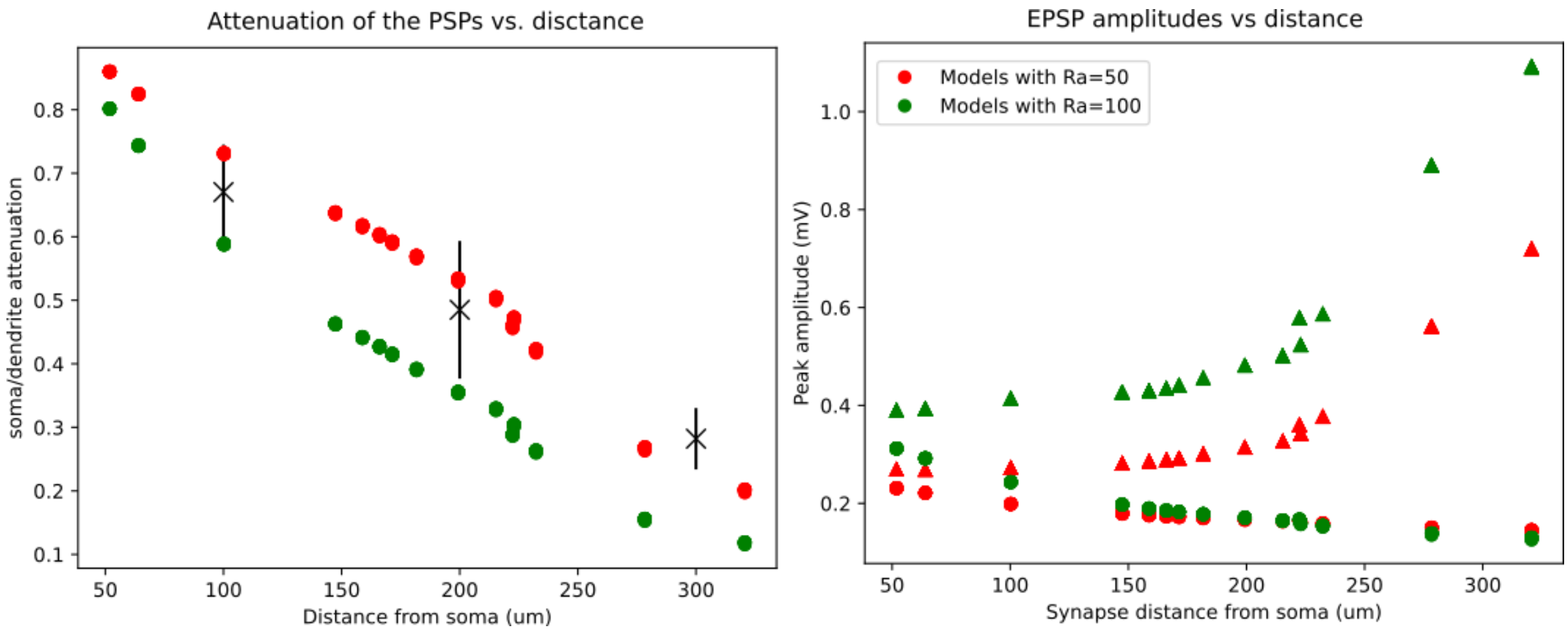

Supplementary Figure 7: Comparison of the results of the PSP Attenuation Test when the axial resistance (Ra) of the model is 50 Ohm×cm (red) versus 100 Ohm×cm (green).

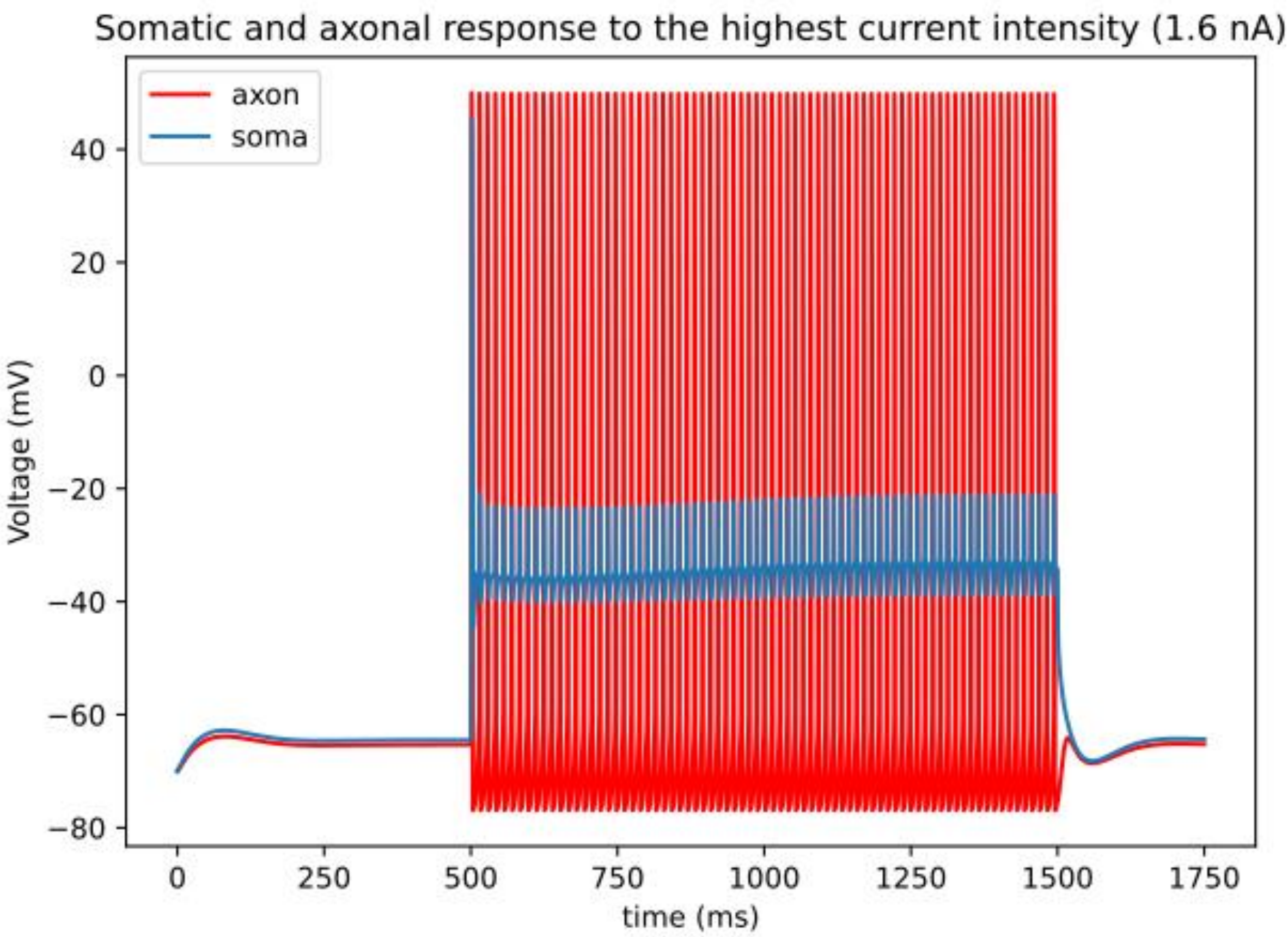

Supplementary Figure 8: Voltage response of an optimized model to a 1.6 nA current step, which is used in the depolarization block test. Blue is the response on the soma while the axon can be seen in red, proving that the spikelets that can be seen in the soma originate from the axon.

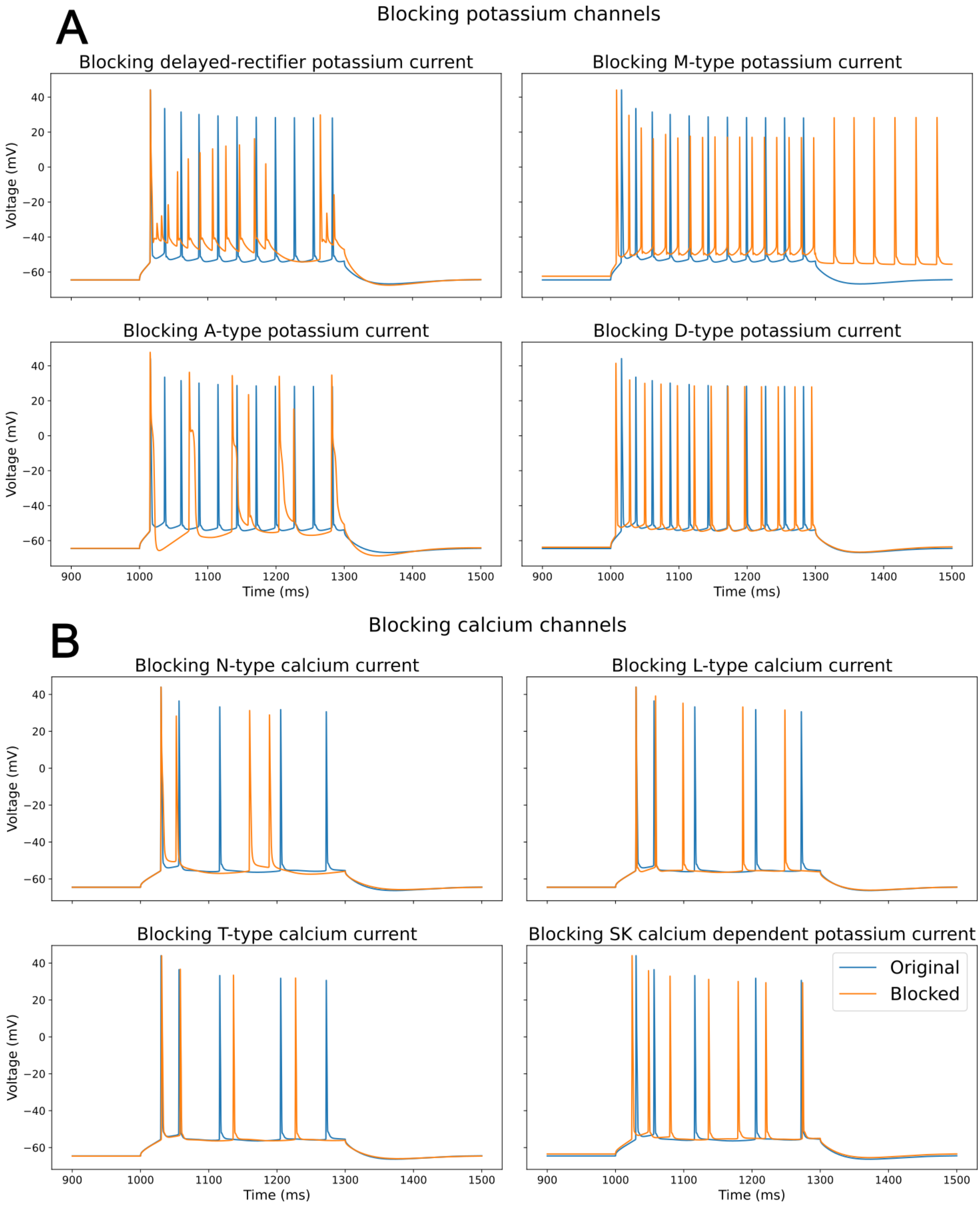

Supplementary Figure 9: Blocking different ion channels to check if their effect on the model is the same as the effect reported in the experimental literature. (A) shows traces resulting from blocking four different potassium channels along with the original voltage traces for a 0.25 nA current injection. (B) shows the results of blocking three calcium channels and the small-conductance calcium-dependent potassium channel for a current amplitude of 0.15 nA. The reason for the different current amplitudes is to emphasize the differences between the original and blocked traces.

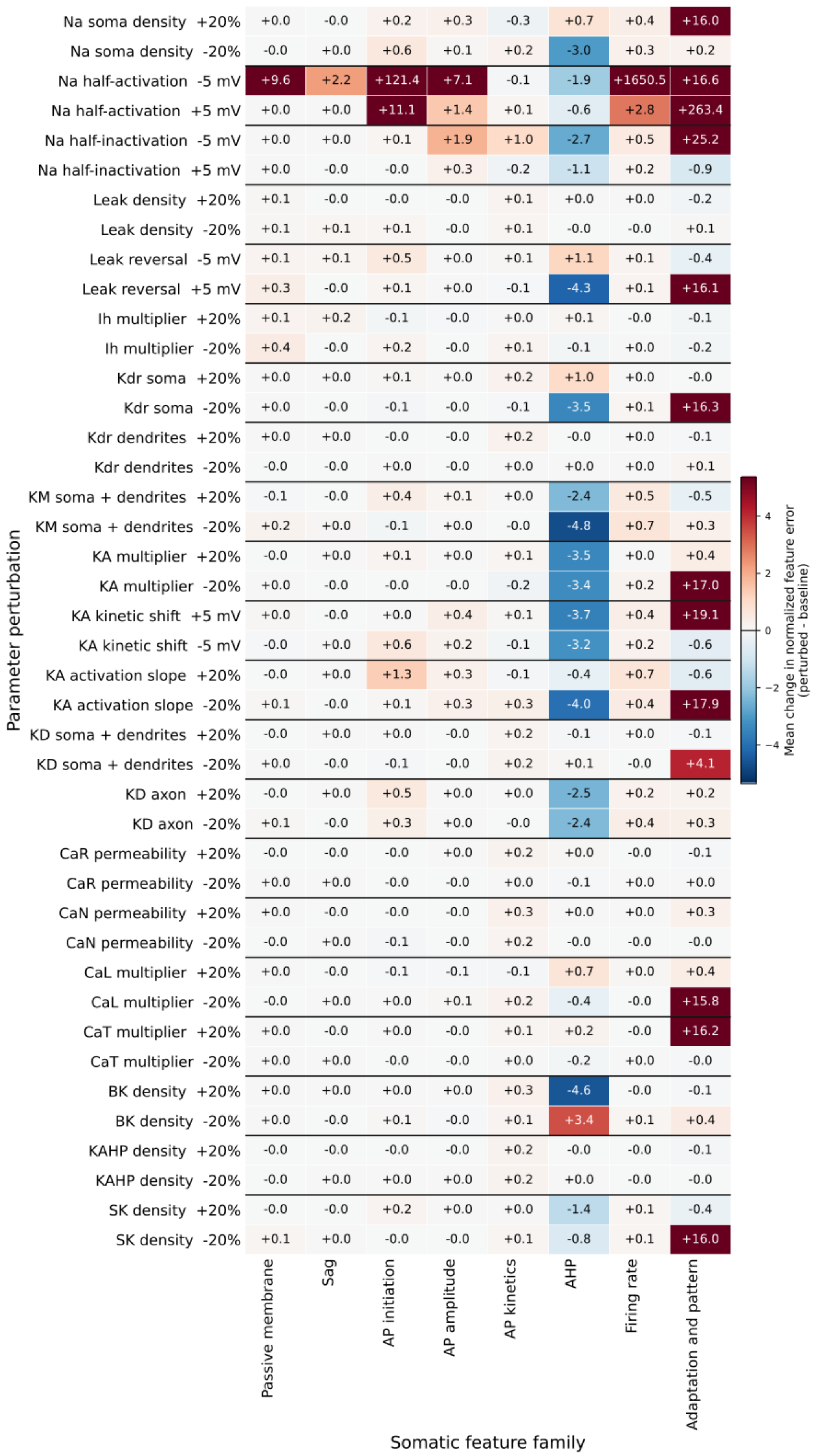

Parameter perturbation
Somatic feature family
Passive membrane
Sag
AP initiation
AP amplitude
AP kinetics
AHP
Firing rate
Adaptation and pattern
Na soma density +20% +0.0 -0.0 +0.2 +0.3 -0.3 +0.7 +0.4 +16.0
Na soma density -20% -0.0 +0.0 +0.6 +0.1 +0.2 -3.0 +0.3 +0.2
Na half-activation -5 mV +9.6 +2.2 +121.4 +7.1 -0.1 -1.9 +1650.5 +16.6
Na half-activation +5 mV +0.0 +0.0 +11.1 +1.4 +0.1 -0.6 +2.8 +263.4
Na half-inactivation -5 mV +0.0 +0.0 +0.1 +1.9 +1.0 -2.7 +0.5 +25.2
Na half-inactivation +5 mV +0.0 -0.0 -0.0 +0.3 -0.2 -1.1 +0.2 -0.9
Leak density +20% +0.1 -0.0 -0.0 -0.0 +0.1 +0.0 +0.0 -0.2
Leak density -20% +0.1 +0.1 +0.1 -0.0 +0.1 -0.0 -0.0 +0.1
Leak reversal -5 mV +0.1 +0.1 +0.5 +0.0 +0.1 +1.1 +0.1 -0.4
Leak reversal +5 mV +0.3 -0.0 +0.1 +0.0 -0.1 -4.3 +0.1 +16.1
Ih multiplier +20% +0.1 +0.2 -0.1 -0.0 +0.0 +0.1 -0.0 -0.1
Ih multiplier -20% +0.4 -0.0 +0.2 -0.0 +0.1 -0.1 +0.0 -0.2
Kdr soma +20% +0.0 +0.0 +0.1 +0.0 +0.2 +1.0 +0.0 -0.0
Kdr soma -20% +0.0 -0.0 -0.1 -0.0 -0.1 -3.5 +0.1 +16.3
Kdr dendrites +20% +0.0 +0.0 -0.0 -0.0 +0.2 -0.0 +0.0 -0.1
Kdr dendrites -20% -0.0 -0.0 +0.0 -0.0 +0.0 +0.0 +0.0 +0.1
KM soma + dendrites +20% -0.1 -0.0 +0.4 +0.1 +0.0 -2.4 +0.5 -0.5
KM soma + dendrites -20% +0.2 +0.0 -0.1 +0.0 -0.0 -4.8 +0.7 +0.3
KA multiplier +20% -0.0 +0.0 +0.1 +0.0 +0.1 -3.5 +0.0 +0.4
KA multiplier -20% +0.0 -0.0 -0.0 -0.0 -0.2 -3.4 +0.2 +17.0
KA kinetic shift +5 mV +0.0 -0.0 +0.0 +0.4 +0.1 -3.7 +0.4 +19.1
KA kinetic shift -5 mV -0.0 +0.0 +0.6 +0.2 -0.1 -3.2 +0.2 -0.6
KA activation slope +20% -0.0 +0.0 +1.3 +0.3 -0.1 -0.4 +0.7 -0.6
KA activation slope -20% +0.1 -0.0 +0.1 +0.3 +0.3 -4.0 +0.4 +17.9
KD soma + dendrites +20% -0.0 +0.0 +0.0 -0.0 +0.2 -0.1 +0.0 -0.1
KD soma + dendrites -20% +0.0 -0.0 -0.1 -0.0 +0.2 +0.1 -0.0 +4.1
KD axon +20% -0.0 +0.0 +0.5 +0.0 +0.0 -2.5 +0.2 +0.2
KD axon -20% +0.1 -0.0 +0.3 +0.0 -0.0 -2.4 +0.4 +0.3
CaR permeability +20% -0.0 -0.0 -0.0 +0.0 +0.2 +0.0 -0.0 -0.1
CaR permeability -20% +0.0 +0.0 -0.0 -0.0 +0.0 -0.1 +0.0 +0.0
CaN permeability +20% +0.0 -0.0 -0.0 -0.0 +0.3 +0.0 +0.0 +0.3
CaN permeability -20% -0.0 +0.0 -0.1 -0.0 +0.2 -0.0 -0.0 -0.0
CaL multiplier +20% +0.0 -0.0 -0.1 -0.1 -0.1 +0.7 +0.0 +0.4
CaL multiplier -20% -0.0 +0.0 +0.0 +0.1 +0.2 -0.4 -0.0 +15.8
CaT multiplier +20% +0.0 -0.0 +0.0 -0.0 +0.1 +0.2 -0.0 +16.2
CaT multiplier -20% +0.0 +0.0 -0.0 -0.0 +0.0 -0.2 +0.0 -0.0
BK density +20% +0.0 +0.0 +0.0 +0.0 +0.3 -4.6 -0.0 -0.1
BK density -20% +0.0 -0.0 +0.1 -0.0 +0.1 +3.4 +0.1 +0.4
KAHP density +20% -0.0 -0.0 -0.0 -0.0 +0.2 -0.0 -0.0 -0.1
KAHP density -20% -0.0 +0.0 +0.0 +0.0 +0.2 +0.0 -0.0 -0.0
SK density +20% -0.0 -0.0 +0.2 +0.0 +0.0 -1.4 +0.1 -0.4
SK density -20% +0.1 +0.0 -0.0 -0.0 +0.1 -0.8 +0.1 +16.0
4
2
0
−2
−4
Mean change in normalized feature error
(perturbed - baseline)

Supplementary Figure 10: Sensitivity of feature errors of the Somatic Features Test of HippoUnit to variations in model parameters. We chose a model parameter set (ca1_pc_13) and performed a sensitivity analysis for each HippoUnit validation test. We systematically increased and decreased individual parameter values by 20%, except for the parameters that represent membrane potential values, which were changed by ± 5 mV. The change in the feature errors was calculated as Δerror = Error after perturbation − baseline error. To enable the representation of the large number of features in the Somatic Features Test in a single figure, we first calculated the mean of the error changes across the different current step amplitudes for each feature, then we arranged the features into feature families. The error change for each feature family is the mean error change of the features in the given family. The resulting changes are shown as a heat map. We clipped the color scale at +5 to make both negative and positive changes visible, and also because large positive errors typically result from penalties added by HippoUnit when the behavior of the model is qualitatively different from the experimentally observed physiological behavior. Examples of this can be seen in the rows corresponding to the perturbation of the half-activation of sodium channels where HippoUnit assigned penalties to models that are spontaneously firing or not firing at all.

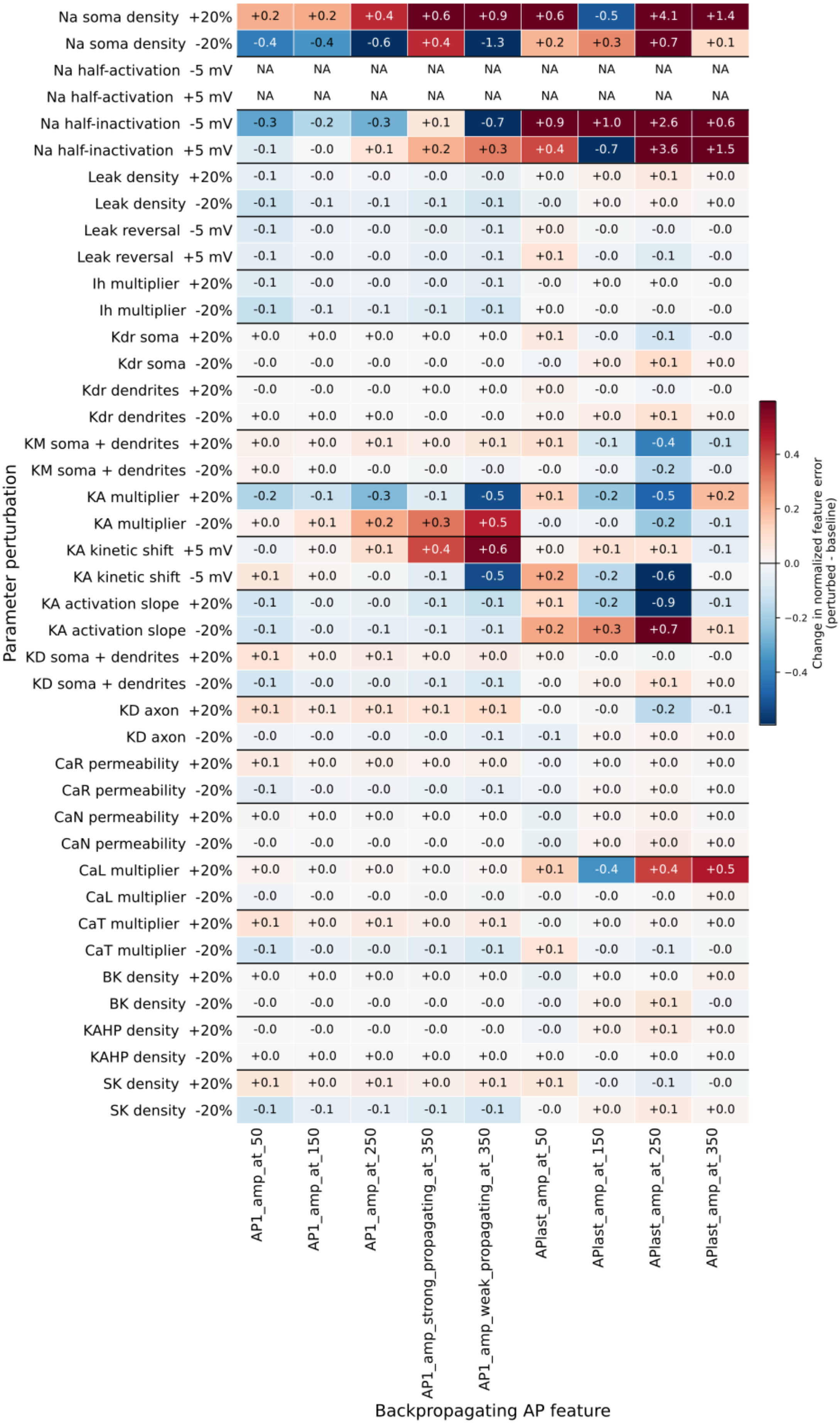
Parameter perturbation
Backpropagating AP feature
AP1_amp_at_50 AP1_amp_at_150 AP1_amp_at_250 AP1_amp_strong_propagating_at_350 AP1_amp_weak_propagating_at_350 APlast_amp_at_50 APlast_amp_at_150 APlast_amp_at_250 APlast_amp_at_350
Na soma density +20% +0.2 +0.2 +0.4 +0.6 +0.9 +0.6 -0.5 +4.1 +1.4
Na soma density -20% -0.4 -0.4 -0.6 +0.4 -1.3 +0.2 +0.3 +0.7 +0.1
Na half-activation -5 mV NA NA NA NA NA NA NA NA NA
Na half-activation +5 mV NA NA NA NA NA NA NA NA NA
Na half-inactivation -5 mV -0.3 -0.2 -0.3 +0.1 -0.7 +0.9 +1.0 +2.6 +0.6
Na half-inactivation +5 mV -0.1 -0.0 +0.1 +0.2 +0.3 +0.4 -0.7 +3.6 +1.5
Leak density +20% -0.1 -0.0 -0.0 -0.0 -0.0 +0.0 +0.0 +0.1 +0.0
Leak density -20% -0.1 -0.1 -0.1 -0.1 -0.1 -0.0 +0.0 +0.0 +0.0
Leak reversal -5 mV -0.1 -0.0 -0.0 -0.0 -0.1 +0.0 -0.0 -0.0 -0.0
Leak reversal +5 mV -0.1 -0.0 -0.0 -0.0 -0.1 +0.1 -0.0 -0.1 -0.0
Ih multiplier +20% -0.1 -0.0 -0.0 -0.0 -0.1 -0.0 +0.0 +0.0 -0.0
Ih multiplier -20% -0.1 -0.1 -0.1 -0.1 -0.1 +0.0 -0.0 -0.0 -0.0
Kdr soma +20% +0.0 +0.0 +0.0 +0.0 +0.0 +0.1 -0.0 -0.1 -0.0
Kdr soma -20% -0.0 -0.0 -0.0 -0.0 -0.0 -0.0 +0.0 +0.1 +0.0
Kdr dendrites +20% -0.0 -0.0 -0.0 +0.0 +0.0 +0.0 -0.0 -0.0 -0.0
Kdr dendrites -20% +0.0 +0.0 +0.0 -0.0 -0.0 +0.0 +0.0 +0.1 +0.0
KM soma + dendrites +20% +0.0 +0.0 +0.1 +0.0 +0.1 +0.1 -0.1 -0.4 -0.1
KM soma + dendrites -20% +0.0 +0.0 -0.0 -0.0 -0.0 -0.0 -0.0 -0.2 -0.0
KA multiplier +20% -0.2 -0.1 -0.3 -0.1 -0.5 +0.1 -0.2 -0.5 +0.2
KA multiplier -20% +0.0 +0.1 +0.2 +0.3 +0.5 -0.0 -0.0 -0.2 -0.1
KA kinetic shift +5 mV -0.0 +0.0 +0.1 +0.4 +0.6 +0.0 +0.1 +0.1 -0.1
KA kinetic shift -5 mV +0.1 +0.0 -0.0 -0.1 -0.5 +0.2 -0.2 -0.6 -0.0
KA activation slope +20% -0.1 -0.0 -0.0 -0.1 -0.1 +0.1 -0.2 -0.9 -0.1
KA activation slope -20% -0.1 -0.0 -0.1 -0.1 -0.1 +0.2 +0.3 +0.7 +0.1
KD soma + dendrites +20% +0.1 +0.0 +0.1 +0.0 +0.0 +0.0 -0.0 -0.0 -0.0
KD soma + dendrites -20% -0.1 -0.0 -0.0 -0.1 -0.1 -0.0 +0.0 +0.1 +0.0
KD axon +20% +0.1 +0.1 +0.1 +0.1 +0.1 -0.0 -0.0 -0.2 -0.1
KD axon -20% -0.0 -0.0 -0.0 -0.0 -0.1 -0.1 +0.0 +0.0 +0.0
CaR permeability +20% +0.1 +0.0 +0.0 +0.0 +0.0 -0.0 +0.0 +0.0 +0.0
CaR permeability -20% -0.1 -0.0 -0.0 -0.0 -0.1 -0.0 +0.0 +0.0 +0.0
CaN permeability +20% +0.0 +0.0 +0.0 +0.0 +0.0 -0.0 +0.0 +0.0 +0.0
CaN permeability -20% -0.0 -0.0 -0.0 -0.0 -0.0 -0.0 +0.0 +0.0 +0.0
CaL multiplier +20% +0.0 +0.0 +0.0 +0.0 +0.0 +0.1 -0.4 +0.4 +0.5
CaL multiplier -20% -0.0 -0.0 -0.0 -0.0 -0.0 -0.0 -0.0 -0.0 +0.0
CaT multiplier +20% +0.1 +0.0 +0.1 +0.0 +0.1 -0.0 +0.0 +0.0 +0.0
CaT multiplier -20% -0.1 -0.0 -0.0 -0.1 -0.1 +0.1 -0.0 -0.1 -0.0
BK density +20% +0.0 +0.0 +0.0 +0.0 +0.0 -0.0 +0.0 +0.0 +0.0
BK density -20% -0.0 -0.0 -0.0 -0.0 -0.0 -0.0 +0.0 +0.1 -0.0
KAHP density +20% -0.0 -0.0 -0.0 -0.0 -0.0 -0.0 +0.0 +0.1 +0.0
KAHP density -20% +0.0 +0.0 +0.0 +0.0 +0.0 +0.0 -0.0 +0.0 +0.0
SK density +20% +0.1 +0.0 +0.1 +0.0 +0.1 +0.1 -0.0 -0.1 -0.0
SK density -20% -0.1 -0.1 -0.1 -0.1 -0.1 -0.0 +0.0 +0.1 +0.0
Change in normalized feature error
(perturbed - baseline)
0.4
0.2
0.0
−0.2
−0.4

Supplementary Figure 11: Sensitivity of feature errors of the Back-propagating Action Potential Test of HippoUnit to variations in model parameters. This figure is analogous to Supplementary Figure 10, except that we clipped the color scale at -0.5 and +0.5 in this case. When we varied the half-activation of the sodium channels (rows 3 and 4) the test could not find a current injection amplitude where the model fires at 15 Hz. Perturbating this parameter led to a spontaneously firing cell model (-5 mV change) or the model firing only at very high current amplitudes (+5 mV change).

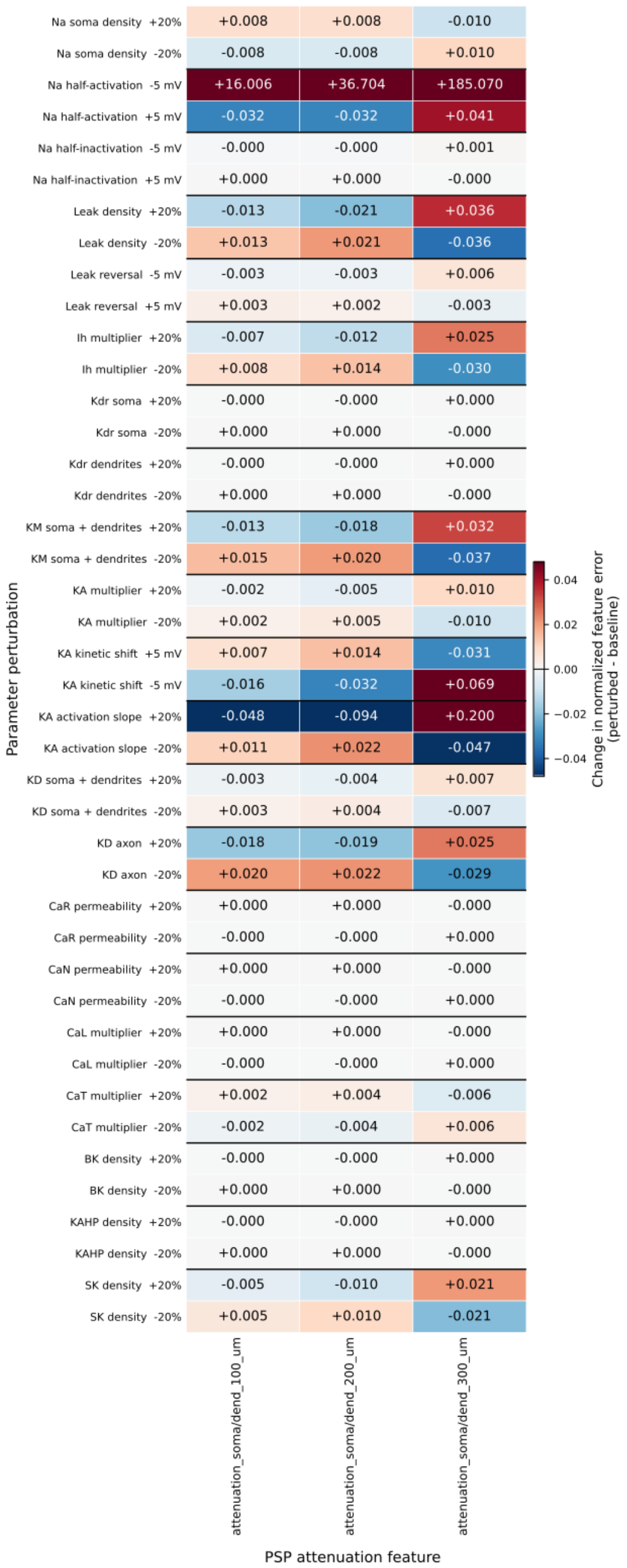
Parameter perturbation
Na soma density +20% +0.008 +0.008 -0.010
Na soma density -20% -0.008 -0.008 +0.010
Na half-activation -5 mV +16.006 +36.704 +185.070
Na half-activation +5 mV -0.032 -0.032 +0.041
Na half-inactivation -5 mV -0.000 -0.000 +0.001
Na half-inactivation +5 mV +0.000 +0.000 -0.000
Leak density +20% -0.013 -0.021 +0.036
Leak density -20% +0.013 +0.021 -0.036
Leak reversal -5 mV -0.003 -0.003 +0.006
Leak reversal +5 mV +0.003 +0.002 -0.003
Ih multiplier +20% -0.007 -0.012 +0.025
Ih multiplier -20% +0.008 +0.014 -0.030
Kdr soma +20% -0.000 -0.000 +0.000
Kdr soma -20% +0.000 +0.000 -0.000
Kdr dendrites +20% -0.000 -0.000 +0.000
Kdr dendrites -20% +0.000 +0.000 -0.000
KM soma + dendrites +20% -0.013 -0.018 +0.032
KM soma + dendrites -20% +0.015 +0.020 -0.037
KA multiplier +20% -0.002 -0.005 +0.010
KA multiplier -20% +0.002 +0.005 -0.010
KA kinetic shift +5 mV +0.007 +0.014 -0.031
KA kinetic shift -5 mV -0.016 -0.032 +0.069
KA activation slope +20% -0.048 -0.094 +0.200
KA activation slope -20% +0.011 +0.022 -0.047
KD soma + dendrites +20% -0.003 -0.004 +0.007
KD soma + dendrites -20% +0.003 +0.004 -0.007
KD axon +20% -0.018 -0.019 +0.025
KD axon -20% +0.020 +0.022 -0.029
CaR permeability +20% +0.000 +0.000 -0.000
CaR permeability -20% -0.000 -0.000 +0.000
CaN permeability +20% +0.000 +0.000 -0.000
CaN permeability -20% -0.000 -0.000 +0.000
CaL multiplier +20% +0.000 +0.000 -0.000
CaL multiplier -20% -0.000 -0.000 +0.000
CaT multiplier +20% +0.002 +0.004 -0.006
CaT multiplier -20% -0.002 -0.004 +0.006
BK density +20% -0.000 -0.000 +0.000
BK density -20% +0.000 +0.000 -0.000
KAHP density +20% -0.000 -0.000 +0.000
KAHP density -20% +0.000 +0.000 -0.000
SK density +20% -0.005 -0.010 +0.021
SK density -20% +0.005 +0.010 -0.021
attenuation_soma/dend_100_um
attenuation_soma/dend_200_um
attenuation_soma/dend_300_um
PSP attenuation feature
0.04
0.02
0.00
−0.02
−0.04
Change in normalized feature error
(perturbed - baseline)

Supplementary Figure 12: Sensitivity of feature errors of the Postsynaptic Potential Attenuation Test of HippoUnit to variations in model parameters. This figure is analogous to Supplementary Figure 10, except that we clipped the color scale at -0.05 and +0.05 in this case. Lowering the half-activation of the sodium channels by 5 mV led to a spontaneously firing cell model, drastically increasing the error of the test, but the perturbations of other optimized parameters had relatively little effect on the results of this test.

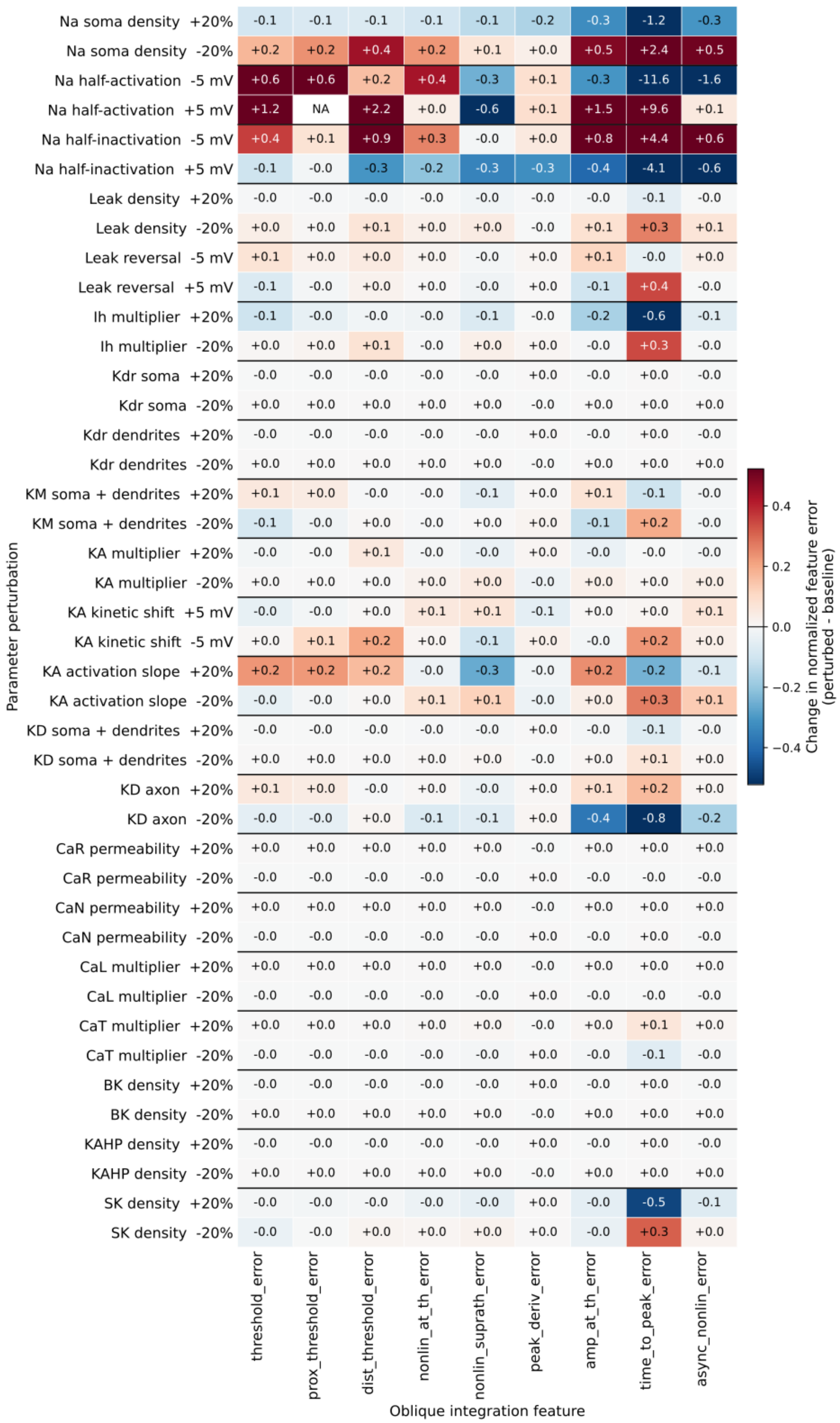
Na soma density +20% -0.1 -0.1 -0.1 -0.1 -0.1 -0.2 -0.3 -1.2 -0.3
Na soma density -20% +0.2 +0.2 +0.4 +0.2 +0.1 +0.0 +0.5 +2.4 +0.5
Na half-activation -5 mV +0.6 +0.6 +0.2 +0.4 -0.3 +0.1 -0.3 -11.6 -1.6
Na half-activation +5 mV +1.2 NA +2.2 +0.0 -0.6 +0.1 +1.5 +9.6 +0.1
Na half-inactivation -5 mV +0.4 +0.1 +0.9 +0.3 -0.0 +0.0 +0.8 +4.4 +0.6
Na half-inactivation +5 mV -0.1 -0.0 -0.3 -0.2 -0.3 -0.3 -0.4 -4.1 -0.6
Leak density +20% -0.0 -0.0 -0.0 -0.0 -0.0 -0.0 -0.0 -0.1 -0.0
Leak density -20% +0.0 +0.0 +0.1 +0.0 +0.0 -0.0 +0.1 +0.3 +0.1
Leak reversal -5 mV +0.1 +0.0 +0.0 +0.0 -0.0 +0.0 +0.1 -0.0 +0.0
Leak reversal +5 mV -0.1 -0.0 +0.0 +0.0 -0.0 +0.0 -0.1 +0.4 -0.0
Ih multiplier +20% -0.1 -0.0 -0.0 -0.0 -0.1 -0.0 -0.2 -0.6 -0.1
Ih multiplier -20% +0.0 +0.0 +0.1 -0.0 +0.0 +0.0 -0.0 +0.3 -0.0
Kdr soma +20% -0.0 -0.0 -0.0 -0.0 -0.0 +0.0 -0.0 +0.0 -0.0
Kdr soma -20% +0.0 +0.0 +0.0 +0.0 +0.0 -0.0 +0.0 +0.0 +0.0
Kdr dendrites +20% -0.0 -0.0 -0.0 -0.0 -0.0 +0.0 -0.0 +0.0 -0.0
Kdr dendrites -20% +0.0 +0.0 +0.0 +0.0 +0.0 -0.0 +0.0 +0.0 +0.0
KM soma + dendrites +20% +0.1 +0.0 -0.0 -0.0 -0.1 +0.0 +0.1 -0.1 -0.0
KM soma + dendrites -20% -0.1 -0.0 +0.0 -0.0 +0.0 +0.0 -0.1 +0.2 -0.0
KA multiplier +20% -0.0 -0.0 +0.1 -0.0 -0.0 +0.0 -0.0 -0.0 -0.0
KA multiplier -20% +0.0 +0.0 +0.0 +0.0 +0.0 -0.0 +0.0 +0.0 +0.0
KA kinetic shift +5 mV -0.0 -0.0 +0.0 +0.1 +0.1 -0.1 +0.0 +0.0 +0.1
KA kinetic shift -5 mV +0.0 +0.1 +0.2 +0.0 -0.1 +0.0 -0.0 +0.2 +0.0
KA activation slope +20% +0.2 +0.2 +0.2 -0.0 -0.3 -0.0 +0.2 -0.2 -0.1
KA activation slope -20% -0.0 -0.0 +0.0 +0.1 +0.1 -0.0 +0.0 +0.3 +0.1
KD soma + dendrites +20% -0.0 -0.0 -0.0 -0.0 -0.0 +0.0 -0.0 -0.1 -0.0
KD soma + dendrites -20% +0.0 +0.0 +0.0 +0.0 +0.0 -0.0 +0.0 +0.1 +0.0
KD axon +20% +0.1 +0.0 -0.0 +0.0 -0.0 +0.0 +0.1 +0.2 +0.0
KD axon -20% -0.0 -0.0 +0.0 -0.1 -0.1 +0.0 -0.4 -0.8 -0.2
CaR permeability +20% +0.0 +0.0 +0.0 +0.0 +0.0 -0.0 +0.0 +0.0 +0.0
CaR permeability -20% -0.0 -0.0 -0.0 -0.0 -0.0 +0.0 -0.0 -0.0 -0.0
CaN permeability +20% +0.0 +0.0 +0.0 +0.0 +0.0 -0.0 +0.0 +0.0 +0.0
CaN permeability -20% -0.0 -0.0 -0.0 -0.0 -0.0 +0.0 -0.0 +0.0 -0.0
CaL multiplier +20% +0.0 +0.0 +0.0 +0.0 +0.0 -0.0 +0.0 +0.0 +0.0
CaL multiplier -20% -0.0 -0.0 -0.0 -0.0 -0.0 +0.0 -0.0 -0.0 -0.0
CaT multiplier +20% +0.0 +0.0 +0.0 +0.0 +0.0 -0.0 +0.0 +0.1 +0.0
CaT multiplier -20% -0.0 -0.0 -0.0 -0.0 -0.0 +0.0 -0.0 -0.1 -0.0
BK density +20% -0.0 -0.0 -0.0 -0.0 -0.0 +0.0 -0.0 +0.0 -0.0
BK density -20% +0.0 +0.0 +0.0 +0.0 +0.0 -0.0 +0.0 +0.0 +0.0
KAHP density +20% -0.0 -0.0 -0.0 -0.0 -0.0 +0.0 -0.0 +0.0 -0.0
KAHP density -20% +0.0 +0.0 +0.0 +0.0 +0.0 -0.0 +0.0 +0.0 +0.0
SK density +20% -0.0 -0.0 -0.0 -0.0 -0.0 +0.0 -0.0 -0.5 -0.1
SK density -20% -0.0 -0.0 +0.0 +0.0 +0.0 +0.0 -0.0 +0.3 +0.0
Parameter perturbation
threshold_error
prox_threshold_error
dist_threshold_error
nonlin_at_th_error
nonlin_suprath_error
peak_deriv_error
amp_at_th_error
time_to_peak_error
async_nonlin_error
Oblique integration feature
0.4
0.2
0.0
−0.2
−0.4
Change in normalized feature error
(perturbed - baseline)

Supplementary Figure 13: Sensitivity of feature errors of the Oblique Integration Test of HippoUnit to variations in model parameters. This figure is analogous to Supplementary Figure 10, except that we clipped the color scale at -0.5 and +0.5 in this case. Once again, changing the properties and maximal conductance of the sodium channels had the largest effect on the results of this test. Changes in the time_to_peak feature errors are due to the altered timing of the action potentials at the soma. The errors of this feature are discussed further in the main article.

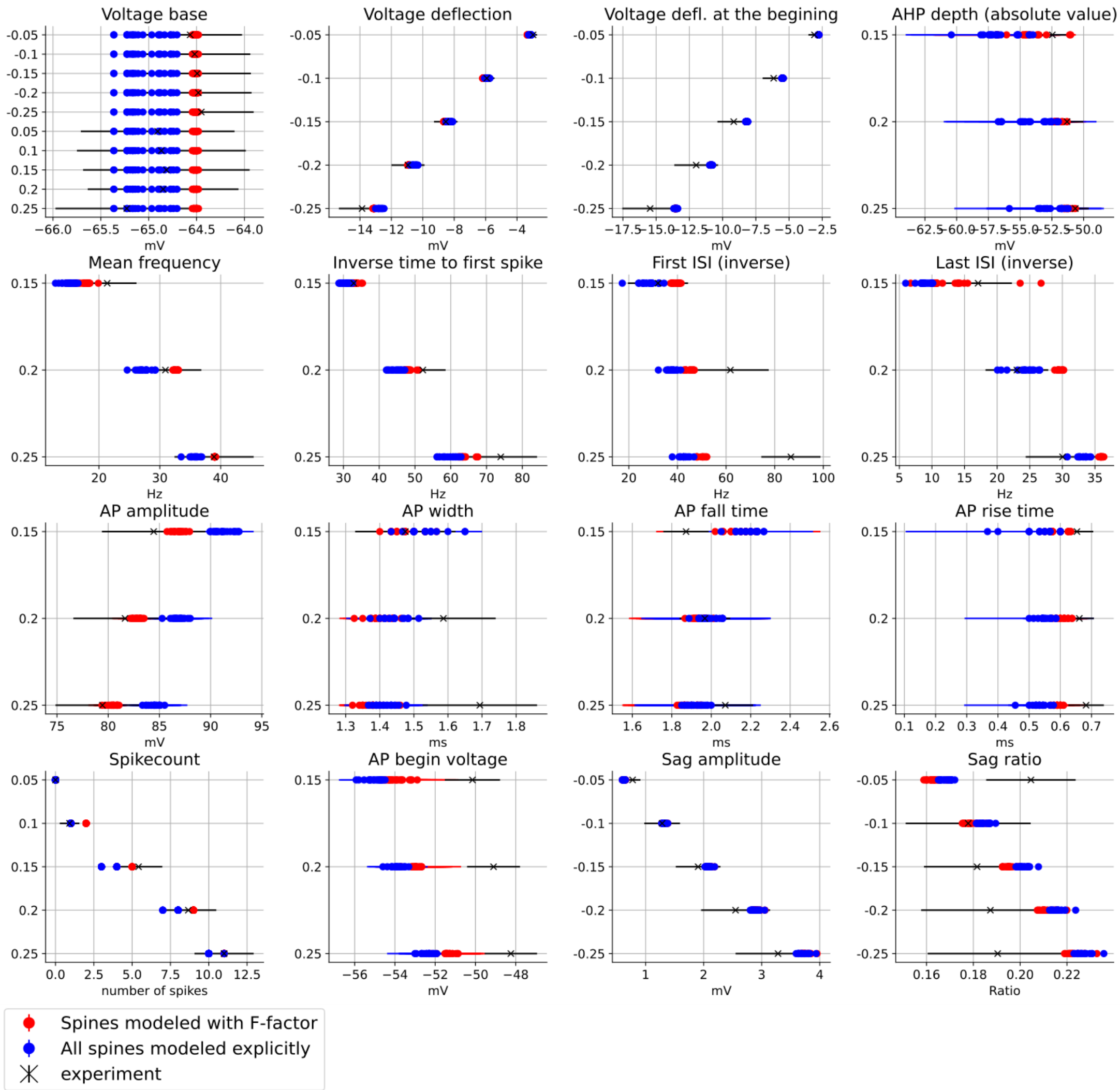

Supplementary Figure 14: The result of the Somatic Features Test of HippoUnit for the 20 optimized models, where the spines were modeled with the F-factor (red), and for the models (constructed from the same parameters) that had all their spines modelled explicitly (blue). For this figure 16 representative features were selected to show various aspects of the somatic behavior of the cells, from passive properties to action potential shapes and spiking patterns.

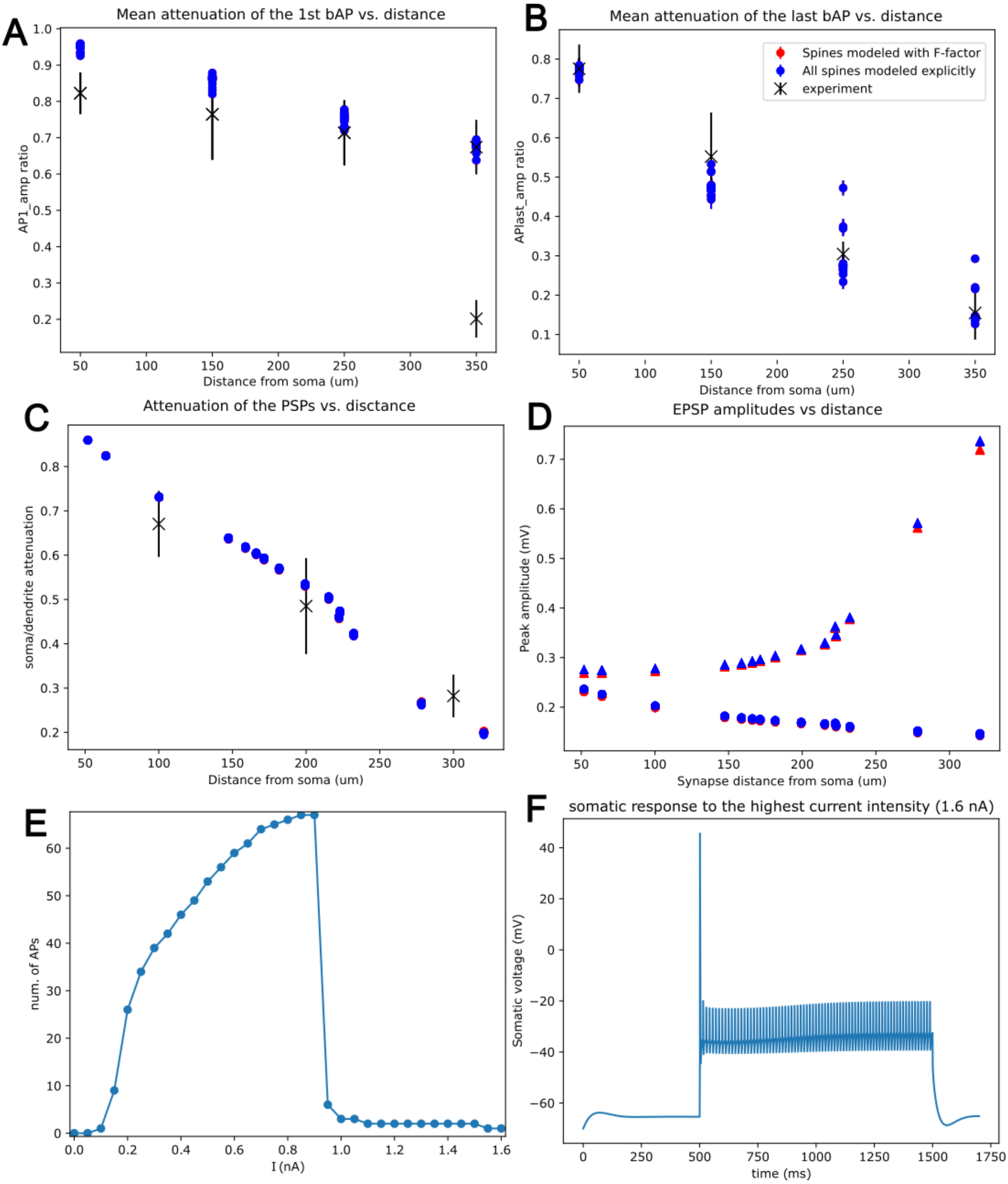

Supplementary Figure 15: This figure is analogous to Figure 4 but compares the models that have their spines taken into account with the F-factor method (shown with red symbols) and the models that have all their spines modeled explicitly (shown with blue symbols). In E and F the results of the Depolarization Block Test from the all-spine model are shown, and these are almost identical to the results of the optimized model (where spines were modeled with the F-factor) shown in Figure 4.

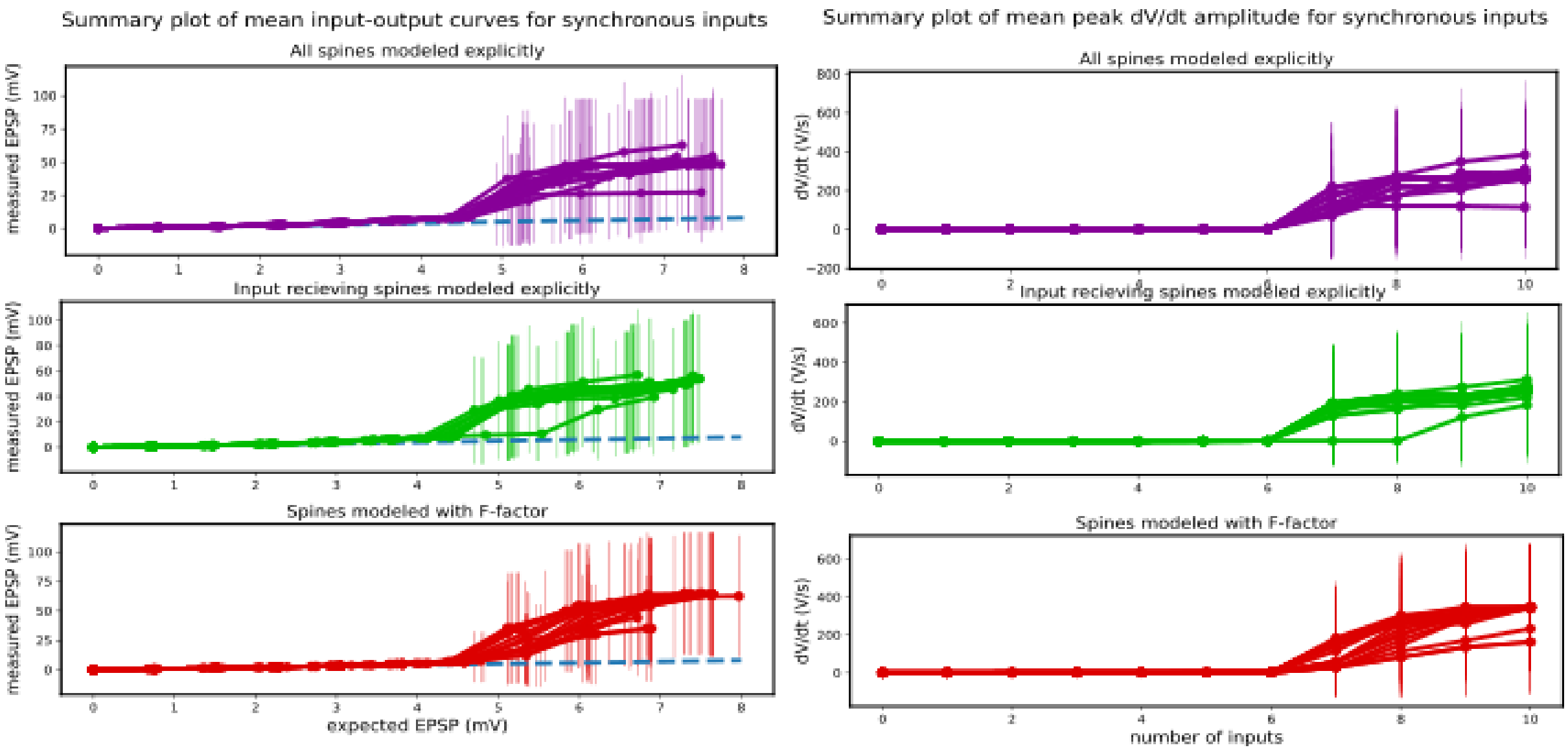

Supplementary Figure 16: Details of oblique integration with spines modeled at different levels of detail. The left panels show the actual EPSP amplitude vs. the EPSP amplitude expected in the case of linear summation of inputs, in three cases: all dendritic spines explicitly modeled (purple), only the spines that receive input explicitly modeled (green) and all spines modeled with the F-factor method, and therefore synapses arriving at the dendritic shaft (red). Each line shows the mean and standard deviation across the different dendrites of a model, and the lines represent models with different parameter sets. The right panels show summary plots of the peak dV/dt amplitudes in the same fashion.

**Model ID/Final score**	**Somatic Features Test**	**Backpropagating Action Potential Test**	**Post-synaptic Potential Attenuation Test**	**Depolarization Block Test**	**Oblique Integration Test**
**ca1_pc_1**	5.999812379380203	0.679974507948835	0.8815250528973664	100.0	1.3832827568140922
**ca1_pc_2**	6.102531665074244	0.6614106891395188	0.8783717689724702	100.0	1.4235210134301548
**ca1_pc_3**	6.137991297992989	0.8437047595192255	0.8864543368016756	100.0	1.3855369215806277
**ca1_pc_4**	6.010019314416906	0.6742835721659348	0.8791784298213062	100.0	1.393769446561066
**ca1_pc_5**	6.254036456606632	0.7804085657370539	0.8829035139849215	100.0	1.4106712002980333
**ca1_pc_6**	5.95653560340471	0.9294096683310361	0.8832031042049876	100.0	1.599708765450257
**ca1_pc_7**	6.3369241906772	0.7300643057414209	0.8824017011646402	100.0	1.4080181529433244
**ca1_pc_8**	5.808121596417921	0.708250468248414	0.8786129525874751	100.0	1.4613169925908647
**ca1_pc_9**	5.82354092561403	0.6708276520456713	0.8784880865127299	100.0	1.4632454938871824
**ca1_pc_10**	6.207260491835448	0.720940775691076	0.8815443231212111	100.0	1.439573421188216
**ca1_pc_11**	5.9764503220439895	0.6911989210428842	0.8806243973077268	100.0	1.4281206004197604
**ca1_pc_12**	6.047568039203012	0.8220784732196578	0.8832056100232343	100.0	1.4391154574667309
**ca1_pc_13**	6.26050956998024	0.7667380315646256	0.8802208985022135	100.0	1.437452833914895
**ca1_pc_14**	5.610302388775106	0.7837655488856184	0.8986955523636805	100.0	0.9032578564110012
**ca1_pc_15**	6.31275407603006	0.736670399951902	0.8858483339173863	100.0	1.3722673501887288
**ca1_pc_16**	5.5912158654555514	0.7482739740600358	0.896533365040896	100.0	0.9173685168476463
**ca1_pc_17**	5.843499945649421	0.6888593870201096	0.8787198786696456	100.0	1.4136129998454767
**ca1_pc_18**	6.192475750346506	0.72216791118272	0.8828033731446342	100.0	1.4019119882067568
**ca1_pc_19**	5.916793482120877	0.6706571100318572	0.8782966134552702	100.0	1.3948623959108088
**ca1_pc_20**	5.795548603332573	1.3051057901704874	0.8788489797720148	100.0	0.9090161076675553

Supplementary Table 1: The final score of the models on each HippoUnit test. Since none of the models entered depolarization block, they all got the error score of 100. On the other tests all models performed well, and they are very close to each other which indicates that the parameter fitting process found functionally similar solutions in all cases.
